# Supplementary material for: The brain microvasculature is a primary mediator of interferon-α neurotoxicity in human cerebral interferonopathies
Source: Immunity. 2024 Jul 9;57(7):1696–1709.e10. doi: 10.1016/j.immuni.2024.05.017 (PMC11250091; doi:10.1016/j.immuni.2024.05.017)
Supplement: Document S1. Figures S1–S7 and Tables S1 and S3–S5 [file mmc1.pdf]

Immunity, Volume *57*

Supplemental information

The brain microvasculature is a primary mediator of interferon-α neurotoxicity in human cerebral interferonopathies

Barney Viengkhou, Emina Hayashida, Sarah McGlasson, Katie Emelianova, Deborah Forbes, Stewart Wiseman, Joanna Wardlaw, Rovin Verdillo, Sarosh R. Irani, Darragh Duffy, Fredrik Piehl, Lipin Loo, Axel Pagenstecher, G. Greg Neely, Yanick J. Crow, Iain L. Campbell, David P.J. Hunt, and Markus J. Hofer

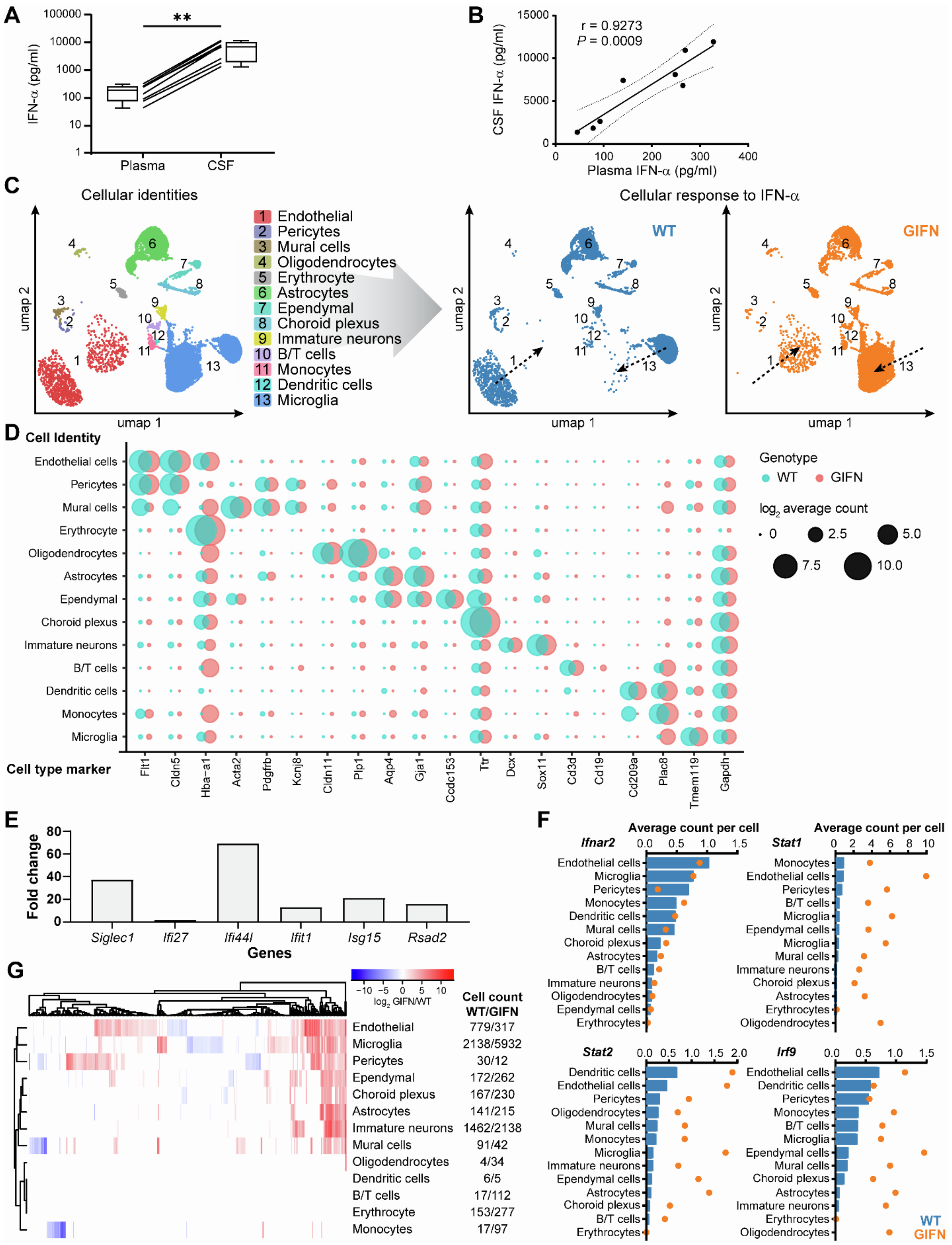

A
IFN-α (pg/ml)
Plasma
CSF
**
B
r = 0.9273
P = 0.0009
CSF IFN-α (pg/ml)
Plasma IFN-α (pg/ml)
C
Cellular identities
1 Endothelial
2 Pericytes
3 Mural cells
4 Oligodendrocytes
5 Erythrocyte
6 Astrocytes
7 Ependymal
8 Choroid plexus
9 Immature neurons
10 B/T cells
11 Monocytes
12 Dendritic cells
13 Microglia
Cellular response to IFN-α
WT
GIFN
umap 1
umap 2
D
Cell Identity
Genotype
WT
GIFN
log2 average count
Cell type marker
E
Fold change
Genes
Siglec1
Ifi27
Ifi44l
Ifit1
Isg15
Rsad2
F
Average count per cell
Ifnar2
Stat1
Stat2
Irf9
G
log2 GIFN/WT
Cell count WT/GIFN
Endothelial 779/317
Microglia 2138/5932
Pericytes 30/12
Ependymal 172/262
Choroid plexus 167/230
Astrocytes 141/215
Immature neurons 1462/2138
Mural cells 91/42
Oligodendrocytes 4/34
Dendritic cells 6/5
B/T cells 17/112
Erythrocyte 153/277
Monocytes 17/97

Fig. S1 CSF IFN-α concentrations and type I interferon signaling are increased in brains of GIFN mice (Fig. S1 refers to Fig. 2).

(**A**) Paired comparison of CSF and plasma samples IFN-α protein from GIFN mice (*n* = 8) measured by ELISA. Samples from WT mice (*n* = 4) were below the detection limit of the ELISA. Box plots are shown. Paired two-tailed t test. $^{**}P < 0.01$.

(**B**) Correlation between CSF and plasma IFN-α protein in GIFN mice. Pearson correlation with 95% confidence bands.

(**C**) UMAP plot showing clustering of cells in of WT and GIFN mice. Data from one experiment.

(**D**) Known cell-type-associated markers were used to assign and verify cell identities of clusters.

(**E**) Fold change (GIFN vs WT) of genes used to calculate the whole brain ISG score.

(**F**) Average count of transcripts of each cell identity and between WT and GIFN cells. *Ifnar1* is shown in Fig. 2.

(**G**) Heatmap of type I interferon-regulated genes from each cell identity.

A-B: Representative data from two independent experiments. **C-G**: Analyzed data from one experiment.

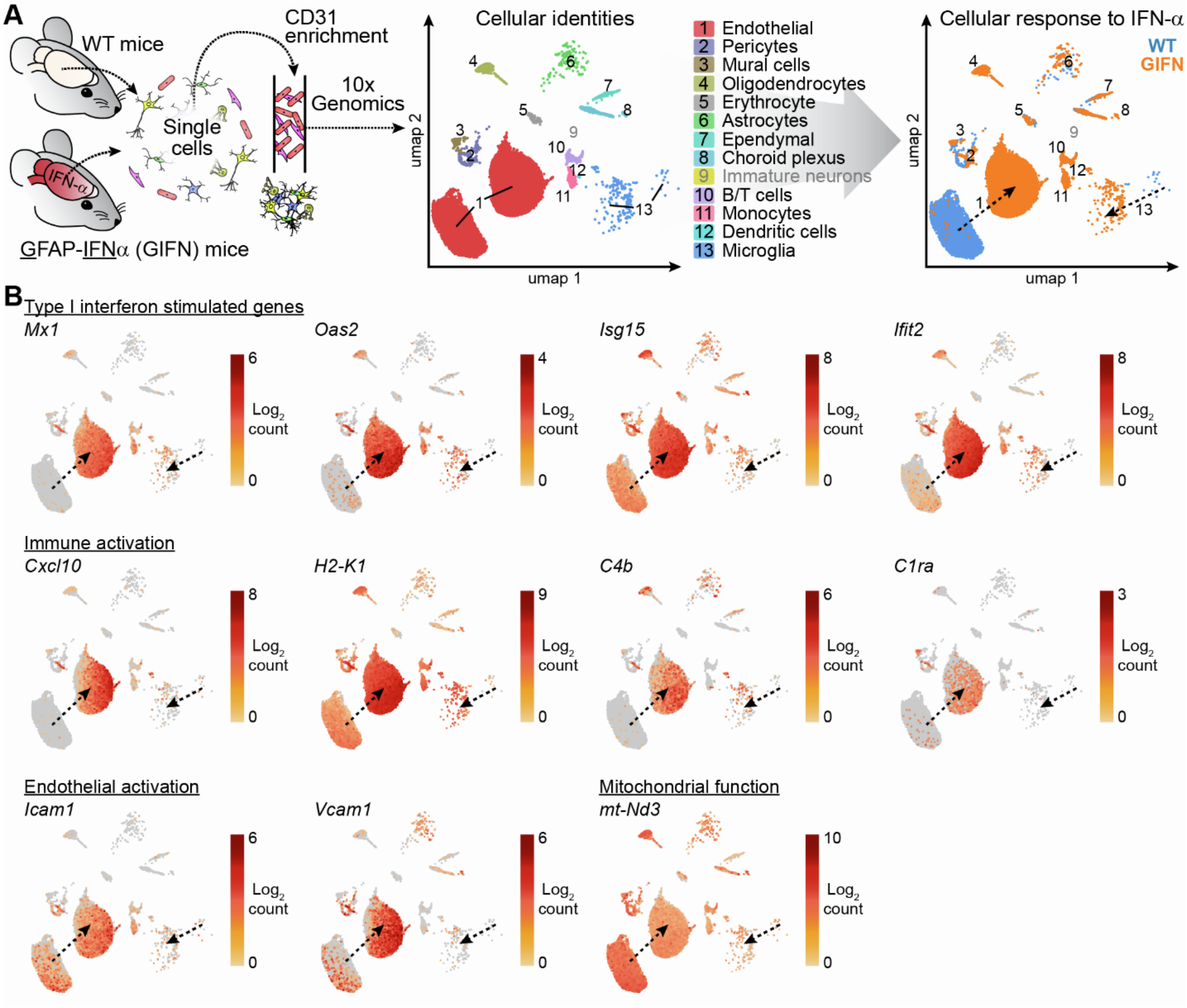

Fig. S2. Immune stimulation and endothelial cell activation is present in CD31-enriched cortical cells from GIFN mice versus WT mice (Fig. S2 refers to Fig. 3A-B).

(**A**) Single-cell RNA sequencing of isolated cells from pooled forebrains of WT and GIFN mice that were enriched using CD31-microbeads. Cells were clustered and assigned an identity which allowed transcriptomic comparison between the two genotypes, as in Fig. 2. Note, immature neurons were not detected. Dashed arrows indicate shift in endothelial cells and microglia between WT and GIFN mice.

(**B**) Markers of canonical type I interferon-stimulated genes, immune activation, endothelial activation and mitochondrial function.

A-B: Data from one experiment.

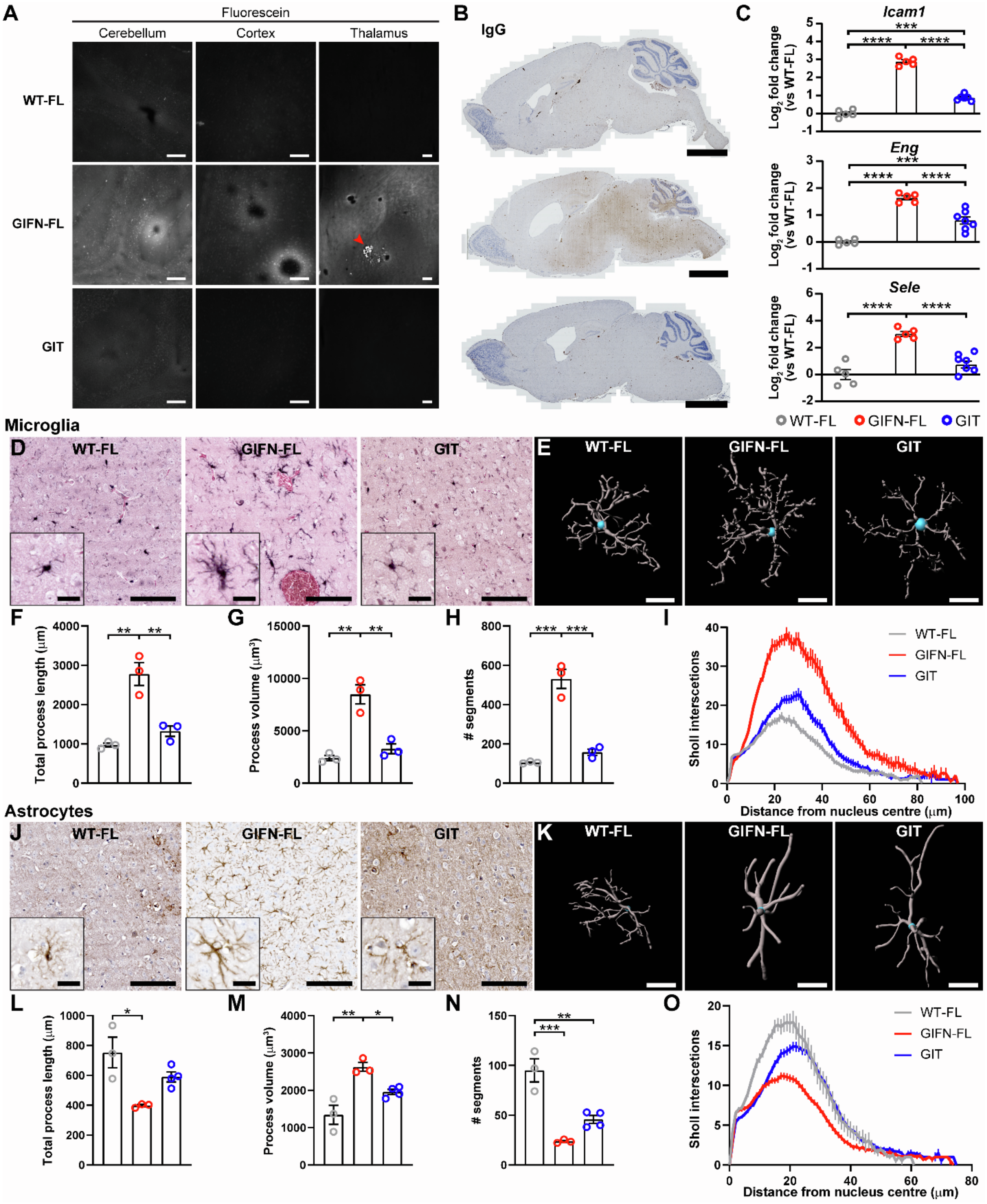

A
Fluorescein
Cerebellum
Cortex
Thalamus
WT-FL
GIFN-FL
GIT
B
IgG
C
Icam1
Eng
Sele
Log2 fold change (vs WT-FL)
WT-FL
GIFN-FL
GIT
Microglia
D
E
F
Total process length (μm)
G
Process volume (μm3)
H
segments
I
Sholl interscetions
Distance from nucleus centre (μm)
Astrocytes
J
K
L
Total process length (μm)
M
Process volume (μm3)
N
segments
O
Sholl interscetions
Distance from nucleus centre (μm)

Fig. S3. Blood-brain barrier function is restored and morphological characteristics of cortical microglia and astrocytes is partially reversed in GIT mice (Fig. S3 refers to Fig. 6).

(**A)** Representative fluorescent images of brain sections for fluorescein revealing the spatial leakage (*n* = 4 per genotype at 16 weeks of age). Red arrowhead: calcification; scale bar, 100 µm. Representative images from over three independent experiments.

(**B**) Immunohistochemistry for IgG (brown) in the parenchyma (*n* = 4 per genotype at 16 weeks of age; scale bar, 2 mm; hematoxylin counterstain). Representative images from two independent experiments.

(**C**) Markers of endothelial activation in the cerebellum measured by qPCR (*n* = 5 for WT-FL and GIFN-FL and *n* = 7 for GIT). Mean ± s.e.m. are shown. One-way ANOVA with Tukey's post-test. ***P* < 0.01, ****P* < 0.001 and *****P* < 0.0001. Quantification was one experiment from sample collected from more than three independent experiments.

(**D** and **J**) Immunohistochemistry for microglia (Iba1) and astrocytes (GFAP) (*n* = 4 per genotype at 16 weeks of age; scale bar, 100 µm and 20 µm for inserts). Representative images from two independent experiments.

(**E** and **K**) Reconstructed morphology from Iba1 and GFAP stained passively cleared brains (*n* = 3-4 mice per genotype at 16 weeks of age). Representative images from two independent experiments.

(**F** and **L**) Total length of processes calculated from morphological reconstructions.

(**G** and **M**) Average volume of processes calculated from single cell morphological reconstructions.

(**H** and **N**) Number of segments calculated from single cell morphological reconstructions.

(**I** and **O**) Histogram of Sholl intersections calculated from single cell morphological reconstructions.

C, **F**-**H** and **L**-**N**: each point is a mouse. **I** and **O** are from of all measured microglia and astrocytes. Mean ± s.e.m. are shown. Statistical comparisons were performed using one-way ANOVA with Tukey's post-test. **P* ≤ 0.05, ***P* ≤ 0.01, ****P* ≤ 0.001 and *****P* ≤ 0.0001. **E**-**I** and **K**-**O**: Quantification of data pooled from two independent experiments.

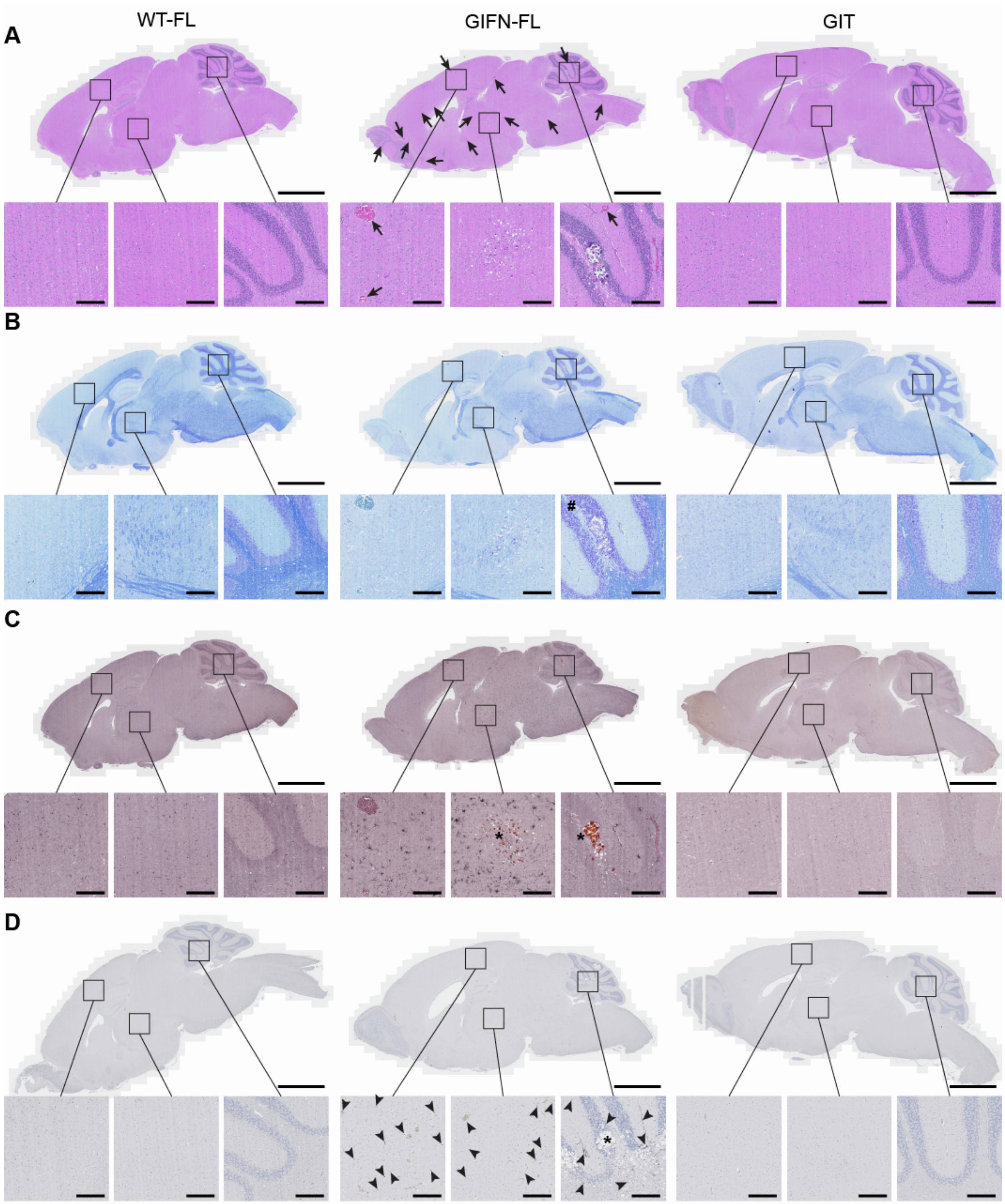

Fig. S4. Microangiopathy, neurodegeneration, calcification and T cell infiltrates are absent in the brains of 16-week-old GIT mice (Fig. S4 refers to Fig. 7A).

(**A**) H&E (arrows: aneurysms).

(**B**) LFB&CV (#: loss of myelin).

(**C**) Iba1-ARS (*: calcification).

(**D**) CD3 (arrowheads) staining.

Staining of of WT-FL, GIFN-FL and GIT mice at 16 weeks of age (*n* = 4 per genotype). Whole brain image: scale bar, 2 mm; zoomed images: scale bar, 200 μm. Representative images from two independent experiments.

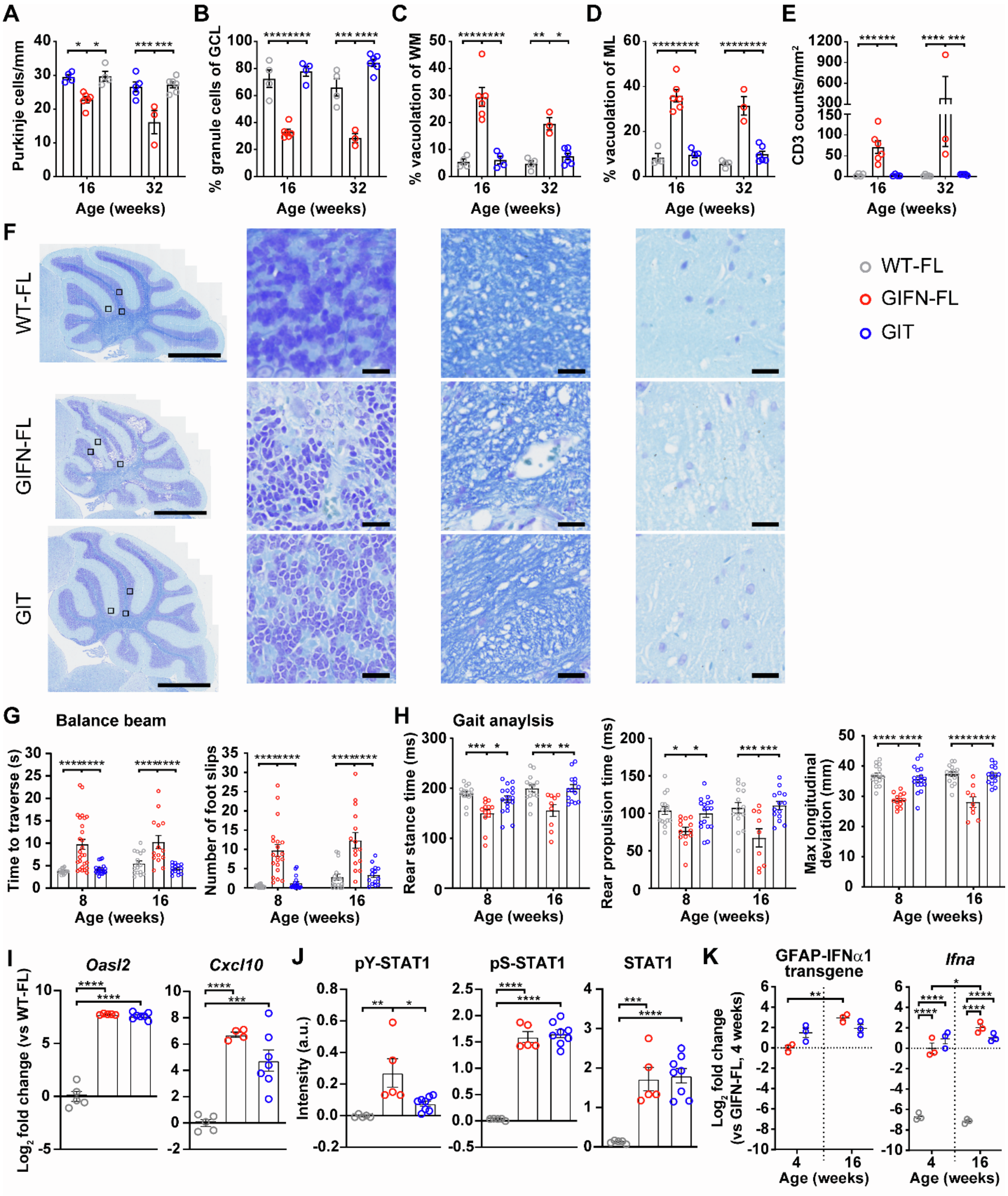

Fig. S5. Neuropathology and motor phenotype are improved in GIT mice despite chronic type I interferon signaling in the brain (Fig S5 refers to Fig. 7).

(**A**) Purkinje cells normalized to the perimeter of the granule cell layer (GCL) of the cerebellum.

(**B**) Percentage area granule cells in the GCL of the cerebellum.

(**C**) Percentage area vacuolation of the white matter (WM) of the cerebellum.

(**D**) Percentage area vacuolation of the molecular layer (ML) of the cerebellum.

(**E**) Number of CD3 cells per mm^2 of the cerebellum.

(**F**) Representative low (scale bar, 1 mm) and high magnification (scale bar, 20 μm) of the cerebellum indicating the GCL, WM and ML in WT-FL, GIFN-FL and GIT mice at 16 weeks of age (*n* = 4 per genotype).

(**G**) Fine motor coordination detected using the balance beam test (*n* = 16 for WT-FL, 25 for GIFN-FL and 18 for GIT at 8 weeks of age; 16 for WT-FL, 12 for GIFN-FL and 14 for GIT at 16 weeks of age; repeated measures linear mixed-effects models with Tukey's post-test; also viewed in Video S2).

(**H**) Parameters of gait of average duration in which rear paws are in contact with the treadmill, average time it takes to leave the stance phase and average maximum distance the foot deviates from the waist during the stance phase (*n* = 15 for WT-FL, 13 for GIFN-FL and 17 for GIT at 8 weeks of age; 14 for WT-FL, 10 for GIFN-FL and 14 for GIT at 16 weeks of age; repeated measures linear mixed-effects models with Tukey's post-test).

(**I**) Expression of type I interferon-stimulated genes in the cerebella of mice measured by qPCR (*n* = 4-5 for WT-FL and GIFN-FL and 7 for GIT at 16 weeks of age; one-way ANOVA with Tukey's post-test).

(**J**) Quantification of immunoblots from Fig. 7D (*n* = 5 for WT-FL and GIFN-FL and 8 for GIT at 16 weeks of age; one-way ANOVA with Tukey's post-test).

(**K**) Expression of the transgene (GFAP-IFNα1) and *Ifna* in the cerebellum measured by qPCR (*n* = 3 per genotype per age; two-way ANOVA with Tukey's post-test). Representative analysis from two independent experiments.

Points represent individual animals and mean ± s.e.m. shown. $^*P < 0.05$, $^{**}P < 0.01$, $^{***}P < 0.001$ and $^{****}P < 0.0001$. **A**-**E**: *n* = 3-5 per genotype per age. Two-way ANOVA with Tukey's post-test. **A**-**G**: Quantification from images obtained in two independent experiments. **F**: Representative images from two independent experiments. **G**-**H**: Data pooled from more than three independent experiments. **I**-**J**: Quantification was one experiment from sample collected from more than three independent experiments.

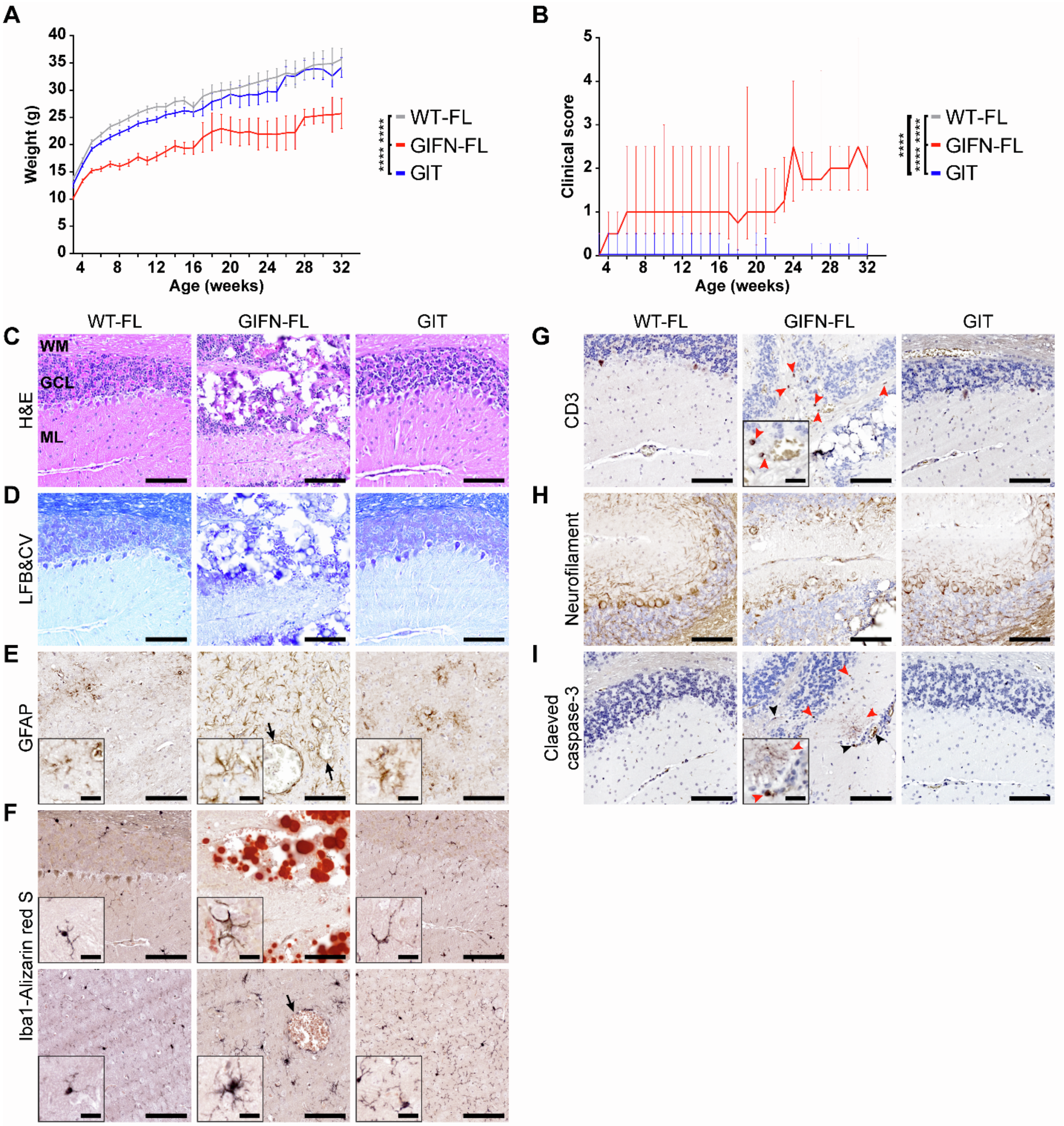

Fig. S6. GIT mice have a largely WT-like development and neuropathology (Fig S6 refers to Fig. 7).

(**A**) Weight curve of mice up to 32 weeks of age (*n* = 50-57 mice per genotype). Mean ± s.e.m. shown.

(**B**) Clinical score (median ±interquartile range shown) indicating development of ataxia (score: 0.5 – 2), seizures (score: 3 – 4) and mortality (score: 5). *n* = 50-57 mice per genotype. Significance of clinical scores between genotypes was calculated with cumulative link mixed models with *P* adjusted with Tukey's post-test. Significance of weight between

genotypes was calculated with linear mixed-effects models and *P* adjusted with Tukey's post-test. ****$P \leq 0.0001$.

(**C**) H&E staining (white matter (WM), granule cell layer (GCL) and molecular layer (ML)).

(**D**) LFB&CV staining.

(**E**) GFAP immunohistochemistry.

(**F**) Staining for Iba1 and Alizarin red S.

(**G**) CD3 staining showed T cell infiltration (red arrowheads). Black arrowheads indicate perivascular cells and red arrow heads indicate positive cells in the parenchyma.

(**H**) Neurofilament staining.

(**I**) Cleaved caspase-3 staining showed apoptosis.

A-**B**: Data pooled from more than three independent experiments. **C**-**I**: n = 3-4 per genotype at 32 weeks of age. Scale bar, 100 μm and 20 μm in inserts. Representative images from two independent experiments.

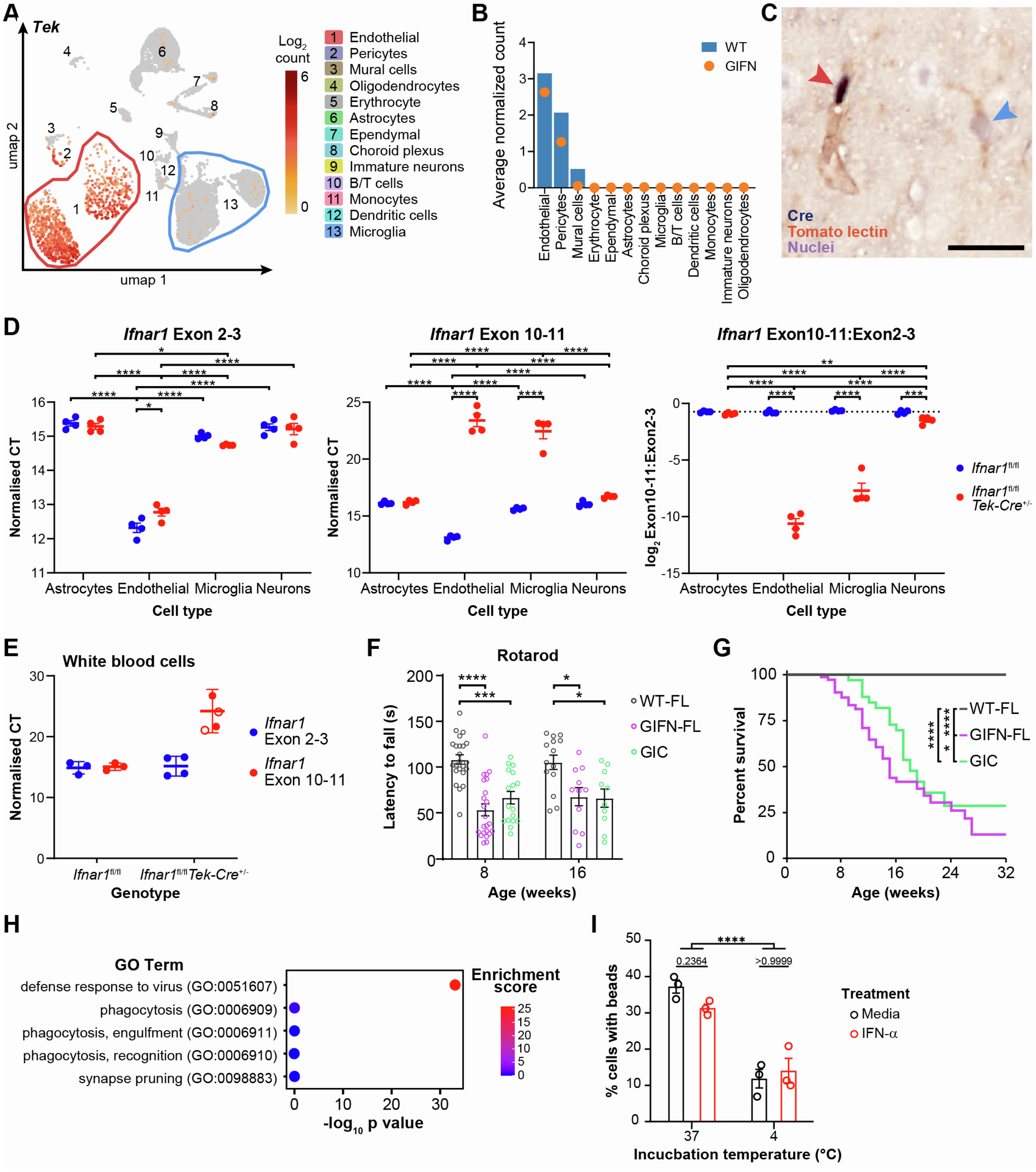

Fig. S7. *Ifnar1* deletion on microglia does not rescue clinical phenotype in GIFN mice (Fig S7 refers to Figs. 6 & 7).

(**A**) scRNASeq data from WT and GIFN mice showing *Tek* expression.

(**B**) Average normalized counts of *Tek* for each cell identity.

(**C**) Immunohistochemistry for Cre recombinase protein and tomato lectin (labels endothelial cells and microglia). Endothelial cell nuclei with Cre recombinase (red arrowhead), but

not in microglia (blue arrowhead). Scale bar, 20 µm. Representative images from two independent experiments.

(**D** and **E**) Expression of *Ifnar1* mRNA in (**D**) different brain cell types from *Ifnar1*$^{fl/fl}$ and *Ifnar1*$^{fl/fl}$*xTek-Cre*$^{+/-}$ mice (n = 4 per genotype) and (**E**) in white blood cells (n = 3-4 per genotype) measured by qPCR. Primers amplifying across exons 2 and 3 indicate levels of *Ifnar1* transcripts and those amplifying across exons 10 and 11 indicate transcripts from cells without exon 10 excision. Transcripts were normalized to 18S. Mean ± s.e.m. and two-way ANOVA with Tukey's post-test are shown in (**D**) and mean ± s.d. in (**E**). Empty dots in (**E**) were not included in the mean but indicate undetermined CTs that were set to a CT value of 40. **D**: Data were from two independent experiment. **E**: Data from one experiment.

(**F**) Gross motor function measured by rotarod test (n = 23 for WT-FL, 23 for GIFN-FL and 17 for GIC at 8 weeks and n = 14 for WT-FL, 11 for GIFN-FL and 10 for GIC at 16 weeks; repeated measures linear mixed-effects models with Tukey's post-test. Quantification of data pooled from more than three independent experiments.

(**G**) Survival analysis of mice with *Ifnar1* excision in microglia in GIFN mice (GIC) and control littermates (total n = 96 for WT-FL, 82 for GIFN-FL and 47 for GIC mice). Significance determined by long-rank test with Benjamini-Hochberg post-test. Data pooled from more than three independent experiments.

(**H**) Overrepresentation analysis for phagocytosis associated biological processes in microglia from the GIFN brain using PANTHER.

(**I**) Phagocytosis assay using fluorescent latex beads incubated for 1 h at 4°C or 37°C with primary microglia pretreated with media or IFN-α for 72 h (n = 3 per treatment per temperature). Mean ± s.e.m. and two-way ANOVA with Tukey's post-test. Representative analysis from two independent experiments.

A-B and **H**: Data from one experiment. *$P < 0.05$, **$P < 0.01$, ***$P < 0.001$ and ****$P <$ 0.0001.

Table S1. IFN-α concentrations in paired serum-CSF across diseases (Table S1 refers to Fig 1A-D).

	**Controls**		**Interferonopathic Disease**	
	**Healthy Control**	**Disease Control (Multiple Sclerosis – a non-interferonopathic disease)**	**Cerebral Interferonopathy disease (AGS)**	**Systemic Interferonopathy disease (SLE)**
**Number of serum-CSF pairs**	19	32	29	53
**Serum IFN-α fg/ml (median and IQR)**	0.36 (0.36 - 0.40)	0.36 (0.36 - 0.62)	153 (39 - 622)	195 (9.9 - 770)
**CSF IFN-α fg/ml (median and IQR)**	1.74 (1.1 - 4.1)	0.81 (0.72 - 2.1)	552 (104 - 3189)	7.3 (1.5 - 38.5)

Table S3. Multiple linear regression analysis with "age at sampling" as the dependent variable (Table S3 refers to Fig. 5A).

Parameter estimates	Variable	Estimate	|t|	P value	VIF	R2 with other variables
β0	Intercept	38.16	7.486	<0.0001		
β1	CSF interferon (IU/ml)	-0.2281	3.628	0.0004	1.085	0.07818
β2	Serum interferon (IU/ml)	-0.08033	0.7658	0.4456	1.085	0.07818

Table S4. Cohort demographics for human samples.

A) Cohort demographic for paired serum-CSF Simoa study (Table S4A refers to Fig 1).

B) Cohort demographic for SLE paired Simoa – neuroimaging/MRI study (Table S4B refers to Fig 5).

Table S4A.

		Non-interferonopathic controls		Interferonopathic disease		
Underlying diagnosis		Healthy controls	Relapsing-remitting multiple sclerosis	Systemic lupus erythematosus cohort 1	Systemic lupus erythematosus cohort 2	Aicardi-Goutières Syndrome
Number of serum-CSF pairs		19	32	35	18	29
Age	Mean age	27.4	34.5	45	15	9
	Age range	22-32	20-51	18-77	11 to 17	0-38
Sex	Female %	53	78	97	73	54
Ethnicity	White Caucasian %	89	84	86	N/A	N/A
	Asian %	11	16	14	N/A	N/A
Country of origin	Sweden %	78	72	80	N/A	N/A
	Other European %	11	6.00	9	N/A	N/A
	Middle East %	11	16	9	N/A	N/A
	Other %		6	2	N/A	N/A

Table S4B.

Diagnosis		Systemic lupus erythematosus
	n	47
Age	Mean age	49
	Age range	20-75
Sex	Female %	91
Ethnicity	White Caucasian %	93
	Other %	7
Socioeconomic status	School leaving age	16 years

Table S5. List of oligonucleotides (Table S5 refers to STAR Methods).

Oligonucleotide	Source	Identifier
Expression assays		
Icam1 forward primer: 5'-CTGGGCTTGGAGACTCAGTG-3'	Sigma-Aldrich	N/A
Icam1 reverse primer: 5'-CACACTCTCCGGAAACGAA-3'	Sigma-Aldrich	N/A
Eng forward primer: 5'-AGGTGTTCCTGGTCCTCGTT-3'	Sigma-Aldrich	N/A
Eng reverse primer: 5'-GTTGACTCTTGGCTGTCCTTG-3'	Sigma-Aldrich	N/A
Sele forward primer: 5'-CTAGCGCCTGGATGAAAGC-3'	Sigma-Aldrich	N/A
Sele reverse primer: 5'-GAGCTCACTGGAGGCATTGT-3'	Sigma-Aldrich	N/A
Isg15 forward primer: 5'-GAGCTAGAGCCTGCAGCAAT-3'	Sigma-Aldrich	N/A
Isg15 reverse primer: 5'-TTCTGGGCAATCTGCTTCTT-3'	Sigma-Aldrich	N/A
Mx1 forward primer: 5'-TCTGAGGAGAGCCAGACGAT-3'	Sigma-Aldrich; Hernández et al. [54]	N/A
Mx1 reverse primer: 5'-ACTCTGGTCCCCAATGACAG-3'	Sigma-Aldrich; Hernández et al. [54]	N/A
Oasl2 forward primer: 5'-GGATGCCTGGGAGAGAATCG-3'	Sigma-Aldrich; Hernández et al. [54]	N/A
Oasl2 reverse primer: 5'-TCGCCTGCTCTTCGAAAC-3'	Sigma-Aldrich; Hernández et al. [54]	N/A
Cxcl10 forward primer: 5'-AGAGACATCCCGAGCCAA-3'	Sigma-Aldrich	N/A
Cxcl10 reverse primer: 5'-GATGAGGCAGAAAATGACGG-3'	Sigma-Aldrich	N/A
Ifna forward primer: 5'-GTGACCTTCCTCAGACTCATAAC-3'	Sigma-Aldrich	N/A
Ifna reverse primer: 5'-CAAAGTCCTTCCTGTCCTTCA-3'	Sigma-Aldrich	N/A
GFAP-IFNα1 transgene forward primer: 5'-CAATGTGCTGGGAAGACTGA-3'	Sigma-Aldrich	N/A
GFAP-IFNα1 transgene reverse primer: 5'-CTGCATTCTAGTTGTGGTTTGTC-3'	Sigma-Aldrich	N/A
Ifnar1 Exon 2 forward primer: 5'-CAGCCACGGAGAGTCAATG-3'	Sigma-Aldrich	N/A
Ifnar1 Exon 3 reverse primer: 5'-GCACTTTTAACCACTTCGCC-3'	Sigma-Aldrich	N/A
Ifnar1 Exon 10 forward primer: 5'-GCCTCCCCGCAGTATTGAT-3'	Sigma-Aldrich	N/A

Ifnar1 Exon 11 reverse primer: 5’-ATCTTTCCGTGTGCTCCTCA-3’	Sigma-Aldrich	N/A
18S forward primer: 5’-CACGGCCGGTACAGTGAAAC-3'	Sigma-Aldrich; Hernández et al. [54]	N/A
18S reverse primer: 5’-AGAGGAGCGAGCGACCAA-3'	Sigma-Aldrich; Hernández et al. [54]	N/A
Genotyping assays		
Ifnar1 Exon 10 forward primer: 5’-GTGTTGTGTTCTTCTCTGTCATGGT-3’	Sigma-Aldrich	N/A
Ifnar1 Exon 10 reverse primer: 5’-AATACTGCGGGGAGGCTTGA-3’	Sigma-Aldrich	N/A
Tk1 forward primer: 5’-CCAACGAGGGCAAGACAGTAATTG-3’	Sigma-Aldrich	N/A
Tk1 reverse primer: 5’-AGAGCACCCACTAGCCAATAGC-3’	Sigma-Aldrich	N/A
