# Supplementary material for: The brain microvasculature is a primary mediator of interferon-α neurotoxicity in human cerebral interferonopathies
Source: Immunity. 2024 Jul 9;57(7):1696–1709.e10. doi: 10.1016/j.immuni.2024.05.017 (PMC11250091; doi:10.1016/j.immuni.2024.05.017)
Supplement: Document S2. Article plus supplemental information [file mmc5.pdf]

Article

Immunity

The brain microvasculature is a primary mediator of interferon-α neurotoxicity in human cerebral interferonopathies

Graphical abstract

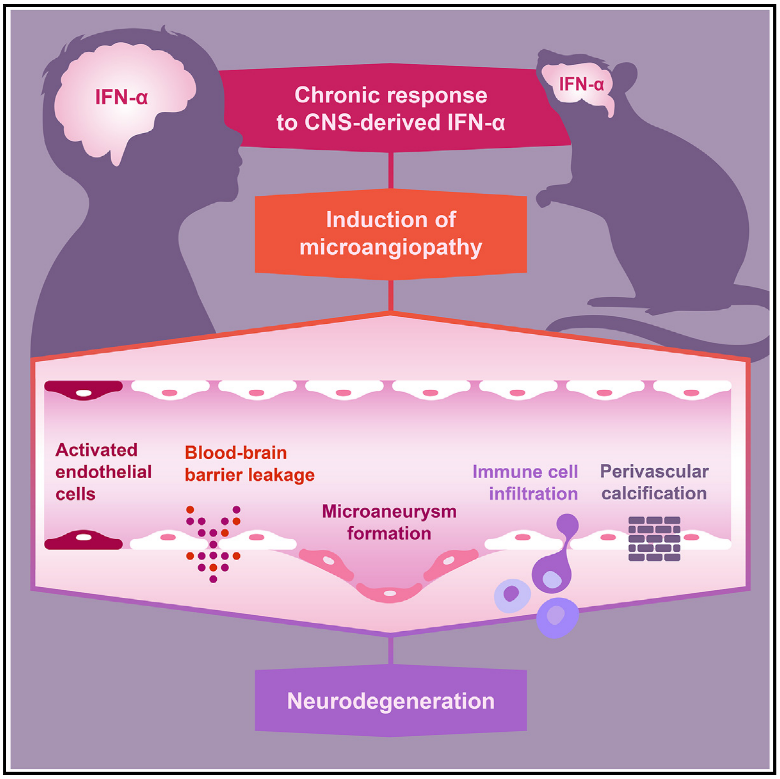

Highlights

- Neurotoxic IFN-α in Aicardi-Goutières syndrome (AGS) is primarily of cerebral origin
- Endothelial cells are critical cellular targets of neurotoxic IFN-α
- Chronically elevated intracerebral IFN-α causes a distinct cerebral microangiopathy
- Neurodegeneration arises as a secondary consequence of IFN-α-driven microangiopathy

Authors

Barney Viengkhou, Emina Hayashida, Sarah McGlasson, ..., Iain L. Campbell, David P.J. Hunt, Markus J. Hofer

Correspondence

david.hunt@ed.ac.uk (D.P.J.H.), markus.hofer@sydney.edu.au (M.J.H.)

In brief

Aicardi-Goutières syndrome is an autoinflammatory brain disease associated with increased interferon-α production, but the primary source and mediators of neurotoxicity are poorly described. Viengkhou et al. show that neurotoxic interferon-α originates in the central nervous system and mediates disease primarily through its effects on the cerebral microvasculature. This represents an accessible target for therapeutic intervention.

Check for updates

Viengkhou et al., 2024, Immunity 57, 1696–1709
July 9, 2024 © 2024 The Authors. Published by Elsevier Inc.
https://doi.org/10.1016/j.immuni.2024.05.017

CellPress

CellPress
OPEN ACCESS

Immunity

Article

The brain microvasculature is a primary mediator of interferon-α neurotoxicity in human cerebral interferonopathies

Barney Viengkhou,[1] Emina Hayashida,[1] Sarah McGlasson,[2,3] Katie Emelianova,[2] Deborah Forbes,[2,3] Stewart Wiseman,[3] Joanna Wardlaw,[2,3] Rovin Verdillo,[1] Sarosh R. Irani,[4] Darragh Duffy,[5] Fredrik Piehl,[6] Lipin Loo,[1] Axel Pagenstecher,[7] G. Greg Neely,[1] Yanick J. Crow,[8,9] Iain L. Campbell,[1] David P.J. Hunt,[2,3,10,*] and Markus J. Hofer[1,10,11,*]

[1]School of Life and Environmental Sciences and the Charles Perkins Centre, The University of Sydney, Sydney, NSW 2006, Australia
[2]UK Dementia Research Institute at University of Edinburgh, Edinburgh EH16 4SB, UK
[3]Centre for Clinical Brain Sciences at University of Edinburgh, Edinburgh EH16 4SB, UK
[4]Oxford Autoimmune Neurology Group, University of Oxford, Oxford, UK
[5]Translational Immunology Unit, Institut Pasteur, Université Paris Cité, Paris, France
[6]Neuroimmunology Unit, Department of Clinical Neuroscience, Karolinska Institutet, Stockholm, Sweden
[7]Department of Neuropathology, University of Marburg, Baldingerstrasse, 35043 Marburg, Germany
[8]MRC Human Genetics Unit, Institute of Genetics and Cancer, University of Edinburgh, Edinburgh, UK
[9]Laboratory of Neurogenetics and Neuroinflammation, Institut Imagine, Université de Paris, Paris, France
[10]Senior author
[11]Lead contact
*Correspondence: david.hunt@ed.ac.uk (D.P.J.H.), markus.hofer@sydney.edu.au (M.J.H.)
https://doi.org/10.1016/j.immuni.2024.05.017

SUMMARY

Aicardi-Goutières syndrome (AGS) is an autoinflammatory disease characterized by aberrant interferon (IFN)-α production. The major cause of morbidity in AGS is brain disease, yet the primary source and target of neurotoxic IFN-α remain unclear. Here, we demonstrated that the brain was the primary source of neurotoxic IFN-α in AGS and confirmed the neurotoxicity of intracerebral IFN-α using astrocyte-driven *Ifna1* misexpression in mice. Using single-cell RNA sequencing, we demonstrated that intracerebral IFN-α-activated receptor (IFNAR) signaling within cerebral endothelial cells caused a distinctive cerebral small vessel disease similar to that observed in individuals with AGS. Magnetic resonance imaging (MRI) and single-molecule ELISA revealed that central and not peripheral IFN-α was the primary determinant of microvascular disease in humans. Ablation of endothelial *Ifnar1* in mice rescued microvascular disease, stopped the development of diffuse brain disease, and prolonged lifespan. These results identify the cerebral microvasculature as a primary mediator of IFN-α neurotoxicity in AGS, representing an accessible target for therapeutic intervention.

INTRODUCTION

Aicardi-Goutières syndrome (AGS) is a devastating childhood disease with innate immune activation and aberrant production of interferon-alpha (IFN-α). AGS is characterized by congenital/early-onset brain disorder with prominent intracerebral calcification and diffuse brain disease, typically resulting in severe neurodisability.[1] There are currently no approved treatments, and there is a major unmet need to identify critical pathophysiological steps that inform treatment strategies. AGS is a monogenic disease, most often inherited as an autosomal recessive trait, with nine causative genes identified to date.[1,2] Several disease-associated mutations are linked to pathways of antiviral intracellular nucleic acid sensing and processing, and dysfunction leads to activation of the type I IFN (e.g., IFN-α) response. Notably, elevated IFN-α is a core immunological phenotype of AGS, observed across all disease-associated genotypes. Although AGS is a rare disease, its paradigmatic nature provides insight into a broad range of neurological and inflammatory diseases with an activated IFN response.

How the brain responds in a pathological manner to chronic elevation of IFN-α in AGS is unknown. A leading hypothesis is that IFN-α itself acts as a neurotoxin. However, the primary source of neurotoxic IFN-α has not been definitively identified or modeled.[3] Establishing its tissue origin, alongside which cells are particularly vulnerable to IFN-α, is crucial to our understanding of how the disease develops. Type I IFNs can lead to widespread activation of IFN response genes in almost all cell types via type I IFN receptor (IFNAR) signaling. Yet the extent and pathological cellular consequences of these responses differ and may also be influenced by the origins of the IFN-α. Thus, it is particularly important to establish whether a specific cell type within the brain is critically vulnerable to chronic activation of the type I IFN response since targeting cytokine-receptor

 © 2024 The Authors. Published by Elsevier Inc.
This is an open access article under the CC BY license (http://creativecommons.org/licenses/by/4.0/).

CellPress
OPEN ACCESS

interactions on that cell type might represent an important therapeutic strategy.

Addressing these questions requires unambiguous identification of the source and targets of neurotoxic IFN-α in AGS. However, studying downstream mechanisms of cytokine neurotoxicity in AGS has been particularly challenging for two reasons. Firstly, IFN-α proteins are low-abundance cytokines and difficult to detect in human samples.[4] The advent of ultrasensitive single-molecule ELISA (Simoa) technologies, combined with very high-affinity IFN-α antibodies derived from individuals with mutations in the thymic autoimmune regulator gene (*AIRE*) causing autoimmune polyendocrinopathy candidiasis-ectodermal dystrophy (APECED), offers an opportunity for direct quantification[4] and has reduced reliance on approaches that are based on indirect detection.[5] Secondly, it has been difficult to dissect the mechanisms of IFN-α neurotoxicity in mouse models of AGS. This is because most AGS mouse models based on patient mutations, such as *TREX1* and *RNASEH2B*, do not recapitulate neurological aspects of the disease.[6,7]

The aim of this paper is to identify the origins and cellular targets of neurotoxic IFN-α in AGS. We used Simoa to detect IFN-α in the serum and cerebral spinal fluid (CSF) across a spectrum of non-interferonopathic and interferonopathic diseases and showed that the central nervous system (CNS) is the primary source of neurotoxic IFN-α in AGS. We then replicated this focal IFN-α toxicity using a mouse model of brain-targeted *Ifna1* overexpression to investigate the downstream consequences of tissue-specific cytokine overproduction. Using this approach, we identified the microvasculature as a primary target of neurotoxic IFN-α and endothelial IFN-α signaling as a crucial initiator of diffuse brain disease in AGS. The cerebral microvasculature is therefore a critical mediator of IFN-α neurotoxicity in AGS and an accessible target for therapeutic intervention.

RESULTS

The CNS is the primary source of elevated IFN-α in AGS

Studying the distribution and dynamics of IFN-α in human disease has been challenging because these proteins typically exert biological effects at very low concentrations, below the level of detection of traditional ELISA technologies.[4] Recently, advances in Simoa technology, using super-affinity pan-IFN-α antibodies derived from individuals with *AIRE*-deficiency, have facilitated ultrasensitive quantification of IFN-α concentrations,[4] allowing for the accurate comparison of IFN-α concentrations in different human fluid compartments such as blood and CSF.

To determine the primary site of abnormal constitutive production of IFN-α in individuals with AGS, we first quantified IFN-α concentrations using Simoa from paired blood-CSF samples in control individuals (Figure 1A). This included healthy controls (19 blood-CSF pairs) and neuroinflammatory disease controls (individuals with multiple sclerosis, 32 blood-CSF pairs). In both control groups, blood and CSF IFN-α concentrations were low, with median serum and CSF IFN-α concentrations under 2 fg/mL (Figures 1B and 1C; Table S1). We next analyzed IFN-α concentrations from blood-CSF pairs from individuals with AGS (29 blood-CSF pairs, with clinical details and genotypes from Lodi et al.[8]). CSF IFN-α concentrations in AGS were significantly higher than paired blood ($p < 0.005$, Figure 1D), with median CSF IFN-α concentrations ∼300 times higher than healthy control CSF (552 fg/mL versus 1.74 fg/mL, respectively, Figures 1B–1D; Table S1).

We then asked if this intracerebral IFN-α elevation was specifically observed in AGS. We compared paired blood-CSF results from individuals with AGS to paired blood-CSF results from a cohort of individuals with systemic lupus erythematosus (SLE). SLE is a multiorgan autoimmune disease associated with prominent IFN-α activation (53 blood-CSF pairs, including samples combined from Lodi et al.[8] and Varley et al.[9]). We found that blood IFN-α concentrations were similarly elevated in both AGS and SLE individuals (median 154 and 195 fg/mL, respectively, $p = 0.9$, two-tailed Wilcoxon test; Figure 1B; Table S1). However, in contrast to AGS, CSF IFN-α concentrations were significantly lower than paired blood samples from individuals with SLE ($p < 0.0001$, two-tailed Wilcoxon test; Figures 1B and 1D). Taken together, these observations are consistent with a primary intracerebral origin of neurotoxic IFN-α in AGS.

Elevated intracerebral IFN-α mediates neurotoxicity in AGS

To test the hypothesis that brain-derived IFN-α is neurotoxic in AGS, we analyzed the neuropathological findings of mice overexpressing *Ifna1* in the CNS and compared these to the brain pathology observed in AGS. Both neuropathological and human-induced pluripotent stem cell studies of individuals with AGS have identified astrocytes as a potential cellular source of IFN-α in AGS,[11–13] which is modeled in transgenic mice that express *Ifna1* under the control of an astrocyte-specific promotor[14,15] (*Gfap-Ifna1*, referred to as GIFN mice throughout). Comparable to AGS, IFN-α concentrations were substantially higher in CSF than in plasma of GIFN mice (Figures S1A and S1B). GIFN mice specifically express *Ifna1* in the brain and universally develop severe brain disease in early life, including intracerebral calcification, confirmed by micro-computed tomography (CT) and alizarin red staining, which is also a characteristic neuroradiological feature of AGS (Figures 1E–1G). Additional neuropathological features of AGS, such as microvascular abnormalities, glial activation, and neuronal loss, were also recapitulated in GIFN mice (Figure 1G). We therefore reasoned that GIFN mice provide a disease-relevant system in which downstream cellular vulnerabilities to neurotoxic IFN-α in AGS could be explored.

Endothelial cells are the most responsive cell type to IFN-α in the brain

To identify IFN-responsive cells in the brain, we performed single-cell RNA sequencing (scRNA-seq, 10X Genomics) on cells isolated from the forebrains of GIFN and non-transgenic wild-type (WT) littermate control mice at 4 weeks, prior to the onset of symptomatic neurological disease (Figures 2A, S1C, and S1D). We observed widespread activation of IFN response genes, including all components of the "IFN signature" used in clinical practice as an AGS biomarker (Figure S1E). Quantification of *Ifnar1* and *Ifnar2* transcripts, encoding the subunits of the IFNAR and downstream signaling transducers of the canonical type I IFN signaling pathway, showed these components to be most highly expressed in cells of the cerebral

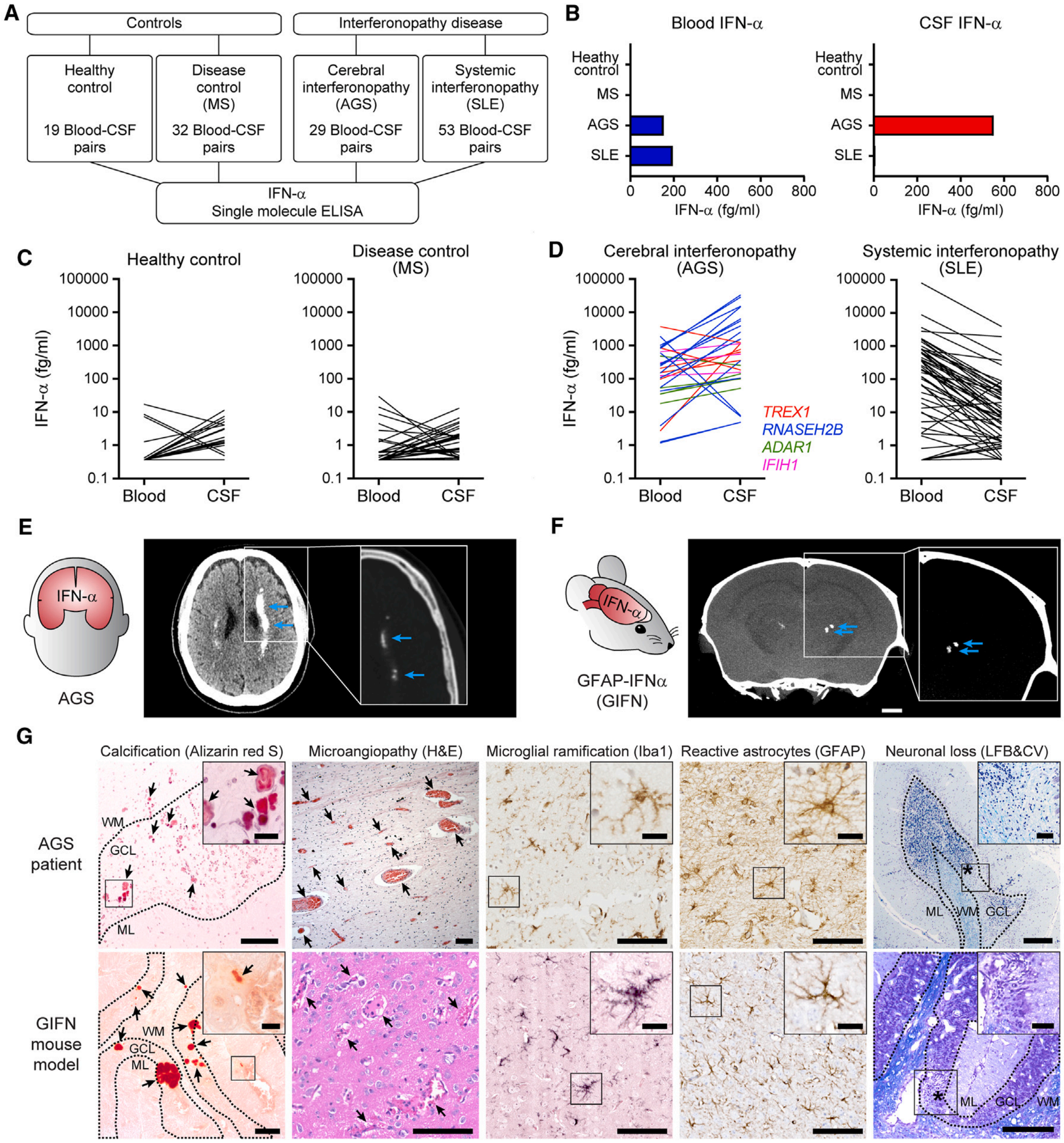

Figure 1. Neurotoxic interferon-alpha production in AGS is derived from an intracerebral source

(A) Overview of the systematic analysis of 133 paired serum-CSF IFN-α concentrations measured by Simoa in 51 control pairs (healthy controls and multiple sclerosis [MS], a non-interferonopathic disease) and 82 interferonopathic disease pairs (AGS and SLE, including cases from Lodi et al.[8] and Varley et al.[9]).

(B–D) Median (B) and individual concentrations (C and D) of IFN-α from blood and CSF (each line represents paired data) were determined by Simoa. (D) CSF concentrations are significantly higher than serum in individuals with AGS ($p = 0.005$, two-tailed paired Wilcoxon test). Serum IFN-α concentrations are significantly higher in serum than CSF in individuals with SLE ($p < 0.0001$, two-tailed paired Wilcoxon test). Data from 133 blood-CSF pairs.

(E) Computed tomography (CT) brain image from an individual with AGS showing bilateral calcification, a feature which is typically seen in AGS brain imaging[10] (blue arrows; inset is of a bone window, with calcification shown at the same signal as skull bone).

(F) Micro-CT of the head of a GIFN mouse infused with contrast, revealing bilateral intracerebral calcification (blue arrows; z-projection of 0.5 mm; scale bars, 1 mm). Inset is of a bone window. Representative image from $n = 2$ mice at 24 weeks of age from two independent experiments.

(G) Calcification (Alizarin red S stain, arrows point at calcium deposits; scale bars, 100 μm; scale bars inset, 20 μm), microangiopathy with enlarged capillaries (H&E stain, arrows point at enlarged blood vessels; scale bars, 100 μm), activation and hyper-ramification of microglia (Iba1 immunohistochemistry; scale bars, 100 μm; scale bars inset, 20 μm), reactive astrocytosis (glial fibrillary acidic protein [GFAP] immunohistochemistry; scale bars, 100 μm; scale bars inset, 20 μm) and neurodegeneration (Luxol fast blue and cresyl violet [LFB&CV] stain; asterisk: near total loss of granule cell neurons; scale bars, 250 μm; scale bars inset, 50 μm) within the brain parenchyma of a individual with AGS due to TREX1 mutation (c.598G>A p.Asp200Asn het) and in the brain of GIFN mice (representative from $n = 5$ mice at 16–24 weeks of age). GCL, granule cell layer; WM, white matter; ML, molecular layer. Representative images are from two independent experiments.

See also Tables S1 and S4A.

CellPress
OPEN ACCESS

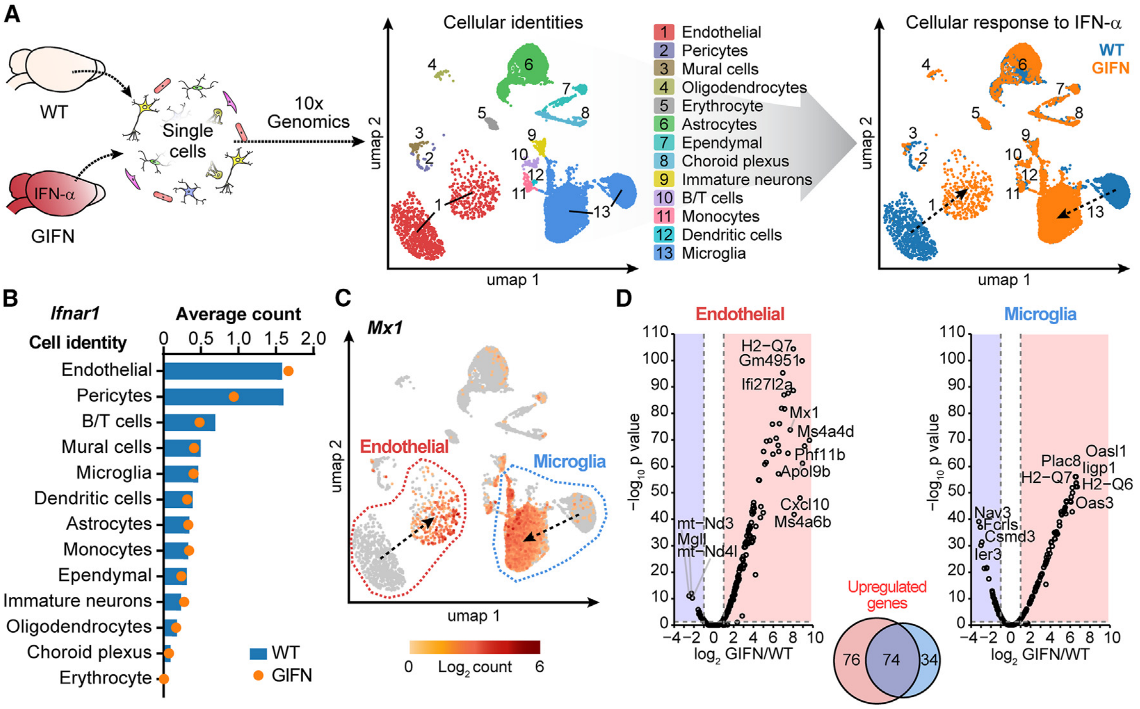

Figure 2. Endothelial cells are intracerebral targets of IFN-α

(A) Single-cell RNA sequencing was done on isolated cells from pooled forebrains of WT and GIFN mice. Cells were clustered and assigned an identity, which allowed for transcriptomic comparison between the two genotypes. Dashed arrows indicate shift in endothelial cells and microglia between WT and GIFN mice (genotype-separated UMAP plots shown in Figure S1C).

(B) Average counts of *Ifnar1* in various cell types and between genotypes.

(C) UMAP plot with counts of *Mx1* shown. Endothelial cells and microglia clusters are outlined.

(D) Volcano plot of genes from endothelial cells and microglia. Dotted lines indicate threshold for significance (adj. $p \leq 0.05$) and fold change (|fold change| ≥ 2). Venn diagram of upregulated genes.

(A–D) Data from one experiment.

See also Figure S1 and Table S2A.

microvasculature, in particular, endothelial cells, of both WT and GIFN mice compared with the other cell types (Figures 2B and S1F). Additionally, pericytes, vascular mural cells, microglia, and lymphocytes were cell types that ranked among the highest average counts of transcripts for type I IFN signaling components. Analysis of uniform manifold approximation and projection (UMAP) plots showed that cerebral endothelial cells and microglia undergo substantial transcriptomic alterations and change in cellular identity in GIFN mice (Figures 2A and 2C). Hence, both endothelial cells and microglia showed a broad response of IFN-stimulated gene upregulation with a common core and cell-type-specific response, with the broadest and most conspicuous response being seen in endothelial cells (Figures 2C, 2D, and S1G; Table S2). The pronounced response of blood vessel-associated cells to chronic IFN-α suggests that the microvasculature, in particular endothelial cells, might act as a primary mediator of IFN-α neurotoxicity.

Chronic intracerebral activation of endothelial IFNAR causes a distinctive cerebral microangiopathy

To further clarify the consequences of constitutively active IFNAR signaling in cerebral endothelial cells *in vivo*, we extended our scRNA-seq analyses by sequencing CD31-enriched cells from the brains of WT and GIFN mice aged 4 weeks (Figures 3A and S3A). Focusing on endothelial cells, we identified significant upregulation of genes and pathways implicated in endothelial cell function, including cellular adhesion molecules (*Vcam1*) and IFN-stimulated genes involved in chemotaxis (*Cxcl10*) and antigen presentation (*H2-K1*) (Figures 3A, 3B, and S3B; Table S2).

Importantly, the changes in gene expression at 4 weeks preceded the development of a widespread microangiopathic disease that is present in GIFN mice at 16 weeks. The microangiopathy at 16 weeks of age was characterized by upregulation of cell adhesion molecules and major histocompatibility complex (MHC) class I, lymphocyte infiltration (Figure 3C), and development of small vessel aneurysms (Figure 3D). Microangiopathic disease in GIFN mice also involved breakdown of the blood-brain barrier (BBB) with leakage of Evan's blue and fluorescein into the brain parenchyma (Figure 3E). Together, these findings demonstrate that the transcriptional changes induced by IFN-α in the CNS temporally precede the development of a prominent cerebral microvascular disease phenotype.

We next asked whether this IFN-driven cerebral microvascular disease displayed distinctive and conserved neuropathological features between mice and humans. Comparison of brain disease and neuropathology between GIFN mice and individuals

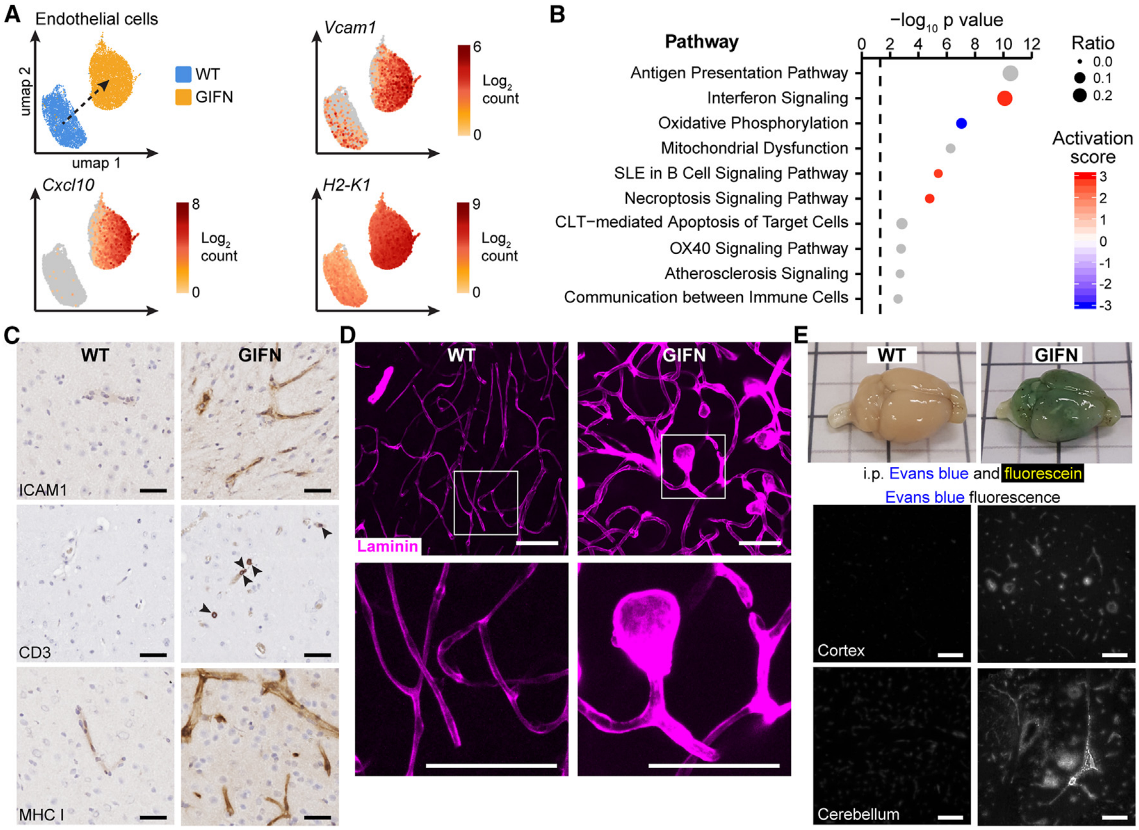

Figure 3. Type I interferon signaling in endothelial cells causes a distinctive cerebral microangiopathy

(A) Transcript counts of endothelial activation markers (*Vcam1*), chemokines (*Cxcl10*), and MHC class I (*H2-K1*) in endothelial cells between WT and GIFN mice (scRNA-seq on CD31-enriched cells).

(B) Pathway analyses of significantly regulated genes and associated predicted activity scores are shown. Dashed line: significance threshold.

(C) Immunohistochemical staining for the endothelial activation marker ICAM1, CD3-positive T cells (arrowheads indicate T cells), and MHC class I ($n = 4$ per genotype at 16 weeks of age; scale bars, 20 μm). Representative images from two independent experiments.

(D) Laminin staining of passively cleared cerebral cortices. Abnormal microvasculature in GIFN mice with enlarged capillaries and microaneurysms (scale bars, 100 μm; $n = 4$ per genotype at 16 weeks of age). Representative images from two independent experiments.

(E) Evans blue and fluorescein injected into WT and GIFN mice. Perfused brain photographed before sectioning ($n = 4$ per genotype at 16 weeks of age; black lines are 1 cm apart and bisected by gray lines) and imaging for Evans blue fluorescence of the cortex and cerebellum (scale bars, 100 μm). Representative image from two independent experiments.

(A and B) Data from one experiment.

See also Figure S2 and Table S2B.

with AGS revealed shared distinctive features, including perivascular T cell infiltration, vessel-associated calcification, vascular caliber variations, and aneurysm formation (Figure 4). Thus, microangiopathy is not just a feature in GIFN mice but also prominently present in brains from individuals with AGS, consistent with a role in the disease pathogenesis.

Cerebral microangiopathy in AGS is primarily driven by IFN-α of CNS origin rather than peripheral blood origin

Taken together, these data suggest that cerebral microangiopathy in AGS is largely driven by very high concentrations of IFN-α produced within the CNS. Consistent with this, an analysis of the largest available AGS cohort[5] demonstrated a clear inverse correlation between IFN-α activity in the CSF and time to onset of the disease, with a dose-dependent effect (Figure 5A). This association with time to onset was observed for CSF IFN-α and not serum IFN-α (Table S3).

However, although our single-molecule ELISA analyses of human interferonopathic disease in Figure 1 showed a primary CNS source of elevated IFN-α in individuals with AGS, IFN-α concentrations were also raised in circulating blood as well. This elevation was similar to that observed in "peripheral interferonopathic" diseases such as SLE (Figure 1B). Given that circulating type I IFN has been implicated in endothelial-mediated brain changes,[16] we asked whether peripheral blood IFN-α might contribute substantially to the microangiopathy observed in cerebral interferonopathy.

To address this question, we examined the association between circulating blood IFN-α concentrations and microvascular disease in individuals with SLE, who we have shown to exhibit very high peripheral blood IFN-α concentrations yet substantially lower CSF IFN-α concentrations (Figure 1D). We quantified cerebral microvascular disease using brain magnetic resonance imaging (MRI) in 47 individuals with SLE and

CellPress
OPEN ACCESS

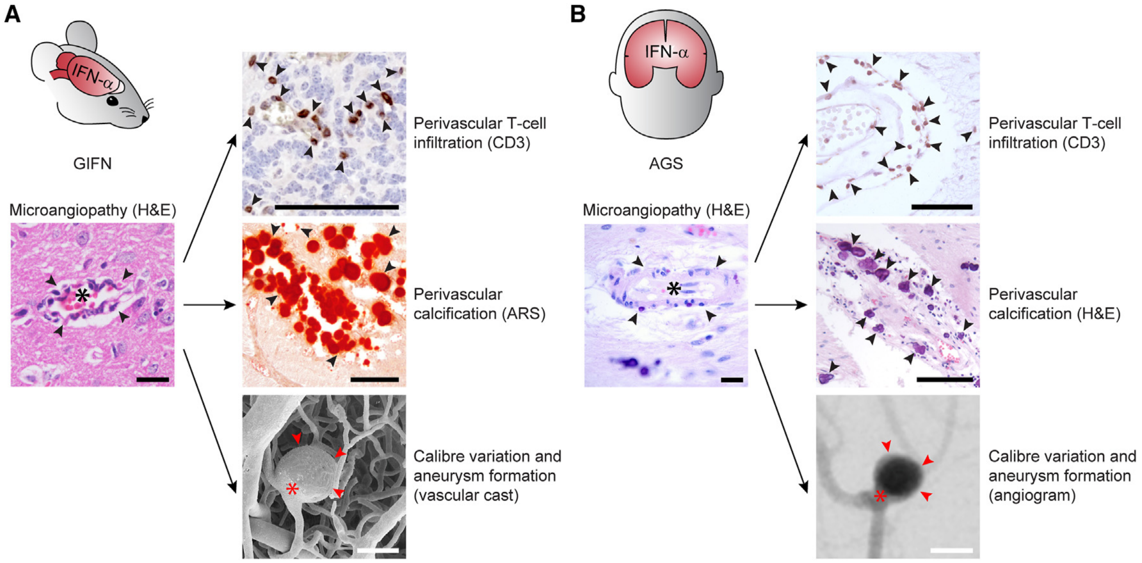

Figure 4. Distinctive features of interferon cerebral microangiopathy are observed in individuals with AGS

(A and B) Activated and dysmorphic capillaries in microangiopathy (vessel lumen indicated by asterisks and capillary indicated by arrowheads; scale bars, 20 μm) are associated with perivascular T cell infiltrates (indicated by arrowheads; scale bars, 50 μm), perivascular calcification (indicated by arrowheads; scale bars, 100 μm), changes to capillary caliber, and aneurysm formation (aneurysm neck indicated by asterisks and aneurysm outpouching indicated by arrowheads; vascular cast: scale bars, 50 μm; angiogram: scale bars, 5 mm). Representative immunohistochemistry images from two independent studies and one study for the cast and angiogram.

measured blood IFN-α concentrations at the time of imaging (Figures 5B and 5C). Cerebral small vessel disease (SVD) in each subject was quantified using validated SVD burden and Fazekas scores.[17] We found that peripheral blood IFN-α concentrations did not correlate with the development of microvascular disease in people with SLE (Figures 5D and 5E). Noting that almost all individuals with AGS have blood concentrations of IFN-α over 10 fg/mL, we further stratified the SLE cohort into individuals who have very high "AGS concentrations" of IFN-α in the peripheral blood (>10 fg/mL) and those who have lower concentrations. There was no significant difference in SVD burden or Fazekas score between these two groups (Figures 5D and 5E). As such, the concentration of IFN-α in the peripheral blood observed in individuals with AGS does not confer a substantially increased risk of cerebral microvascular disease. Taken together, these results suggest that cerebral microangiopathy in AGS is primarily driven by IFN-α of CNS origin rather than peripheral blood origin.

Ablation of endothelial IFNAR rescues cerebral microangiopathic disease

To test whether chronic activation of IFNAR signaling within endothelial cells mediates microvascular disease, we generated GIFN mice with endothelial cell deletion of *Ifnar1* (Figure 6A). These triple transgenic mice (GIFN$^{+/-}$ x *Ifnar1*$^{fl/fl}$ x Tek-Cre$^{+/-}$, termed "GIT" mice) exhibited near-complete rescue of cerebral vascular disease, with decreased ICAM1 and MHC class I production and reduced leukocyte infiltration, compared with GIFN$^{+/-}$ x *Ifnar1*$^{fl/fl}$ x Tek-Cre$^{-/-}$ mice (termed "GIFN-FL" mice) (Figure 6A). Although these findings suggest a primary role for endothelial IFNAR1 signaling in T cell recruitment to the CNS parenchyma in GIFN-FL mice, it is conceivable that astrocytes and microglia may contribute to this process. Importantly, the cerebral microvasculature of GIT mice appeared similar to littermates, which did not produce excess IFN-α within the brain (GIFN$^{-/-}$ x *Ifnar1*$^{fl/fl}$ x Tek-Cre$^{-/-}$, termed "WT-FL" mice) and without overt pathological changes (Figure 6B; Video S1). By contrast, pronounced morphological abnormalities were seen in brain capillaries of GIFN-FL mice, including microvascular dilation and microaneurysm formation (Figure 6B; Video S1). Quantification of the cerebral microvasculature revealed dilation of small vessels and string vessels (remnants of capillaries) in GIFN-FL mice, while capillaries in GIT mice mirrored those of WT-FL mice (Figures 6C–6G).

We next investigated the functional consequences of the microangiopathy in GIFN mice by assessing BBB integrity. Although BBB integrity was compromised in GIFN-FL mice, deletion of the IFNAR gene *Ifnar1* from endothelial cells in GIT mice was sufficient to restore its function (Figures 6, S3A, and S3B). Furthermore, expression of endothelial cell activation markers *Icam1*, *Eng*, and *Sele* was significantly reduced in GIT mice compared with GIFN-FL mice (Figure S3C). We therefore conclude that loss of endothelial type I IFN signaling prevents microvascular disease in the brains of GIFN mice.

GIT mice show rescued extravascular IFN-α neurotoxicity

We next examined the development of the diffuse extravascular brain disease between GIFN and GIT mice. There were strikingly reduced neuropathological changes in GIT mice compared with GIFN-FL mice at 16 weeks of age (Figures 7A and S3–S5). Notably, there was the absence of overt tissue destruction, neuronal loss, and calcification in brains of GIT mice, as well as a partial reversal of gliosis. Functionally, GIT

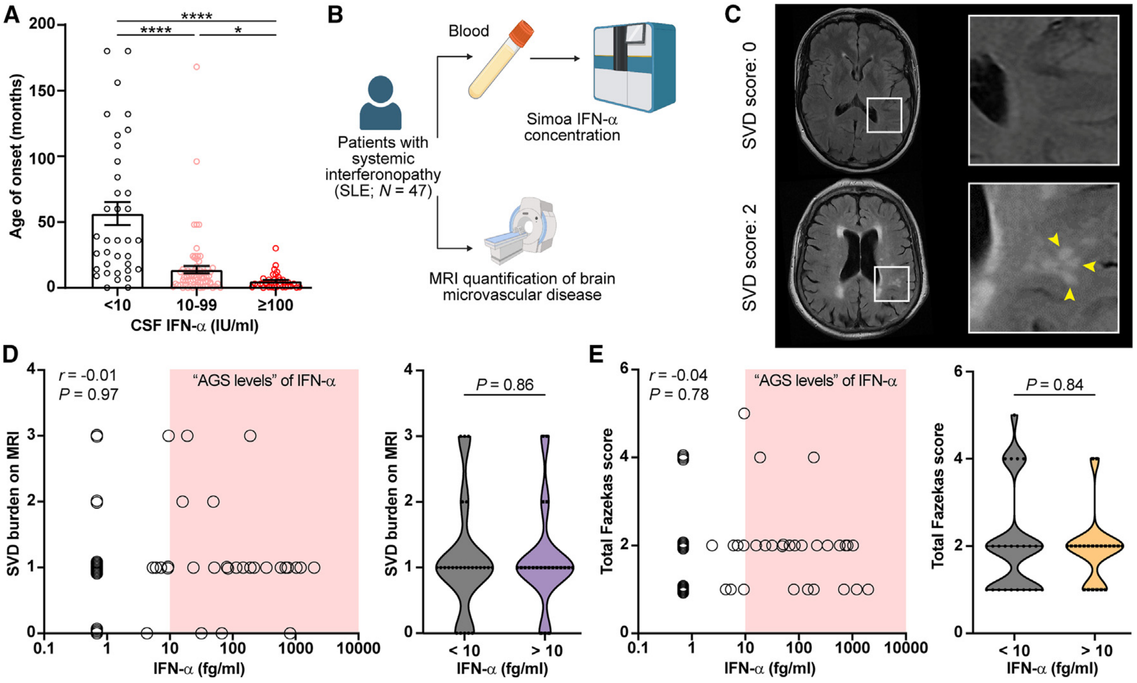

Figure 5. Cerebral microangiopathy in AGS is primarily driven by IFN-α of CNS origin rather than peripheral blood origin

(A) Analysis of CSF IFN-α activity and disease severity indicated by time to onset of disease (specifically, time of diagnostic lumbar puncture) in a large cohort of individuals with AGS.[5] Stratified data with Kruskal-Wallis test. *$p < 0.05$ and ****$p < 0.0001$.

(B) Overview of MRI study. Forty-seven individuals with SLE underwent paired IFN-α Simoa and MRI of the brain to quantify cerebral small vessel disease.

(C) Representative MRI brain images of two individuals with SLE illustrating appearances of small vessel disease. Top image: no SVD present (SVD score = 0) and lower image (SVD score = 2): hyperintensities are seen in the white matter, reflecting small vessel disease (yellow arrowheads). Representative images from 47 individuals with SLE.

(D and E) Correlation plots between serum IFN-α concentrations and SVD burden score or Fazekas score (n = 47 individuals with SLE). Red box highlights concentration typically observed in AGS (>10 fg/mL; n = 22). Correlation analyzed with Spearman's rank correlation and stratified data with two-tailed Mann-Whitney U test.

mice also displayed improved motor coordination compared with GIFN-FL mice (Figure 7B, S5G, and S5H; Video S2). Importantly, the disease rescue in GIT mice occurred despite evidence of widespread IFN-α expression and intracerebral activation of the type I IFN response (Figures 7C, 7D, S5I, and S5J). This cumulative rescue of the cellular phenotypes and pathology led to GIT mice surviving longer with near-normal neurological phenotype (Figures 7E and S6) and no demonstrable neuropathological abnormalities, even in mice aged 8 months (Figure S6), markedly contrasting with the deterioration seen in GIFN-FL mice.

Tek-Cre mice are widely used to perform endothelial-specific deletion experiments, and *Tek* expression is largely specific for cerebral endothelial cells, as confirmed by our scRNA-seq data (Figures S7A and S7B) and immunohistochemistry (Figure S7C). However, we identified recombination within microglia and hematopoietic cells in *Ifnar1*$^{fl/fl}$ x Tek-Cre$^{+/-}$ mice (Figures S7D and S7E), which may be related to transient *Tek* expression in early hematopoietic cell development.[18] Our previous work has shown that microglia are not major mediators of IFN-α neurotoxicity.[19] We sought further confirmation of this by generating a second triple transgenic mouse line with microglia-specific deletion of *Ifnar1* (GIFN$^{+/-}$ x *Ifnar1*$^{fl/fl}$ x *Cx3cr1-Cre*$^{+/-}$, termed "GIC" mice). In contrast to GIT mice, microglia-specific inactivation of *Ifnar1* had no significant improvement on motor disease and minimal impact on the survival of GIC mice compared with GIFN-FL mice (Figures S7F and S7G). Further, microglia in young GIFN mice did not have enrichment of expressed genes associated with effector pathways like phagocytosis[20] (Figure S7H). To assess further IFN-α effects on phagocytosis, we determined this in IFN-α-stimulated primary microglia. Compared with unstimulated cells, 72 h stimulation with IFN-α did not alter uptake of particles in microglia (Figure S7I). Therefore, consistent with previous published findings, these analyses suggest a minor role only for microglia in mediating IFN-α neurotoxicity.

Taken together, these human and murine studies identified the microvasculature and endothelial cells as the major target of centrally produced neurotoxic IFN-α. Inhibition of IFNAR signaling in endothelial cells prevented development of diffuse brain disease and neurodegeneration, resulting in a significant increase of survival compared with GIFN-FL mice. Together, these results demonstrate the cellular mechanism of IFN-α-induced neurotoxicity is primarily mediated by endothelial cells, where stimulation of IFNAR1 signaling results in direct activation of multiple pathological pathways, causing a complex yet distinctive microvascular phenotype. The resulting loss of BBB integrity, infiltration of immune cells, and development of

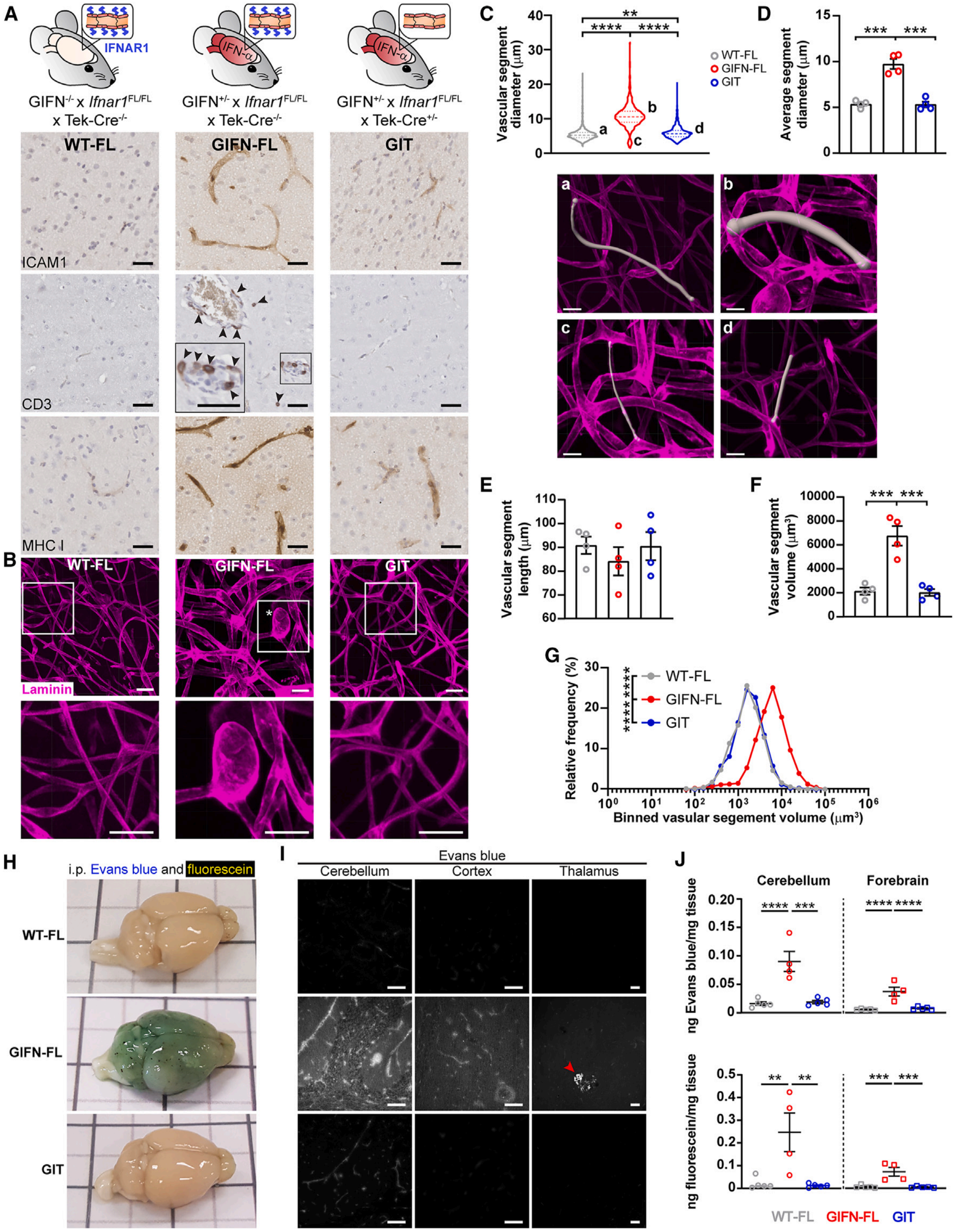

Figure 6. Endothelial deletion of IFNAR1 reverses cerebral microangiopathy in GIFN mice

(A) Immunohistochemistry for endothelial activation marker ICAM1, CD3-positive T cells (arrowheads), and MHC class I immunostaining ($n = 4$ per genotype at 16 weeks of age; scale bars, 30 μm). Representative images from two independent experiments.

(B) Laminin-stained passively cleared cortices. Abnormal microvasculature in GIFN-FL mice with enlarged capillaries and microaneurysms ($n = 4$ per genotype at 16 weeks of age; scale bars, 30 μm; asterisks: aneurysm). Representative images from two independent experiments.

(C) Violin plot (median and interquartile range indicated) of cortical vascular segments (portions between vascular branches). (A–D) Representative images of traced vascular segments at indicated segment diameters. Scale bars, 20 μm.

(D) Average vascular segment diameter.

(legend continued on next page)

aneurysms and calcification lead to increased glial reactivity, neuronal loss, and ultimately death.

DISCUSSION

In this paper, we analyzed blood-CSF pairs from individuals across healthy and interferonopathic disease states to demonstrate a primary CNS origin of neurotoxic IFN-α in AGS. We showed, using a study of blood IFN-α concentrations and brain MRI, that central IFN-α, and not peripheral IFN-α, is the primary determinant of cerebral disease. We then used different transgenic mouse models to demonstrate the neurotoxicity of elevated brain-derived IFN-α and identify cerebral endothelial cells and their IFNAR signaling as a critical bottleneck for mediating this toxicity. As such, we have identified important up-stream points of potential early therapeutic intervention, within the complex neuropathological cascade that occurs in AGS.

Although elegant mouse studies have focused on how circulating type I IFN contributes to brain dysfunction,[16] our Simoa data showed that in AGS, the primary source of the neurotoxic IFN-α was in the CNS itself, reflecting a profoundly disordered autoinflammatory state with prominent brain-specific activation of the type I IFN response. We also showed that microangiopathy and brain disease in AGS were primarily associated with IFN-α of CNS origin and independent of peripheral IFN-α concentrations. This key tissue-specific feature of a CNS-dominant disease is not replicated by genetic models of AGS.[7,21] For example, most mouse models based on enzymatic loss of function of *TREX1* and *RNaseH2* have near-normal brains with minimal upregulation of type I IFN-stimulated genes.[7,21] By contrast, GIFN mice recapitulate the core clinical and pathological features of individuals with AGS, and transgene expression is specific to the CNS,[14] producing widespread upregulation of IFN-stimulated genes, including the specific genes that form part of the "IFN score" used in clinical practice for individuals with AGS. Although there may be low GFAP expression in non-CNS cells, such as Schwann cells, peripherally circulating IFN-α was only a fraction of the IFN-α concentration in the CNS in the GIFN model, similar to what we observed in individuals with AGS. Thus, although our findings clearly demonstrate that cerebrally produced IFN-α mediates neurotoxicity in individuals with AGS and the GIFN mouse model, a minor contribution of peripherally produced IFN-α to brain disease cannot be excluded. However, our paired Simoa-MRI analyses suggested that IFN-α, even when produced in the blood at concentrations seen in AGS, does not contribute substantially to the development of cerebral microvascular disease. As such, we believe the GIFN mouse represents an important and suitable mouse model to study intracerebral events downstream of IFN-α production. Although we recognize the limitations of studying a model that is not based on an established genetic cause of AGS, intracerebral IFN-α elevation was observed across genotypes, as demonstrated by our Simoa data. Therefore, the findings are likely more broadly applicable to AGS and are agnostic to genetic mutation, providing important complementary insights to models based on underlying genetic causes.

Our data identified damage to the cerebral microvasculature as a critical first step in the IFN-α neurotoxicity observed in AGS and pinpointed IFNAR signaling within endothelial cells as a key site for potential therapeutic intervention. Inappropriate or chronic production of IFN-α within the brain caused upregulation of many endothelial-specific IFN-stimulated genes, including cellular adhesion molecules, chemokines, and genes involved in antigen presentation, leading to a distinctive cerebral microangiopathy characterized by endothelial activation, capillary malformation, and lymphocyte infiltration, which was accompanied by major functional defects such as BBB breakdown. Importantly, we showed that diffuse aspects of interferonopathic brain disease such as calcification and neuronal loss arose as a downstream consequence of this cytokine-induced microangiopathy. Elegant studies of viral sickness behavior and delirium have identified brain endothelial cells as natural gatekeepers for virus-induced cognitive dysfunction following peripheral infection, although brain disease in this setting is typically mild.[16] Similarly, peripherally administered recombinant type I IFN therapies can rarely cause a renal thrombotic microangiopathy when used to treat conditions such as multiple sclerosis,[22] but have differing impacts on the brain compared with AGS and GIFN mice. Our study adds to this work by showing that endothelial cells play a critical gatekeeping role when neurotoxic IFN-α is produced from within the CNS rather than peripheral blood. Our Simoa analyses showed that the elevated IFN-α concentrations in individuals with AGS were largely CNS-derived, although significant elevations were also detected in the blood. This raises an important question as to whether such peripheral elevations of IFN-α could contribute to the observed cerebral microangiopathy. We have addressed this question by performing analyses of blood IFN-α concentrations and simultaneous brain imaging in individuals with a systemic interferonopathy (i.e., SLE), where our analyses showed serum IFN-α concentrations are an order of magnitude higher than in the CSF. Using this approach, we demonstrated that elevated systemic IFN-α is

(E) Average vascular length.
(F) Average vascular volume.
(G) Binned distribution of vascular segment volume.
(H) Mice were injected intraperitoneally (i.p.) with Evans blue, with overnight circulation, followed by fluorescein injection, and then perfused after 30 min. Evans blue and fluorescein permeate a leaky brain ($n = 5$ for WT-FL and GIT and 4 for GIFN-FL at 16 weeks of age; black lines are 1 cm apart and bisected by gray lines).
(I) Fluorescent images of brain sections for Evans blue revealing the spatial leakage in different brain regions. Red arrowhead: calcification; scale bars, 100 μm.
(J) Quantification of Evans blue and fluorescein extracted from the cerebellum and forebrain and detected by fluorescence spectroscopy. No comparisons were made between brain regions or between dyes. Quantification was one experiment from samples collected from more than three independent experiments.
(D–F and J) Each point is a mouse. Mean ± SEM. are shown, unless otherwise stated. Statistical comparisons were performed using one-way ANOVA with Tukey's post-test. **$p < 0.01$, ***$p < 0.001$, and ****$p < 0.0001$. (C–G) Pooled data from two independent experiments. (H and I) Representative images from over three independent experiments.
See also Figure S3 and Video S1.

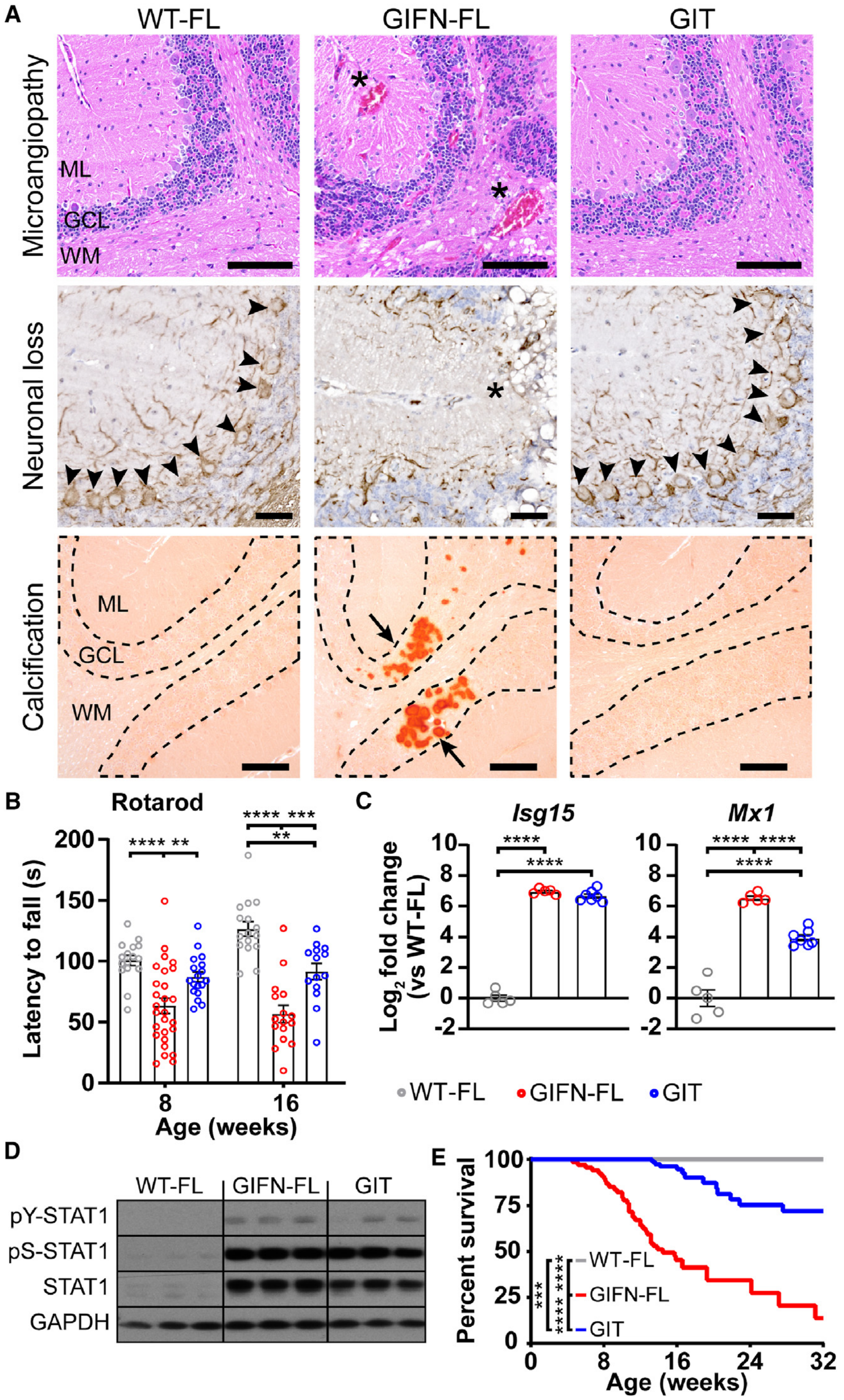

Figure 7. Endothelial deletion of IFNAR1 prevents the development of extravascular disease and prolongs survival of GIFN mice

(A) Top row: hematoxylin and eosin stain of the cerebellum with neuronal loss in the granule cell layer (GCL), vacuolation in the white matter (WM), and enlarged vessels/aneurysms (asterisks) observable in GIFN-FL mice (scale bars, 100 μm; ML, molecular layer). Middle row: neurofilament immunohistochemistry shows Purkinje neurons (arrowheads) and their absence in GIFN-FL mice (asterisk; scale bars, 30 μm). Bottom row: calcification in the GCL revealed with alizarin red S only in GIFN-FL mice (arrow: clustered calcified deposits in the GCL; scale bars, 100 μm; $n = 4$ per genotype at 16 weeks of age). Representative images from two independent experiments.

(B) Gross motor coordination tested by rotarod ($n = 15$ for WT-FL, 16 for GIFN-FL, and 18 for GIT; repeated measures linear mixed-effects models with Tukey's post-test; points represent individual animals; and error bars represent mean ± SEM). Data pooled from more than three independent experiments.

(C) Expression of type I interferon-stimulated genes in the cerebella detected by qPCR ($n = 5$ for WT-FL and GIFN-FL and 7 for GIT at 16 weeks of age; one-way ANOVA with Tukey's post-test). Points represent individual animals, and error bars represent mean ± SEM. Quantification was one experiment from sample collected from more than three independent experiments.

(D) Immunoblots for type I interferon signaling in the cerebella based on STAT1 activation ($n = 4$–5 for WT-FL and GIFN-FL and 7 for GIT at 16 weeks of age). Quantification was one experiment from samples collected from more than three independent experiments.

(E) Survival analysis of GIT mice and littermate controls (total $n = 70$ for WT-FL, 67 for GIFN-FL, and 86 for GIT mice). Significance determined by log-rank test with Benjamini-Hochberg post-test. Data pooled from more than three independent experiments.

*$p < 0.05$, **$p < 0.01$, ***$p < 0.001$, and ****$p < 0.0001$.
See also Figures S4–S6 and Video S2.

not associated with the development of cerebral microangiopathy, and those individuals with very high concentrations of serum IFN-α (i.e., blood concentrations typically observed in individuals with AGS, >10 fg/mL) did not have more SVD in the CNS than those with lower concentrations. We have also demonstrated that in AGS, it is the concentrations of cerebral IFN-α and not peripheral blood IFN-α that correlated most strongly with markers of disease severity, such as time to onset.[5]

Therefore, our converging lines of evidence from human and mouse studies showed that disabling and lethal cerebral microvascular disease is the consequence of CNS IFN-α overproduction. This scenario is seen in distinctive genetic disorders such as AGS, but also potentially in other clinical scenarios such as CNS viral infection. These results further highlight that the microvasculature could be targeted to prevent the devastating consequences of IFN-α neurotoxicity in these clinical settings. Taken together, these studies show that distinct clinical phenotypes can be caused by chronic type I IFN elevation, and the source of the IFN-α is a major determinant of the nature of these illnesses.

These results have potential implications for how AGS is classified. Little is known of the cellular dynamics of dysregulated

IFN signaling within the brain in AGS and other cerebral interferonopathies. Likely, this is due in part to the failure of genetic mouse models to recapitulate the intracerebral synthesis and neurotoxicity of type I IFN,[23] but also because neuropathological studies often show diffuse end-stage brain damage involving multiple cell types. Since a critically vulnerable IFN-responsive cell type has not yet been identified, AGS has been formally classified as a "leukodystrophy" or "astrocytopathy," suggesting oligodendrocytes or astrocytes play key roles in the degenerative cascade.[24] However, our data provide experimental evidence that endothelial cells are a primary target of IFN toxicity in AGS, in turn suggesting that AGS may be best considered a cerebral microangiopathy or vasculopathy, a clinical hypothesis first suggested last century.[25–27]

There is currently a substantial translational effort to develop therapeutic strategies for AGS,[28–30] and elucidation of the spatial dynamics of disordered IFN signaling within the brain will inform the development of optimal therapeutic strategies and clinical trial design.[28] Current treatment and ongoing clinical trials focus on the use of Jak inhibitors and reverse transcriptase inhibitors, which have broad effects including modulation of type I IFN signaling (e.g., ClinicalTrials.gov Identifier: NCT03921554 and NCT04731103). However, our findings collectively suggest that constitutively activated type I IFN signaling within endothelial cells may represent an important therapeutic target for AGS and one that is readily accessible to systemically delivered drugs. Thus, we propose that targeting IFN-driven microangiopathy prior to the onset of diffuse brain disease may represent an important neuroprotective strategy in AGS and cerebral interferonopathies.

Limitations of the study

To clarify the contribution of the cerebral vasculature to disease in AGS, we have used an established model of endothelial cell genetic deletion using a Tek-Cre approach in our GIFN mice. However, all endothelial-specific Cre drivers have some limitations in interpretation, in particular Cre-mediated deletion in other cell types.[31,32] Although *Tek* was highly expressed in endothelial cells, we also observed *Tek* expression in pericytes, and it is therefore possible that IFN-driven pericyte dysfunction contributed to IFN microangiopathy. This may be particularly relevant given that one distinct feature of IFN-driven cerebral microangiopathy was the development of microaneurysms. These focal outpouchings of capillaries can arise as a consequence of pericyte dysfunction or ablation, possibly due to focal loss of mural integrity.[33] Although *Tek* expression was not observed in microglial cells, some recombination of *Ifnar1* occurred in microglia, which may reflect a hematopoietic origin of these cells, where excision is known to occur. However, several lines of evidence pointed to a minor or even protective role for microglia in mediation of IFN neurotoxicity. Firstly, recently published work demonstrates that microglia depletion aggravates disease in GIFN mice[19]; secondly, transcriptomic and *in vitro* analyses suggest that IFN-α-stimulated microglia unlikely participate in effector pathways like phagocytosis[20]; and thirdly, our microglial-specific knockout experiment here showed no significant impact on neurological disease or survival in GIFN mice.

Although further study of the minor or even protective contribution of pericytes and microglia to interferonopathic brain disease will also be of merit, converging evidence from humans and mouse models supports a primary role for the microvasculature in mediating IFN-α-driven neurotoxicity.

STAR★METHODS

Detailed methods are provided in the online version of this paper and include the following:

- KEY RESOURCES TABLE
- RESOURCE AVAILABILITY
  - Lead contact
  - Materials availability
  - Data and code availability
- EXPERIMENTAL MODELS AND STUDY PARTICIPANT DETAILS
  - Human samples
  - Mice
- METHOD DETAILS
  - Primary Cultures
  - Simoa study
  - Time to onset analyses
  - SLE brain MRI study
  - MRI image review and visual rating
  - Total SVD score
  - Micro-CT
  - Single cell isolation and library preparation
  - Single cell RNA sequencing analysis and bioinformatics
  - Histopathology
  - Passive tissue clearing
  - Single cell morphometric quantification
  - Quantitative real-time PCR
  - Blood-brain barrier leakage
  - Motor function quantification
  - Protein isolation and immunoblot
  - IFN-α ELISA
  - Cell type validation of recombination
  - Phagocytosis assay of primary murine microglia
- QUANTIFICATION AND STATISTICAL ANALYSIS

SUPPLEMENTAL INFORMATION

Supplemental information can be found online at https://doi.org/10.1016/j.immuni.2024.05.017.

ACKNOWLEDGMENTS

We thank Grant Foster, Holly Philip, and Ben Gartland of Ward6 Australia for assistance with the graphical abstract and Claire Thompson for editorial help with the manuscript. B.V. and E.H. were supported by an Australian Government Research Training Program Scholarship. We thank Dr Peter Crack (University of Melbourne) for kindly providing *Ifnar1*-floxed mice. We are grateful to Dr Thomas Krucker (Novartis) and Dr Eric Meyer (University of Zurich) for providing scanning electron microscopy image of Mercox microvascular cast in Figure 4. The authors also acknowledge technical support and facilities of The University of Sydney: Laboratory Animal Services, Histopathology Laboratory, The Bosch Institute and core research facilities, Sydney Cytometry, Sydney Imaging, and Sydney Informatic Hub. The schematic in Figure 5B was created using biorender.com. This work was supported by grants: National Health and Medical Research Council Australia Ideas Grant APP2001543 (M.J.H.), Wellcome Trust 215621_Z_19_Z and the Medical Research Foundation and Connect Immune (D.P.J.H.), European Research Council GA309449 and 786142-E-T1IFNs (Y.J.C.), UK Dementia Research Institute (award no. UKDRI – Edin002, DRIEdi17/18, and MRC MC_PC_17113), which receives its funding from DRI Ltd, funded by the UK Medical Research Council, Alzheimer's Society and Alzheimer's Research

UK (J.W.), and Stroke Association (SA PDF 18\100026), a Principal's Scholarship and Lupus UK Grant (S.W.).

AUTHOR CONTRIBUTIONS

B.V., I.L.C., D.P.J.H., and M.J.H. conceived the study. B.V., E.H., G.G.N., S.M., D.P.J.H., and M.J.H. designed the experiments. B.V., E.H., R.V., and L.L. conducted the mouse experiments. S.W. and J.W. conducted MRI studies on individuals. A.P. performed the histological analysis on the human brains. Y.J.C. ascertained human neuropathological material. B.V., E.H., and K.E. analyzed the data. B.V., D.P.J.H., and M.J.H. wrote the paper with input from all of the authors.

DECLARATION OF INTERESTS

I.L.C. and M.J.H. received funding from Ionis Pharmaceuticals for experiments in an unrelated study using the GIFN mice.

Received: February 22, 2022
Revised: January 10, 2024
Accepted: May 17, 2024
Published: June 14, 2024

REFERENCES

1. Crow, Y.J., Chase, D.S., Lowenstein Schmidt, J., Szynkiewicz, M., Forte, G.M.A., Gornall, H.L., Oojageer, A., Anderson, B., Pizzino, A., Helman, G., et al. (2015). Characterization of human disease phenotypes associated with mutations in TREX1, RNASEH2A, RNASEH2B, RNASEH2C, SAMHD1, ADAR, and IFIH1. Am. J. Med. Genet. A *167A*, 296–312. https://doi.org/10.1002/ajmg.a.36887.
2. Uggenti, C., Lepelley, A., Depp, M., Badrock, A.P., Rodero, M.P., El-Daher, M.T., Rice, G.I., Dhir, S., Wheeler, A.P., Dhir, A., et al. (2020). cGAS-mediated induction of type I interferon due to inborn errors of histone pre-mRNA processing. Nat. Genet. *52*, 1364–1372. https://doi.org/10.1038/s41588-020-00737-3.
3. Crow, Y.J., Shetty, J., and Livingston, J.H. (2020). Treatments in Aicardi-Goutières syndrome. Dev. Med. Child Neurol. *62*, 42–47. https://doi.org/10.1111/dmcn.14268.
4. Rodero, M.P., Decalf, J., Bondet, V., Hunt, D., Rice, G.I., Werneke, S., McGlasson, S.L., Alyanakian, M.A., Bader-Meunier, B., Barnerias, C., et al. (2017). Detection of interferon alpha protein reveals differential levels and cellular sources in disease. J. Exp. Med. *214*, 1547–1555. https://doi.org/10.1084/jem.20161451.
5. Rice, G.I., Forte, G.M.A., Szynkiewicz, M., Chase, D.S., Aeby, A., Abdel-Hamid, M.S., Ackroyd, S., Allcock, R., Bailey, K.M., Balottin, U., et al. (2013). Assessment of interferon-related biomarkers in Aicardi-Goutières syndrome associated with mutations in TREX1, RNASEH2A, RNASEH2B, RNASEH2C, SAMHD1, and ADAR: a case-control study. Lancet Neurol. *12*, 1159–1169. https://doi.org/10.1016/S1474-4422(13)70258-8.
6. Crow, Y.J., and Stetson, D.B. (2022). The type I interferonopathies: 10 years on. Nat. Rev. Immunol. *22*, 471–483. https://doi.org/10.1038/s41577-021-00633-9.
7. Reijns, M.A.M., Rabe, B., Rigby, R.E., Mill, P., Astell, K.R., Lettice, L.A., Boyle, S., Leitch, A., Keighren, M., Kilanowski, F., et al. (2012). Enzymatic removal of ribonucleotides from DNA is essential for mammalian genome integrity and development. Cell *149*, 1008–1022. https://doi.org/10.1016/j.cell.2012.04.011.
8. Lodi, L., Melki, I., Bondet, V., Seabra, L., Rice, G.I., Carter, E., Lepelley, A., Martin-Niclós, M.J., Al Adba, B., Bader-Meunier, B., et al. (2021). Differential Expression of Interferon-Alpha Protein Provides Clues to Tissue Specificity Across Type I Interferonopathies. J. Clin. Immunol. *41*, 603–609. https://doi.org/10.1007/s10875-020-00952-x.
9. Varley, J.A., Andersson, M., Grant, E., Berretta, A., Zandi, M.S., Bondet, V., Duffy, D., Hunt, D., Piehl, F., Waters, P., et al. (2020). Absence of Neuronal Autoantibodies in Neuropsychiatric Systemic Lupus Erythematosus. Ann. Neurol. *88*, 1244–1250. https://doi.org/10.1002/ana.25908.
10. Livingston, J.H., Stivaros, S., van der Knaap, M.S., and Crow, Y.J. (2013). Recognizable phenotypes associated with intracranial calcification. Dev. Med. Child Neurol. *55*, 46–57. https://doi.org/10.1111/j.1469-8749.2012.04437.x.
11. Cuadrado, E., Michailidou, I., van Bodegraven, E.J., Jansen, M.H., Sluijs, J.A., Geerts, D., Couraud, P.O., De Filippis, L., Vescovi, A.L., Kuijpers, T.W., et al. (2015). Phenotypic variation in Aicardi-Goutières syndrome explained by cell-specific IFN-stimulated gene response and cytokine release. J. Immunol. *194*, 3623–3633. https://doi.org/10.4049/jimmunol.1401334.
12. van Heteren, J.T., Rozenberg, F., Aronica, E., Troost, D., Lebon, P., and Kuijpers, T.W. (2008). Astrocytes produce interferon-alpha and CXCL10, but not IL-6 or CXCL8, in Aicardi-Goutières syndrome. Glia *56*, 568–578. https://doi.org/10.1002/glia.20639.
13. Giordano, A.M.S., Luciani, M., Gatto, F., Abou Alezz, M., Beghè, C., Della Volpe, L., Migliara, A., Valsoni, S., Genua, M., Dzieciatkowska, M., et al. (2022). DNA damage contributes to neurotoxic inflammation in Aicardi-Goutières syndrome astrocytes. J. Exp. Med. *219*, e20211121. https://doi.org/10.1084/jem.20211121.
14. Akwa, Y., Hassett, D.E., Eloranta, M.L., Sandberg, K., Masliah, E., Powell, H., Whitton, J.L., Bloom, F.E., and Campbell, I.L. (1998). Transgenic expression of IFN-alpha in the central nervous system of mice protects against lethal neurotropic viral infection but induces inflammation and neurodegeneration. J. Immunol. *161*, 5016–5026. https://doi.org/10.4049/jimmunol.161.9.5016.
15. Campbell, I.L., Krucker, T., Steffensen, S., Akwa, Y., Powell, H.C., Lane, T., Carr, D.J., Gold, L.H., Henriksen, S.J., and Siggins, G.R. (1999). Structural and functional neuropathology in transgenic mice with CNS expression of IFN-alpha. Brain Res. *835*, 46–61. https://doi.org/10.1016/s0006-8993(99)01328-1.
16. Blank, T., Detje, C.N., Spieß, A., Hagemeyer, N., Brendecke, S.M., Wolfart, J., Staszewski, O., Zöller, T., Papageorgiou, I., Schneider, J., et al. (2016). Brain Endothelial- and Epithelial-Specific Interferon Receptor Chain 1 Drives Virus-Induced Sickness Behavior and Cognitive Impairment. Immunity *44*, 901–912. https://doi.org/10.1016/j.immuni.2016.04.005.
17. Wardlaw, J.M., Smith, E.E., Biessels, G.J., Cordonnier, C., Fazekas, F., Frayne, R., Lindley, R.I., O'Brien, J.T., Barkhof, F., Benavente, O.R., et al. (2013). Neuroimaging standards for research into small vessel disease and its contribution to ageing and neurodegeneration. Lancet Neurol. *12*, 822–838. https://doi.org/10.1016/S1474-4422(13)70124-8.
18. Koni, P.A., Joshi, S.K., Temann, U.A., Olson, D., Burkly, L., and Flavell, R.A. (2001). Conditional vascular cell adhesion molecule 1 deletion in mice: impaired lymphocyte migration to bone marrow. J. Exp. Med. *193*, 741–754. https://doi.org/10.1084/jem.193.6.741.
19. West, P.K., Viengkhou, B., Campbell, I.L., and Hofer, M.J. (2022). Microglia shield the murine brain from damage mediated by the cytokines IL-6 and IFN-α. Front. Immunol. *13*, 1036799. https://doi.org/10.3389/fimmu.2022.1036799.
20. West, P.K., McCorkindale, A.N., Guennewig, B., Ashhurst, T.M., Viengkhou, B., Hayashida, E., Jung, S.R., Butovsky, O., Campbell, I.L., and Hofer, M.J. (2022). The cytokines interleukin-6 and interferon-α induce distinct microglia phenotypes. J. Neuroinflammation *19*, 96. https://doi.org/10.1186/s12974-022-02441-x.
21. Morita, M., Stamp, G., Robins, P., Dulic, A., Rosewell, I., Hrivnak, G., Daly, G., Lindahl, T., and Barnes, D.E. (2004). Gene-targeted mice lacking the Trex1 (DNase III) $3' \rightarrow 5'$ DNA exonuclease develop inflammatory myocarditis. Mol. Cell. Biol. *24*, 6719–6727. https://doi.org/10.1128/MCB.24.15.6719-6727.2004.
22. Kavanagh, D., McGlasson, S., Jury, A., Williams, J., Scolding, N., Bellamy, C., Gunther, C., Ritchie, D., Gale, D.P., Kanwar, Y.S., et al. (2016). Type I interferon causes thrombotic microangiopathy by a dose-dependent toxic effect on the microvasculature. Blood *128*, 2824–2833. https://doi.org/10.1182/blood-2016-05-715987.

23. Rutherford, H.A., Kasher, P.R., and Hamilton, N. (2020). Dirty Fish Versus Squeaky Clean Mice: Dissecting Interspecies Differences Between Animal Models of Interferonopathy. Front. Immunol. *11*, 623650. https://doi.org/10.3389/fimmu.2020.623650.

24. van der Knaap, M.S., and Bugiani, M. (2017). Leukodystrophies: a proposed classification system based on pathological changes and pathogenetic mechanisms. Acta Neuropathol. *134*, 351–382. https://doi.org/10.1007/s00401-017-1739-1.

25. Barth, P.G., Walter, A., and van Gelderen, I. (1999). Aicardi-Goutières syndrome: a genetic microangiopathy? Acta Neuropathol. *98*, 212–216. https://doi.org/10.1007/s004010051071.

26. Klok, M.D., Bakels, H.S., Postma, N.L., van Spaendonk, R.M.L., van der Knaap, M.S., and Bugiani, M. (2015). Interferon-α and the calcifying microangiopathy in Aicardi-Goutières syndrome. Ann. Clin. Transl. Neurol. *2*, 774–779. https://doi.org/10.1002/acn3.213.

27. Zarekiani, P., Breur, M., Wolf, N.I., de Vries, H.E., van der Knaap, M.S., and Bugiani, M. (2021). Pathology of the neurovascular unit in leukodystrophies. Acta Neuropathol. Commun. *9*, 103. https://doi.org/10.1186/s40478-021-01206-6.

28. Rice, G.I., Meyzer, C., Bouazza, N., Hully, M., Boddaert, N., Semeraro, M., Zeef, L.A.H., Rozenberg, F., Bondet, V., Duffy, D., et al. (2018). Reverse-Transcriptase Inhibitors in the Aicardi–Goutières syndrome. N. Engl. J. Med. *379*, 2275–2277. https://doi.org/10.1056/NEJMc1810983.

29. Vanderver, A., Adang, L., Gavazzi, F., McDonald, K., Helman, G., Frank, D.B., Jaffe, N., Yum, S.W., Collins, A., Keller, S.R., et al. (2020). Janus Kinase Inhibition in the Aicardi-Goutières Syndrome. N. Engl. J. Med. *383*, 986–989. https://doi.org/10.1056/NEJMc2001362.

30. Viengkhou, B., Hong, C., Mazur, C., Damle, S., Gallo, N.B., Fang, T.C., Henry, K., Campbell, I.L., Kamme, F., and Hofer, M.J. (2024). Interferon-α receptor antisense oligonucleotides reduce neuroinflammation and neuropathology in a mouse model of cerebral interferonopathy. J. Clin. Invest. *134*, e169562. https://doi.org/10.1172/JCI169562.

31. Gentek, R., Ghigo, C., Hoeffel, G., Bulle, M.J., Msallam, R., Gautier, G., Launay, P., Chen, J., Ginhoux, F., and Bajénoff, M. (2018). Hemogenic Endothelial Fate Mapping Reveals Dual Developmental Origin of Mast Cells. Immunity *48*, 1160–1171.e5. https://doi.org/10.1016/j.immuni.2018.04.025.

32. Utz, S.G., See, P., Mildenberger, W., Thion, M.S., Silvin, A., Lutz, M., Ingelfinger, F., Rayan, N.A., Lelios, I., Buttgereit, A., et al. (2020). Early Fate Defines Microglia and Non-parenchymal Brain Macrophage Development. Cell *181*, 557–573.e18. https://doi.org/10.1016/j.cell.2020.03.021.

33. Lindahl, P., Johansson, B.R., Levéen, P., and Betsholtz, C. (1997). Pericyte loss and microaneurysm formation in PDGF-B-deficient mice. Science *277*, 242–245. https://doi.org/10.1126/science.277.5323.242.

34. Warnes, G.R., Bolker, B., Bonebakker, L., Gentleman, R., Liaw, W.H.A., Lumley, T., Maechler, M., Magnusson, A., Moeller, S., Schwartz, M., et al. (2016). gplots: Various R Programming Tools for Plotting Data. https://cran.r-project.org/web/packages/gplots/index.html.

35. Wickham, H. (2009). ggplot2: Elegant Graphics for Data Analysis (Springer Publishing Company, Incorporated).

36. Mi, H., and Thomas, P. (2009). PANTHER pathway: an ontology-based pathway database coupled with data analysis tools. Methods Mol. Biol. *563*, 123–140. https://doi.org/10.1007/978-1-60761-175-2_7.

37. Thomas, P.D., Ebert, D., Muruganujan, A., Mushayahama, T., Albou, L.P., and Mi, H. (2022). PANTHER: making genome-scale phylogenetics accessible to all. Protein Sci. *31*, 8–22. https://doi.org/10.1002/pro.4218.

38. Kassambara, A., Kosinski, M., Biecek, P., and Fabian, S. (2019). survminer: Drawing Survival Curves using 'ggplot2'. https://rpkgs.datanovia.com/survminer/reference/ggsurvplot.html.

39. Christensen, R.H.B. (2019). ordinal—Regression Models for Ordinal Data. https://cran.r-project.org/web/packages/ordinal/index.html.

40. Bates, D., Maechler, M., Bolker, B., Walker, S., Christensen, R.H.B., Singmann, H., Dai, B., Scheipl, F., and Grothendieck, G. (2019). Linear Mixed-Effects Models using 'Eigen' and S4. https://cran.r-project.org/web/packages/lme4/index.html.

41. Lenth, R., Singmann, H., Love, J., Buerkner, P., and Herve, M. (2019). emmeans: Estimated Marginal Means, Aka Least-Squares Means. https://rdrr.io/cran/emmeans/.

42. Kamphuis, E., Junt, T., Waibler, Z., Forster, R., and Kalinke, U. (2006). Type I interferons directly regulate lymphocyte recirculation and cause transient blood lymphopenia. Blood *108*, 3253–3261. https://doi.org/10.1182/blood-2006-06-027599.

43. Yona, S., Kim, K.W., Wolf, Y., Mildner, A., Varol, D., Breker, M., Strauss-Ayali, D., Viukov, S., Guilliams, M., Misharin, A., et al. (2013). Fate mapping reveals origins and dynamics of monocytes and tissue macrophages under homeostasis. Immunity *38*, 79–91. https://doi.org/10.1016/j.immuni.2012.12.001.

44. Detje, C.N., Meyer, T., Schmidt, H., Kreuz, D., Rose, J.K., Bechmann, I., Prinz, M., and Kalinke, U. (2009). Local type I IFN receptor signaling protects against virus spread within the central nervous system. J. Immunol. *182*, 2297–2304. https://doi.org/10.4049/jimmunol.0800596.

45. Li, W., Viengkhou, B., Denyer, G., West, P.K., Campbell, I.L., and Hofer, M.J. (2018). Microglia have a more extensive and divergent response to interferon-α compared with astrocytes. Glia *66*, 2058–2078. https://doi.org/10.1002/glia.23460.

46. Viengkhou, B., White, M.Y., Cordwell, S.J., Campbell, I.L., and Hofer, M.J. (2021). A novel phosphoproteomic landscape evoked in response to type I interferon in the brain and in glial cells. J. Neuroinflammation *18*, 237. https://doi.org/10.1186/s12974-021-02277-x.

47. Meyer, S., Woodward, M., Hertel, C., Vlaicu, P., Haque, Y., Kärner, J., Macagno, A., Onuoha, S.C., Fishman, D., Peterson, H., et al. (2016). AIRE-Deficient Patients Harbor Unique High-Affinity Disease-Ameliorating Autoantibodies. Cell *166*, 582–595. https://doi.org/10.1016/j.cell.2016.06.024.

48. Wiseman, S.J., Bastin, M.E., Jardine, C.L., Barclay, G., Hamilton, I.F., Sandeman, E., Hunt, D., Amft, E.N., Thomson, S., Belch, J.F.F., et al. (2016). Cerebral Small Vessel Disease Burden Is Increased in Systemic Lupus Erythematosus. Stroke *47*, 2722–2728. https://doi.org/10.1161/STROKEAHA.116.014330.

49. Staals, J., Makin, S.D.J., Doubal, F.N., Dennis, M.S., and Wardlaw, J.M. (2014). Stroke subtype, vascular risk factors, and total MRI brain small-vessel disease burden. Neurology *83*, 1228–1234. https://doi.org/10.1212/WNL.0000000000000837.

50. Schindelin, J., Arganda-Carreras, I., Frise, E., Kaynig, V., Longair, M., Pietzsch, T., Preibisch, S., Rueden, C., Saalfeld, S., Schmid, B., et al. (2012). Fiji: an open-source platform for biological-image analysis. Nat. Methods *9*, 676–682. https://doi.org/10.1038/nmeth.2019.

51. Rueden, C.T., Schindelin, J., Hiner, M.C., DeZonia, B.E., Walter, A.E., Arena, E.T., and Eliceiri, K.W. (2017). ImageJ2: ImageJ for the next generation of scientific image data. BMC Bioinformatics *18*, 529. https://doi.org/10.1186/s12859-017-1934-z.

52. Ximerakis, M., Lipnick, S.L., Innes, B.T., Simmons, S.K., Adiconis, X., Dionne, D., Mayweather, B.A., Nguyen, L., Niziolek, Z., Ozek, C., et al. (2019). Single-cell transcriptomic profiling of the aging mouse brain. Nat. Neurosci. *22*, 1696–1708. https://doi.org/10.1038/s41593-019-0491-3.

53. Saunders, A., Macosko, E.Z., Wysoker, A., Goldman, M., Krienen, F.M., de Rivera, H., Bien, E., Baum, M., Bortolin, L., Wang, S., et al. (2018). Molecular Diversity and Specializations among the Cells of the Adult Mouse Brain. Cell *174*, 1015–1030.e16. https://doi.org/10.1016/j.cell.2018.07.028.

54. Franzén, O., Gan, L.M., and Björkegren, J.L.M. (2019). PanglaoDB: a web server for exploration of mouse and human single-cell RNA sequencing data. Database (Oxford) *2019*, baz046. https://doi.org/10.1093/database/baz046.

55. Krämer, A., Green, J., Pollard, J., Jr., and Tugendreich, S. (2014). Causal analysis approaches in Ingenuity Pathway Analysis. Bioinformatics Oxf. Engl. *30*, 523–530. https://doi.org/10.1093/bioinformatics/btt703.

CellPress
OPEN ACCESS

56. R Core Team (2020). R: A language and environment for statistical computing (R Foundation for Statistical Computing).
57. Yang, B., Treweek, J.B., Kulkarni, R.P., Deverman, B.E., Chen, C.K., Lubeck, E., Shah, S., Cai, L., and Gradinaru, V. (2014). Single-cell phenotyping within transparent intact tissue through whole-body clearing. Cell *158*, 945–958. https://doi.org/10.1016/j.cell.2014.07.017.
58. Hernández, P.P., Mahlakoiv, T., Yang, I., Schwierzeck, V., Nguyen, N., Guendel, F., Gronke, K., Ryffel, B., Hoelscher, C., Dumoutier, L., et al. (2015). Interferon-λ and interleukin 22 act synergistically for the induction of interferon-stimulated genes and control of rotavirus infection. Nat. Immunol. *16*, 698–707. https://doi.org/10.1038/ni.3180.
59. Doughty, M.J. (2010). pH dependent spectral properties of sodium fluorescein ophthalmic solutions revisited. Ophthalmic Physiol. Opt. *30*, 167–174. https://doi.org/10.1111/j.1475-1313.2009.00703.x.
60. Kaya, M., and Ahishali, B. (2011). Assessment of permeability in barrier type of endothelium in brain using tracers: Evans Blue, sodium fluorescein, and horseradish peroxidase. Methods Mol. Biol. *763*, 369–382. https://doi.org/10.1007/978-1-61779-191-8_25.
61. Tung, V.W.K., Burton, T.J., Dababneh, E., Quail, S.L., and Camp, A.J. (2014). Behavioral assessment of the aging mouse vestibular system. J. Vis. Exp. *89*, 51605. https://doi.org/10.3791/51605.

STAR★METHODS

KEY RESOURCES TABLE

REAGENT or RESOURCE	SOURCE	IDENTIFIER
Antibodies		
Rabbit polyclonal anti-Iba1	Wako	Cat#019-19741; RRID:AB_839504
Rabbit polyclonal anti-GFAP	Dako	Cat#Z0334; RRID:AB_10013382
Mouse monoclonal anti-neurofilament	Sigma-Aldrich	Cat#N0142; RRID:AB_477257
Rabbit monoclonal anti-CD3	Abcam	Cat#ab16669; RRID:AB_443425
Rabbit polyclonal anti-cleaved caspase-3 (Asp175)	Cell Signaling Technology	Cat#9661; RRID:AB_2341188
Rabbit monoclonal anti-cre recombinase	Cell Signaling Technology	Cat#15036; RRID:AB_2798694
Goat polyclonal anti-rabbit IgG, biotinylated	Vector Laboratories	Cat#BA-1000; RRID:AB_2313606
Rabbit anti-rat IgG, biotinylated	Vector Laboratories	Cat#BA-4001; RRID:AB_10015300
Horse polyclonal anti-mouse IgG, biotinylated	Vector Laboratories	Cat#BA-2000; RRID:AB_2313581
Mouse anti-ICAM1	Dr. F. Takei (Terry Fox Laboratory, British Columbia Cancer Research Centre)	N/A
Mouse monoclonal anti-MHCI	ATCC	Cat# TIB-126; RRID:CVCL_9205
Rabbit polyclonal anti-laminin	Sigma-Aldrich	Cat#L9393; RRID:AB_477163
Rabbit monoclonal anti-STAT1 (Tyr701)	Cell Signaling Technology	Cat#7649; RRID:AB_10950970
Rabbit polyclonal anti-STAT1 (Ser727)	Cell Signaling Technology	Cat#9177; RRID:AB_2197983
Rabbit polyclonal anti-STAT1	Cell Signaling Technology	Cat#9172; RRID:AB_2198300
Mouse monoclonal anti-GAPDH	Merck Millipore	Cat#MAB374; RRID:AB_2107445
Goat polyclonal anti-rabbit IgG-horseradish peroxidase	Santa Cruz Biotechnology	Cat#sc-2004; RRID:AB_631746
Goat polyclonal anti-mouse IgG-peroxidase	Sigma-Aldrich	Cat#A0168; RRID:AB_257867
Rat monoclonal anti-CD16/32	BD Biosciences	Cat#553142; RRID:AB_394657
Rat monoclonal anti-CD31-BV421	Biolegend	Cat#102423; RRID:AB_2562186
Rat monoclonal anti-CD11b-BV650	Biolegend	Cat#101239; RRID:AB_11125575
Rat monoclonal anti-CD45-FITC	BD Biosciences	Cat#553080; RRID:AB_394610
Rat monoclonal anti-ACSA-2-PE	Miltenyi Biotec	Cat#130-123-284; RRID:AB_2811488
Rat anti-CD90.2-APC	BD Biosciences	Cat#561974; RRID: AB_398526
Human monoclonal anti-IFN-α, clone 8H1	Lodi et al.[5]	N/A
Human monoclonal anti-IFN-α, clone 12H5	Lodi et al.[5]	N/A
Chemicals, peptides, and recombinant proteins		
Human IFN-Alpha 17 (Alpha I) Protein	PBL Assay Science	Cat#11150
Formaldehyde solution	Sigma-Aldrich	Cat#F8775
Paraformaldehyde	Sigma-Aldrich	Cat#P6148
PBS (10X), pH 7.4	Thermo Fisher Scientific	Cat#70011044
Histodenz (iohexol)	Sigma-Aldrich	Cat#D2158
Dulbecco's Phosphate Buffered Saline	Sigma-Aldrich	Cat#D1408
Bovine serum albumin	Sigma-Aldrich	Cat#A7906
Trypan Blue solution	Sigma-Aldrich	Cat#T8154
Alizarin Red S	Sigma-Aldrich	Cat#A5533
Hydrogen peroxide solution	Sigma-Aldrich	Cat#H1009
Goat serum	Sigma-Aldrich	Cat#G9023
Tween-20	Promega	Cat#H5152
Triton-X100	Sigma-Aldrich	Cat#T8787
Mayer's Hematoxylin Solution	Sigma-Aldrich	Cat#MHS16

(Continued on next page)

CellPress
OPEN ACCESS

Continued

REAGENT or RESOURCE	SOURCE	IDENTIFIER
DPX Mountant for histology	Sigma-Aldrich	Cat#06522
Acrylamide Solution, Acryl-40	Amresco	Cat#0132
Acrylamide Solution, Bis-2	Amresco	Cat#0832
VA-044	Wako	Cat#223-02112
Sodium azide	Sigma-Aldrich	Cat#71289
Streptavidin, Alexa Fluor 488 conjugate	Life Technologies	Cat#S11223
Hoechst 33342	Sigma-Aldrich	Cat#B2261
TRI Reagent	Sigma-Aldrich	Cat#93289
Tris-EDTA buffer solution	Sigma-Aldrich	Cat#93302
RQ1 RNase-Free DNase	Promega	Cat#M6101
Fluorescein sodium salt	Sigma-Aldrich	Cat#166308
Evans blue	Sigma-Aldrich	Cat#E2129
Tissue-Tek O.C.T. Compound	ProSciTech	Cat#IA018
2-methylbutane	Sigma-Aldrich	Cat#320404
trichloroacetic	Sigma-Aldrich	Cat#91230
Protease Inhibitor Cocktail III	Merck Millipore	Cat#539134
Phosphatase Inhibitor Cocktail II	Merck Millipore	Cat#524625
Ponceau S solution	Sigma-Aldrich	Cat#P7170
Immobilon Western Chemiluminescent HRP Substrate	Merck Millipore	Cat#WBKLS0500
LIVE/DEAD Fixable Blue Dead Cell Stain Kit	Thermo Fisher Scientific	Cat#L34961
RNeasy Micro Kit	Qiagen	Cat#74004
Mouse IFN-α	Miltenyi Biotech	Cat#130-093-130
Chromium Single Cell 3′ Library and Gel Bead Kit v3	10X Genomics	Cat#1000075
VECTASTAIN Elite ABC-HRP Kit, Peroxidase (Standard)	Vector Laboratories	Cat#PK-6100
DAB Substrate Kit, Peroxidase (HRP), with Nickel	Vector Laboratories	Cat#SK-4100
Avidin/Biotin Blocking Kit	Vector Laboratories	Cat#SP-2001
Vector NovaRED Substrate Kit, Peroxidase	Vector Laboratories	Cat#SK-4800
Mix-n-Stain CF 647 Antibody Labeling Kit	Sigma-Aldrich	Cat#MX647S50
Mix-n-Stain CF 568 Antibody Labeling Kit	Sigma-Aldrich	Cat#MA568S50
BSA Removal Kit	Abcam	Cat#ab173231
RevertAid RT Reverse Transcription Kit	Thermo Fisher Scientific	Cat#K1691
SensiFAST SYBR Lo-ROX Kit	Bioline	Cat#BIO-94020
Pierce BCA Protein Assay Kit	Thermo Fisher Scientific	Cat#23225
Minimum Essential Medium Eagle	Sigma-Aldrich	Cat#M2279
HEPES solution	Sigma-Aldrich	Cat#H0887
Papain from papaya latex	Sigma-Aldrich	Cat#P3125
L-cysteine	Sigma-Aldrich	Cat#C7352
Deoxyribonuclease I from bovine pancreas	Sigma-Aldrich	Cat#D5025
Fetal Bovine Serum	Sigma-Aldrich	Cat#12003C
Penicillin-Streptomycin (10,000 U/mL)	Thermo Fisher Scientific	Cat#15140163
Poly-D-Lysine solution	Merck Millipore	Cat#A-003-E
Adult Brain Dissociation Kit	Miltenyi Biotec	Cat#130-107-677
Ammonium Chloride Solution	Stemcell Technologies	Cat#07850
**Critical commercial assays**		
Quanterix Homebrew Simoa Kit	Quanterix	Cat#101354
Mouse Interferon alpha 1 ELISA Kit	Abcam	Cat#ab252352

(*Continued on next page*)

Continued		
REAGENT or RESOURCE	SOURCE	IDENTIFIER
Deposited data		
scRNASeq data	This paper	ENA: PRJEB44230
Experimental models: Organisms/strains		
Mouse: GIFN mice (B6;C-Tg(Gfap-Ifna1)39llc/Niusy)	Akwa et al.[14]; Campbell et al.[15]	RRID:MGI:7328531
Mouse: IFNAR1 floxed (Ifnar1^{tm1Uka})	Dr Peter Crack (University of Melbourne)	MGI:2655303
Mouse: Tek-Cre (B6.Cg-Tg(Tek-cre)12Flv/J)	The Jackson Laboratory	RRID:IMSR_JAX:004128
Mouse: Cx3cr1-Cre (B6J.B6N(Cg)-Cx3cr1tm1.1(cre)Jung/J)	The Jackson Laboratory	RRID:IMSR_JAX:025524
Mouse: C57BL/6J	Australian BioResource	C57BL/6JAusb
Oligonucleotides		
See Table S5		N/A
Software and algorithms		
Fiji	NIH	https://fiji.sc/; RRID:SCR_002285
Cell Ranger v3.0.2, v3.1.0	10X Genomics	https://www.10xgenomics.com/support/software/cell-ranger/downloads/; RRID:SCR_017344
Loupe Browser v4	10X Genomics	https://www.10xgenomics.com/support/software/loupe-browser/downloads; RRID:SCR_018555
R v3.6.1, v4.0.2	R	https://www.r-project.org/; RRID: SCR_002394
gplots v3.1.0	Warnes et al.[34]	https://cran.r-project.org/web/packages/gplots/
ggplot2 v3.3.2	Wickham[35]	https://cran.r-project.org/web/packages/ggplot2/index.html
Panther v18.0	Mi and Thomas[36]; Thomas et al.[37]	https://pantherdb.org/; RRID:SCR_004869
SPOT Advanced 4.5	Spot Imaging	http://www.spotimaging.com/software/spot-advanced/; RRID:SCR_016613
Imaris v9	Oxford Instruments	http://www.bitplane.com/imaris/imaris; RRID:SCR_007370
TreadScan	CleverSys	https://cleversysinc.com/CleverSysInc/csi_products/treadscan/
GraphPad Prism v8 and v9	GraphPad Software	http://www.graphpad.com/; RRID:SCR_002798
survminer v0.4.6	Kassambara et al.[38]	https://CRAN.R-project.org/package=survminer; RRID:SCR_021094
ordinal v2019.12-10	Christensen[39]	https://CRAN.R-project.org/package=ordinal; RRID:SCR_022856
lme4 v1.1-21	Bates et al.[40]	https://CRAN.R-project.org/package=lme4; RRID:SCR_015654
emmeans v1.4.3	Lenth et al.[41]	https://CRAN.R-project.org/package=emmeans; RRID:SCR_018734
FlowJo v10	BD Biosciences	https://www.flowjo.com/solutions/flowjo; RRID:SCR_008520
Other		
UltraComp eBeads™ Compensation Beads	Thermo Fisher Scientific	Cat#01-2222-41
Latex beads, carboxylate-modified polystyrene, fluorescent yellow-green	Sigma-Aldrich	Cat#L4655

(*Continued on next page*)

Continued

REAGENT or RESOURCE	SOURCE	IDENTIFIER
Myelin Removal Beads II	Miltenyi Biotec	Cat#130-096-733
CD31 MicroBeads	Miltenyi Biotec	Cat#130-097-418; RRID:AB_2814657

RESOURCE AVAILABILITY

Lead contact

Further information and requests for resources and reagents should be directed to and will be fulfilled by the lead contact, Markus Hofer (markus.hofer@sydney.edu.au).

Materials availability

This study did not generate new unique reagents.

Data and code availability

- The scRNASeq data have been deposited to the European Nucleotide Archive (ENA; https://www.ebi.ac.uk/ena/browser/home) and are publicly available from the date of publication. Accession number is listed in the key resources table.
- This paper does not report original code.
- Any additional information required to reanalyze the data reported in this paper is available from the lead contact upon request.

EXPERIMENTAL MODELS AND STUDY PARTICIPANT DETAILS

Human samples

Study procedures and sample testing were ethically approved (Healthy control, RRMS and SLE: Stockholm: 2009/2107-31/2; SLE Oxford: REC16/YH/0013, and Leeds (East) REC: 10/H1307/2; Integrated Research Approval System project ID: 62971 in the UK). Computed Tomography (CT) and angiographic images used were provided with informed consent from individuals or their relatives. The SLE brain study received research ethics committee approval (South-East Scotland Research Ethics Committee 01, 14/SS/0003), and all participants gave written informed consent. Demographics provided in Table S4.

Use of formalin-fixed paraffin embedded human brain sections complied with the National Statement on Ethical Conduct in Human Research and the Australian Code for the Responsible Conduct of Research. Ethics approval was granted by the University of Sydney Human Research Ethics Committee (2019/492).

Mice

All animal experiments were performed in compliance with the NSW Animal Research Act and its associated regulations and the 2013 NHMRC 'Australian code of practice for the care and use of animals for scientific purposes'. Ethical approval was granted by the University of Sydney Animal Care and Ethics Committee (2014/699, 2018/1373, 2023/2322 and 2023/2270). All mice were housed and maintained under specific pathogen-free conditions in the animal house facility in the School of Life and Environmental Sciences, The University of Sydney, receiving food and water *ad libitum*. Mouse lines were maintained by inbreeding and include GIFN mice (B6;C-Tg(Gfap-Ifna1)39Ilc/Niusy MGI:7328531),[14,15] originally obtained from the Scripps Research Institute, La Jolla, CA, USA, where they were developed by I. L. Campbell, IFNAR1 floxed mice[42] and Tek-Cre mice[18] (JAX stock #004128). The risk of germline activity leading to global knockout was minimised by using only male *Tek-Cre* mice. Generation of GIC mice was done analogous to GIT mice. We observed Ifnar1 recombination in some *Cx3cr1-Cre* mice[43] (JAX stock #025524), with approximately 15% of GIFN$^{+/-}$ x *Ifnar1*$^{fl/fl}$ x *Cx3cr1-Cre*$^{-/-}$ and GIFN$^{+/-}$ x *Ifnar1*$^{fl/fl}$ x *Cx3cr1-Cre*$^{+/-}$ being half-global knockout for *Ifnar1*. All mice were genotyped for the presence of *Ifnar1* recombination.[44] Due to breeding complexities and limited mouse availabilities, mice of both sexes were used (or otherwise specified) for experiments when available with appropriate littermate controls. Age of mice is included in results and figure legends.

Mice were weighted weekly and scored: 0 = WT-like, 0.5 = minor ataxia, 1 = altered gait, 1.5 = severely altered gait, 2 = ataxia, 2.5 = reduced activity, 3 = wild running or jumping, 3.5 = absent seizure, 4 = brief convulsive seizure with recovery, 5 = continuous convulsive seizure or found dead. A score of 5 meet euthanasia criteria. Mice were euthanized using isoflurane (IsoFlo®, Abbott Laboratories).

METHOD DETAILS

Primary Cultures

Primary microglia were isolated from P0-4 WT C57Bl/6 mice as previously described.[45,46] Briefly, cortices without meninges were diced in ice cold minimum essential medium (M2279, Sigma) containing 25 mM HEPES (H0887, Sigma) and then dissociated in 1 mg/ml papain (P3125, Sigma-Aldrich) solution containing 240 μg/ml L-cysteine (C7352, Sigma-Aldrich), 1140 U DNase I type IV

(D5025, Sigma-Aldrich), 25 mM HEPES in minimum essential medium for 1 h at 37°C. A single cell suspension was generated by triturating the dissociated tissues in DF media [10% FBS (12003C, Sigma-Aldrich), 100 U/ml penicillin and 100 μg/ml streptomycin (15140163, Thermo Fisher Scientific) in DMEM (D6429, Sigma-Aldrich)]. After washing the cells with DF media, cells were seeded on poly-D-lysine (A-003-E, Merck Millipore) coated flasks and incubated at 37°C with 5% CO_2 in a humidified incubator. To isolate microglia, confluent flasks were shaken at 260 rpm for 4 h at 37°C and the supernatant pooled and counted before seeding in plates for subsequent experiments.

Simoa study

Control plasma and CSFs were from 19 healthy controls, and from 32 individuals with relapsing-remitting multiple sclerosis (Table S4A). Paired plasma and CSF samples were obtained from individuals with AGS and individuals who met American College of Rheumatology (ACR) criteria for SLE, with clinical details provided in earlier publications.[8,9] Additional paired blood-CSF IFN-α concentrations from individuals with SLE and AGS were systematically analyzed from primary data contained within,[8,9] as well as analyzes of non-Simoa biomarkers from the largest published cohort.[5] IFN-α Simoa was performed as detailed here[8]: the Simoa IFNα assay was developed using a Quanterix Homebrew Simoa assay according to the manufacturer's instructions, and utilizing two autoantibodies specific for pan-IFNα subtype isolated and cloned from two individuals with APS1 / APECED as described.[47] The 8H1 antibody clone was used as a capture antibody after coating on paramagnetic beads (0.3 mg/mL), and the 12H5 was biotinylated (biotin / Ab ratio = 30 / 1) and used as the detector. Recombinant IFNα17/αI (PBL Assay Science) was used to produce a standard curve after cross-reactivity testing. The limit of detection was calculated as the mean value of all blank runs +3 SD and was 0.23 fg/ml. IFN-α concentrations in serum and plasma were considered comparable as previously demonstrated. IFN-α concentrations in the CSF were considered normal for values beneath 2 fg/ml and in serum/plasma beneath 10 fg/ml. All assays were performed blinded to clinical details.

Time to onset analyses

Primary data for individuals with AGS across 6 different genotypes was identified from published datasets.[5] We identified individuals where data was available for time to onset (age in months of lumbar puncture), together with CSF and serum IFN-α concentrations/activity. Age at lumbar puncture and paired CSF and serum IFN-α data was available for 105 individuals. Multiple linear regression was performed using Prism 9.

SLE brain MRI study

This cross-sectional brain magnetic resonance imaging (MRI) study prospectively recruited individuals with SLE—including members of the Scottish Lupus Exchange Database (UK Clinical Trials ID 15489)—who attended a regional specialist clinic (Table S4B). The clinic reviews all individuals diagnosed with SLE in one health region from the point of diagnosis onward. We recruited as consecutively as possible, and individuals with SLE represented a wide range of SLE, being of varying disease durations and severities. All individuals were seen by a consultant rheumatologist; clinics were run jointly with a neurologist and renal physician. SLE was diagnosed according to updated American College of Rheumatology 1997 criteria.

MRI image review and visual rating

All MRI scans were reviewed by a consultant neuroradiologist blind to all other data. Imaging features of cerebral small vessel disease (SVD) were defined per STRIVE guidelines and analyses performed as previously described.[48] Deep and periventricular white matter hyperintensities (WMHs) were coded 0 to 3 using the Fazekas scale and summed to give a total WMH score (0–6) per subject. Visible (enlarged) perivascular spaces (PVS) are round (<3 mm) or linear depending on the orientation of the scan plane to the vessel and their intensity is that of cerebrospinal fluid on T2-weighted MRI. They were assessed in the basal ganglia and centrum semiovale and scored as 0 (none), 1 (1–10 PVS), 2 (11–20 PVS), 3 (21–40 PVS), and 4 (>40 PVS) using a validated scale. Lacunes were defined as deep infarcts, distinguished from PVS because of their larger size (3–20 mm), and their presence, including location in the brain, was noted and burden assessed by total count. We used the gradient-recalled echo scans and the simplified Brain Observer Microbleeds Scale to count microbleeds. Cerebral atrophy was defined as enlargement of the ventricles (deep atrophy) and enlargement of the sulci (superficial atrophy) and scored accordingly by classifying each participant on a validated 6-point scale against a template of normal reference brains. Three analysts did the rating; inter-rater agreement (κ) was 0.66 to 1.0.

Total SVD score

A total SVD score[49] (range 0–4) was calculated from individual imaging features by awarding points as follows: 1 for any lacunes, 1 for any microbleeds, 1 for moderate-to-severe PVS in the basal ganglia (grade 2–4), and 1 for WMHs (deep tissue: Fazekas score 2 or 3 and/or periventricular: Fazekas score 3).

Micro-CT

Mice were transcardially perfused with ice cold 4% buffered formaldehyde at 120 mmHg and a flow rate of 3 ml/min for 10 min and post-fixed overnight at 4°C. Skin and muscle was removed to leave the skull and brain intact, which was subsequently washed in 1x PBS before incubation in 5.5% iohexol (D2158, Sigma-Aldrich) in 1x PBS for 10 days. Skulls were briefly washed with water and then imaged using the MILabs U-CT at Sydney Imaging (University of Sydney) with step angle of 0.25 degrees, tube voltage of 50 kV, tube

current of 0.24 mA and exposure time of 75 ms. Three-dimensional reconstruction was performed using MILabs Recon (v9) with a voxel size of 10 μm. Image processing was performed with Fiji.[50,51]

Single cell isolation and library preparation

Mice (four-week-old female littermates; *N* = 2 WT and 4 GIFN) were perfused with ice cold 1x DPBS (D1408, Sigma-Aldrich), meninges removed and forebrains collected. Tissues were processed as per manufacturer's instructions of the Adult Brain Dissociation Kit (130-107-677, Miltenyi Biotec). Tissues were pooled at the enzymatic dissociation step. Before the cell debris removal step, myelin depletion (130-096-733, Miltenyi Biotec) was performed using the AutoMACS per manufacturer's instructions. Running buffer consisted of 0.5% bovine serum albumin (BSA; A7906, Sigma-Aldrich) in 1x DBPS and filtered through 0.22 μm (SLGP033RS, Merck Millipore). A portion of the single cells were enriched using CD31-microbeads (130-097-418, Miltenyi Biotec) as per manufacturer's instructions. Cells were counted manually and automatically (Countess II, Thermo Fisher Scientific) with trypan blue and adjusted to 1000 cells/μl. Single cells (WT and GIFN total and WT and GIFN enriched) were processed on the same fluidics chip with the Chromium Single Cell 3′ Library and Gel Bead Kit v3 (10X Genomics) following manufacturer's instructions to capture 10,000 cells per sample.

Single cell RNA sequencing analysis and bioinformatics

Sequencing was performed on two lanes of an Illumina NovaSeq 6000 S1 flowcell. Fastq files were generated using the 'mkfastq' tool 10X Genomics' Cell Ranger software (v3.0.2), which runs Illumina's "bcl2fastq" software with the following arguments: " –minimum-trimmed-read-length 8 –mask-short-adapter-reads 8 –create-fastq-for-index-reads –ignore-missing-positions –ignore-missing-filter –ignore-missing-bcls –use-bases-mask=Y28,I8,Y91 -R -p 6 -r 6 -w 6". Sequences were processed using Cell Ranger v3.1.0 (10X Genomics) pipeline and mapped to mm10-3.0.0. The output summary revealed 38,291, 53,737, 51,368 and 27,540 mean reads per cell with 2,582, 2,904, 3,178 and 2,705 median genes per cells, respectively, for WT total, GIFN total, WT enriched and GIFN enriched datasets. The four data sets were aggregated in the pipeline for subsequent visualization and analysis with Loupe Browser v4 (10X Genomics).

Quality control was first performed. Graph-based clusters that had zero or fewer than ten globally distinguishing significant features ($P \leq 0.05$) were excluded. To remove doublets, cells with more than 30,000 unique molecular identifiers were excluded as in.[52] Known cell-type markers[52,53] were used to classify the identity of cells and doublets: vascular cells (*Flt1* and *Cldn5*), microglia (*P2ry12* and *Tmem119*), astrocytes (*Aqp4* and *Gja1*), oligodendrocytes (*Cldn11* and *Plp1*), immature neurons (*Dcx* and *Sox11*), mural cells (*Acta2*), pericytes (*Pdgfrb* and *Kcnj8*), ependymal cells (*Ccdc153*), choroid plexus epithelial cells (*Ttr*), T and B cells (*Cd3* and *Cd19*, respectively), monocytes (*Plac8*), dendritic cells (*Cd209*) and erythrocytes (*Alas2*, *Hbb-bt* and *Hba-a1*). Endothelial cells were identified with vascular cell markers and were negative for pericyte and mural cell markers. Identified doublets co-expressed markers in three visually segregated clusters (*Flt1* with *Tmem119* or *Plac8* and *Tmem119* with *Aqp4*) or co-expressed markers with high counts (*Flt1* $\geq$ 8 and *Aqp4* $\geq$ 3; *Flt1* $\geq$ 7 and *Plp1* $\geq$ 82; *Tmem119* $\geq$ 5 and *Ccdc153* $\geq$ 5). Groups of cells that were spatially separated on the UMAP plot but associated with one graph-based cluster were split into separate clusters. Additionally, clusters were excluded if mitochondrial genes were the only significant globally distinguishing features, indicating low-quality cells.

To assign cell identities to the remaining graph-based clusters, the top significant or abundant globally distinguishing features in each cluster were used to unbiasedly verify cell identities[54] which were assigned by known cell-type markers (Figure S1D). Subsequently, total and enriched cells were separately analyzed. Differential feature analyses were performed using Loupe Brower with Local Distinguishing Feature Comparison between WT and GIFN cells of a cell identity. After statistical analysis, the software excluded a feature if the average count of that feature from the analyzed cell identity in both genotypes was below one. For downstream analysis, features were considered significantly regulated if adjusted $P \leq 0.05$ and $|\text{fold change}| \geq 2$. Pathway analysis was performed using Ingenuity Pathway Analysis[55] and filtered for significantly regulated features with species set to mouse. Heatmap and plots were generated in R[56] using gplots[34] and ggplot2.[35]

To further delineate the regulated pathways in microglia from GIFN mice, we obtained the significantly regulated genes in our dataset and those from bulk-sequenced microglia[20] and parsed them through Panther[36,37] to compare enrichment and non-enrichment of various ontology terms of interest.

Histopathology

Brains from mice were fixed overnight in 10% neutral buffered formalin (F8775, Sigma-Aldrich; diluted using 1x PBS) and processed into 5 μm paraffin sections. Paraffin sections were deparaffinized and rehydrated through an ethanol series prior to histological stains or immunohistochemistry. Histological stains of H&E and LFB&CV were performed at the Histopathology Facility (Department of Pathology, The University of Sydney) for mouse sections and the Department of Neuropathology (University of Marburg, Germany) for human sections. Demonstration of calcification was shown with 2% alizarin red S (pH 4.2) (A5533, Sigma-Aldrich) for 1 min. For immunohistochemical stains, antigens were retrieved in a steamer for 45 min in 25 mM Tris-HCl (pH 9), 10 mM sodium citrate buffer (pH 6) or 25 mM Tris pH 8, 5 mM EDTA with 0.05% SDS. After blocking peroxidases with 0.3% H_2O_2 (H1009, Sigma-Aldrich) in 1x PBS, sections were blocked in 1% goat serum (G9023, Sigma-Aldrich) in 0.1% Tween-20 (H5152, Promega) and 0.05% Triton-X (T8787, Sigma-Aldrich) in 1x PBS for 20 min. Primary antibodies [Iba1 (1:1,000, Tris-EDTA-SDS; 019-19741, Wako), GFAP (1:1,000, Tris pH 9; Z0334, Dako), neurofilament (1:400; N0142, Sigma-Aldrich, Tris-EDTA-SDS), CD3 (1:200, citrate buffer; ab16669, Abcam), cleaved caspase-3 (1:400, citrate buffer; 9661, CST) and Cre recombinase (1:125, citrate buffer; 15036, CST)]

diluted in blocking buffer were applied overnight at 4°C in a humidified chamber. After washing in 1x PBS, sections were incubated with the corresponding biotinylated secondary antibodies [rabbit IgG-biotinylated (BA-1000), rat IgG-biotinylated (BA-4001) and mouse IgG-biotinylated (BA-2000; Tris-EDTA-SDS when used as a primary antibody) with 1:200 dilution, all from Vector Laboratories] for 1 h prior to the application of the ABC kit (PK-6100, Vector Laboratories). Antigen location was revealed with 3,3'-diaminobenzidine or enhanced by nickel (SK-4100, Vector Laboratories) and nuclei were visualized with Mayer's hematoxylin (MHS16, Sigma-Aldrich). To colocalize Cre recombinase, slides were subsequently treated with avidin/biotin (SP-2001, Vector Laboratories) and then incubated with tomato lectin (1:50; L0651, Sigma Aldrich) for 30 min at RT. After washing, lectin was detected with NovaRED (SK-4800, Vector Laboratories) before counterstaining with Mayer's hematoxylin. Slides were subsequently dehydrated through an ethanol series and coverslipped with DPX (06522, Sigma-Aldrich). Fresh frozen sections (10 μm) were obtained after blood-brain barrier leakage experiments using a cryostat (CM1850, Leica), air dried and stored at -80°C. Sections were fixed in acetone:ethanol (3:1) for 5 min and then rinsed in water prior blocking and incubation with primary antibodies [ICAM1 (1:100; kindly provided by Dr. F. Takei, Terry Fox Laboratory, British Columbia Cancer Research Centre, Vancouver, Canada) and MHCI (neat; M1/42.3.9.8.HLK, ATCC)]. Sections were viewed through a DM4000B microscope (Leica, Macquarie Park, NSW, Australia) and images were acquired with a SPOT Flex 64 MP camera and SPOT Advanced 4.5 (Spot Imaging). Slides were scanned in a ZEISS Axio Scan.Z1 with a Plan-Apochromat 10x/0.45 M27 objective and acquired using ZEISS Zen slidescan at the Advanced Microscopy Facility (The Bosch Institute, The University of Sydney). Human brain slices were stained as described above and images were taken with an Olympus BX41 microscope (Olympus, Hamburg, Germany) equipped with a Bresser MicroCam II 4k using the manufacturer's software (Bresser, Rhede, Germany). For visual clarity, brightness and contrast was adjusted to the entire image. Images were quantified using Fiji.[50,51]

Passive tissue clearing

To obtain three-dimensional images of large sections through the brain, mouse brains were passively cleared to achieve optical transparency, similarly described in Yang et al.[57] Briefly, mice were transcardially perfused with ice cold 4% buffered formaldehyde at 120 mmHg and a flow rate of 3 ml/min for 10 min and post-fixed overnight at 4°C. Brains were washed and infused with hydrogel [4% acrylamide (0132, Amresco), 0.05% bisacrylamide (0832, Amresco), 0.25% VA-044 (223-02112, Wako) in 1x PBS] at 4°C before polymerization at 37°C for 4 h in a water bath. Excess polyacrylamide was removed and lipids were cleared by shaking at 260 rpm at 37°C in 8% SDS in 1x PBS. Passively cleared brains were washed multiple times with 1x PBS throughout a day before incubation with Iba1 (1:100 dilution) in diluent [2% goat serum, 0.1% Triton-X, 0.02% NaN_3 (71289, Sigma-Aldrich) in 1x PBS] for 7 days on a shaker. Excess antibodies were removed with multiple washes in 1x PBS over a day on a shaker. Biotinylated anti-rabbit IgG (1:200 dilution) was subsequently applied, incubated for 7 days on a shaker, washed and followed by staining with laminin (L9393, Sigma-Aldrich) conjugated to CF647 (MX647S50, Sigma-Aldrich) (diluted to 6.25 μg/ml), GFAP conjugated to CF568 (MA568S50, Sigma-Aldrich) (diluted to 29 μg/ml) all in the diluent. The brain was again washed and similarly incubated with strepavidin-AF488 (1:300 dilution; S11223, Life Technologies) and 1 μg/ml Hoechst 33342 (B2261, Sigma-Aldrich) all in the diluent. BSA was removed from laminin using the BSA Removal Kit (ab173231, Abcam) prior to conjugation. Immunostained brains were washed over a day and optically cleared and stored in 88% Histodenz (D2158, Sigma-Aldrich) in 0.1 M phosphate buffer (76.8 mM Na_2HPO_4 and 22.4 mM NaH_2PO_4), 1 μg/ml Hoechst 33342 and 0.02% NaN_3 until imaging.

Single cell morphometric quantification

Brains were imaged on a glass bottom microwell dish (P35G-1.5-20-C, MatTek) using a laser scanning confocal microscope (LSM800; Zeiss) at the Advanced Microscopy Facility (The Bosch Institute). Z-stacks were acquired through a Plan-Apochromat 10x/0.45 M27 or a 20x/0.8 M27 with lasers at 405, 488, 561 and 640 nm and emission collected through 400-497 and 563-617 nm on one track and 410-563 and 640-700 nm on the other track. Laser power, gain and offset were interpolated through the z-stack to maintain signal intensity. Z-stacks were acquired with bidirectional scanning, an averaging of 2, 1.03 μs pixel time and 50% overlap between slices. Imaris (v9) (Oxford Instruments) was used to extract morphological information of vessels, astrocytes and microglia. Vessels were divided into vascular segments, defined as a portion of the vessel between branching points. Noise in the z-stack was smoothed using a median filter at 3x3x1. Filament tracer was used to manually trace vascular segments and processes of astrocytes and microglia. Each filament was automatically centered and the diameter automatically adjusted. Surface tool was used to manually trace aneurysms to determine their volume. Morphological information was extracted using the statistics tool.

Quantitative real-time PCR

Tissue samples were mechanically homogenized in TRI Reagent (93289, Sigma-Aldrich) according to manufacturer's instructions. RNA was dissolved in Tris-EDTA (pH 7.4) (93302, Sigma-Aldrich) and stored at -80°C. DNA was removed from 1 μg RNA using RQ1 DNaseI (M6101, Promega) following manufacturer's instructions. cDNA was synthesized using the RevertAid RT Reverse Transcription Kit (K1691, Thermo Fisher Scientific) according to manufacturer's instructions. cDNA, forward and reverse primers (added to 0.4 μM), 1x SensiFAST™ SYBR Lo-ROX Kit (BIO-94020, Bioline) were added and topped up to 10 μL using DNase-free water (AHF7114, Baxter). Primers used are listed in Table S5, some which were previously published.[58] Samples were analyzed in a 7500 Fast Real-Time PCR System or QuantStudio™ 7 Real-Time PCR System (Thermo Fisher Scientific) with the cycle program: 95°C for 2 min and then 40 cycles of 95°C for 3s then 60°C for 30s, followed by melt curve analysis. All plates included the relevant no template controls and 18S for normalization.

To detect the *Ifnar1* exon 10 present on genomic DNA (gDNA) relative to *Tk1*, 1 ng of DNA was added with 0.4 μM primers, 1x SensiFAST™ SYBR Lo-ROX Kit and water with the same cycle program as in quantitative real-time PCR section. Primers used are listed in Table S5. Each plate was run with controls of known $Ifnar1^{fl/fl}$ and $Ifnar1^{\Delta Ex10/+}$ DNA which were used to determine whether mice had a half-global deletion of *Ifnar1* exon 10.

Blood-brain barrier leakage

Sodium fluorescein (332 Da; 166308, Sigma-Aldrich) and Evans blue (961 Da; E2129, Sigma-Aldrich) was used to assess blood-brain barrier integrity, as per previous studies.[59,60] Mice were injected i.p. (both markers at 5 ml/kg; both dissolved in 0.9% saline), first with 2% Evans blue and allowed to circulate overnight and subsequently with 2% sodium fluorescein. After 30 min, mice were euthanized and rapidly perfused with ice cold 1x PBS. The brain was removed, briefly washed in ice cold 1x PBS and excess liquid was blotted. Brains were photographed and then bisected along the longitudinal fissure. For histological examinations, one sagittal half brain was coated and frozen in O.C.T. (IA018, ProSciTech) in liquid nitrogen cooled 2-methylbutane (320404, Sigma-Aldrich). Dried cryosections (10 μm) were viewed under fluorescence (DM4000B, Leica) to determine the spatial distribution of blood-brain barrier leakage and acquired using SPOT Advanced v4.5. To quantify leakage of the dyes into the brains, brains were weighed and homogenized in 1x PBS. Protein was precipitated from the supernatant homogenate in 25% trichloroacetic acid at 4°C for 30 min. After pelleting at 10,000 g for 10 min at 4°C, the supernatant was prepared for detection of fluorescein or Evans blue. For fluorescein, NaOH was added to 1.25 M and for Evans blue, ethanol was added to 70%. Standard curves were prepared with fluorescein or Evans blue in the appropriate amount of trichloroacetic acid and NaOH or ethanol. Fluorescein was excited at 492 ± 5 nm and emission detected at 516 ± 5 nm and Evans blue was excited at 618 ± 5 nm and emission detected at 668 ± 5 nm using a Tecan Infinite M1000 Pro plate reader (Thermo Fisher Scientific).

Motor function quantification

Behavioral tests were performed in the Animal Behavioral Facility of The Bosch Institute, The University of Sydney. Mice were habituated in the facility one week before testing and received food and water *ad libitum* with light between 0600 and 1800 hours. Mice were tested at 8 and 16 weeks of age for early and late stage of disease in adulthood, respectively. All behavioral tests were performed between 0830 and 1500 hours. Equipment was thoroughly cleaned with 80% ethanol after each mouse. Mice were habituated to the testing room and atmosphere in their cages for 25 min before each test and were returned to their cages after each test. The rotarod and balance beam was performed similar in Tung et al.[61] For the rotarod (IITC Life Science), mice were habituated on the 70 mm rods for 1 min. The starting speed was 4 rpm and linearly increased to 40 rpm over 5 min. The rotation of the rod made the mice face the black wall. Once the mice fell, the time was recorded to determine impairment of gross motor function. If mice hung onto the rod for a complete revolution, the time at which they reached the bottom was taken as if they were to have fallen. A total of five consecutive trials were performed with 30 s intertrial intervals. For the balance beam, mice were habituated in the enclosed box for 2 min. The mice were then trained on the beam twice by placing mice in front of the door and then one-third of the distance away from the door, with a 30 s interval once the mice entered the box. The test consisted of placing the mice at the starting end of the beam and allowing them to walk into the enclosed box. Video was recorded from above where mice were timed to transverse 60 cm of the beam and another video recorded from behind but at an elevation such that the rear paws were in view to detect foot slips. A total of five trials were conducted with 30 s intertrial interval. Trials were only counted if the mice moved in the one direction and were not counted if they fell off or reversed in direction. A foot slip was considered when one rear paw slid off the beam. Hi-speed video of the gait of the mice was obtained using the ExerGait XL treadmill (Columbus Instruments) and automatically analyzed with TreadScan (CleverSys). Mice were habituated on the treadmill for 1 min before the apparatus was started. Each recording was for 10 s and was analyzed if the mice showed consistent walking determined by TreadScan. Data was obtained on each paw during the analysis and was excluded if the datum was two standard deviations from the mean for that paw. Data from rear paws were averaged as no hemispherical biased changes were previously observed in the brains of GIFN mice.[14,15]

Protein isolation and immunoblot

Tissues were mechanically homogenized in 50 mM Tris-HCl (pH 7.5), 150 mM NaCl, 1 mM EDTA, 1% sodium deoxycholate (D6750, Sigma-Aldrich), 1% Triton-X, 0.1% SDS, 1x Protease Inhibitor Cocktail III (539134, Merck Millipore), 1x Phosphatase Inhibitor Cocktail II (524625, Merck Millipore). The supernatant was collected after centrifugation at 14,000 g at 4°C for 15 min. Protein concentration was determined spectrophotometrically using the Pierce™ BCA Protein Assay Kit (23225, Thermo Fisher Scientific) following manufacturer's instructions. Proteins were denatured in Laemmeli loading buffer [62.5 mM Tris-HCl (pH 6.8), 2% SDS, 10% glycerol, 5% β-mercaptoethanol (0482, Amresco) and 0.002% bromophenol blue] at 95°C for 5 min prior separation in a 10% Tris-glycine polyacrylamide gel and were electrophoretically transferred onto polyvinylidene difluoride membranes (RPN303F, GE Healthcare) in 24.8 mM Tris, 0.19 M glycine and 15% methanol for at 60 V for 3.5 h at 4°C. Total protein was visualized with Ponceau Red S (P7170, Sigma-Aldrich) for 5 min with shaking and destained in water. The membrane was then blocked in either blocking buffer of 5% skim milk (Woolworths Skim Milk Powder, Woolworths) or 5% BSA in tris buffered saline with Tween-20 [TBS-T; 137 mM NaCl, 20 mM Tris-HCl (pH 7.4) and 0.1% Tween-20] for 40 min at 4°C. Primary antibodies [pY701-STAT1 (1:2,000; 7649, CST); pS727-STAT1 (1:2,000; 9177, CST); STAT1 (1:2,000; 9172, CST); GAPDH (1:30,000; MAB374, Merck Millipore)] diluted in blocking buffer were incubated on the membrane overnight at 4°C with shaking. The membrane was washed with in TBS-T prior to incubation

with the peroxidase conjugated secondary antibody [anti-rabbit horseradish peroxidase (1:30,000; SC2004, SCT); anti-mouse peroxidase (1:10,000; A0168, Sigma-Aldrich)] for 1 h. After washing with TBS-T, proteins were revealed using Immobilon Western Chemiluminescent HRP Substrate (WBKLS0500, Merck Millipore) and visualized on film (34091, Thermo Fisher Scientific) or the ChemiDoc XRS+ (BioRad Laboratories) with multiple exposure times to get non-saturated bands. To re-probe the membrane for different targets, antibodies were stripped in 62.5 mM Tris-HCl (pH 6.8), 2% SDS and 100 mM β-mercaptoethanol for 25 min at 60°C and then rinsed in deionized water before blocking and re-probing. Densitometric quantifications were performed on scanned films or image files using Fiji[50,51] and intensities were normalized against GAPDH. For cross membrane comparisons, gels were run at the same time, transferred in the same tank, blocked, probed, stripped and exposed together. Moreover, one sample on each gel was identical and was used to normalize bands between membranes.

IFN-α ELISA

From WT (N = 4) and GIFN (N = 8) mice, CSF from the cisterna magna was collected and placed on ice. Ventricular blood was collected using heparinized (1000 U/ml) 25 G needle and 1 ml syringe and placed on ice. Plasma was isolated by centrifugation of blood at 2,000 g at 4°C. IFN-α_1 was detected by ELISA (ab252352, Abcam) following manufacturer's instructions with plasma diluted 1:2 and CSF from WT mice was diluted 1:12 (due to the low CSF volume and minimum sample input volume) and 1:20 from GIFN mice. All samples from WT mice were below the detection limit of the ELISA.

Cell type validation of recombination

To verify recombination specificity, we dissociated and sorted cells of the brain. Briefly, mice were transcardially perfused with ice cold 1x DBPS and the forebrain processed using the Adult Brain Dissociation Kit. Cells were blocked in CD16/32 (1:100; 553142, BD Biosciences) and LIVE/DEAD Fixable Blue Dead Cell Stain Kit (1:500; L34961, Thermo Fisher Scientific) in 1x PBS on ice in the dark. After pelleting at 300 g for 5 min at 4°C, cells and compensation beads were resuspended in 5% FBS and 5 mM EDTA in 1x PBS containing a mixture of antibodies for 1 h on ice in the dark. Antibodies used were CD31-BV421 (1:200; 102423, Biolegend), CD11b-BV650 (1:200; 101239, Biolegend), CD45-FITC (1:100; 553080, BD Biosciences), ACSA-2-PE (1:100; 130-123-284, Miltenyi Biotec) and CD90.2-APC (1:200; 561974, BD Biosciences). Controls of unstained cells, single-stained compensation beads (01-2222-41, Thermo Fisher Scientific) and fluorescence minus ones were included. Cells were washed at 300 g for 5 min at 4°C before passing through a 35 μm filter. Sorting was performed on the BD Influx 7L with a 100 μm nozzle at Sydney Cytometry. Cells were sorted into four populations which were positive for the indicated markers and negative for all others: astrocytes (ACSA-2$^+$, including those that are also CD90.2$^+$), endothelial cells (CD31$^+$), microglia (CD45$^+$CD11b$^+$) and neurons (CD90.2$^+$). Volume was removed to ensure <5x10^5 cells and then was pelleted at 300 g for 5 min at 4°C. Supernatant was removed and the pellet was stored at -80°C before RNA extraction using the RNeasy Micro Kit (74004, Qiagen) following manufacture's instruction with the inclusion of β-mercaptoethanol and DNaseI digestion. To verify recombination specificity in hematopoietic cells, blood was collected in heparinized cannula and syringes. Red blood cells were lysed using ammonium chloride (07850, Stemcell Technologies) as per manufacturer's instructions. Cells were pelleted at 500 g for 5 min and washed with 1x DPBS before pelleting. The pellet was lysed with TRI Reagent and RNA isolated according to manufacturer's instructions. cDNA was generated and the maximum volume was used in the qPCR reaction, as noted in the kit's instructions; method details described in quantitative real-time PCR section. For controls, cortical brain samples from WT mice and mice with excision of *Ifnar1* exon 10 from all cells were included.

Phagocytosis assay of primary murine microglia

Primary microglia were pre-treated with DF media or 50 U/ml IFN-α (130-093-130, Miltenyi Biotech) for a total of 72 h with treatment being changed every 24 h. To measure phagocytic activity, yellow-green fluorescent carboxylate-modified polystyrene latex beads (L4655, Sigma-Aldrich) were added to cells at 200 beads per cell and incubated for 1 h at 37°C with 5% CO_2 in a humified incubator. To differentiate phagocytosis from beads attached to cells, cells were kept at 4°C for 1-2 h prior to adding the beads and throughout the assay. Following washing, cells were fixed with 10% neutral buffered formalin for 20 min. Fixed cells were washed in PBS prior to detection on the 5-laser Aurora Spectral cytometer (Cytek Biosciences) at Sydney Cytometry. Data was anaylzed with FlowJo. Quality control gating for time, single cells and debris.

QUANTIFICATION AND STATISTICAL ANALYSIS

Statistical analyses of different data sets are outlined in Figure legends and were performed in GraphPad Prism (version 8), or R. Statistical analyses for human paired blood-CSF samples were performed using Wilcoxon paired signed rank tests and descriptive statistics used median with interquartile range. Two-sided Mann-Whitney U test was used to compare between groups.

For mouse studies, assumptions of normality of residuals and homogeneity of variance were examined with Q-Q and homoscedasticity plots, respectively. Briefly, comparisons between genotypes were performed with one-way ANOVA with Tukey's post-test, comparisons between age and genotypes were performed by two-way ANOVA and repeated measures linear mixed-effects models with Tukey's post-test for motor function analyses. Data on number of foot slips underwent square-root transformation and quantification of blood leakage underwent log transformation to obtain a normal distribution and

homogeneity of variance. Pairwise comparison of survival curves by log-rank test, adjusted with a Benjamini-Hochberg procedure, was done using the ‘survminer’ package[38] in R. Statistical analyses involving repeated measures of ordinal data was analyzed with a cumulative link mixed model fitted with the Laplace approximation with the ‘ordinal’ package.[39] Statistical analysis of repeated measures weight data with missing data was performed with linear mixed-effects models using the ‘lme4’ package.[40] Pairwise comparisons with Tukey’s P-value adjustment were performed using the ‘emmeans’ package.[41] A $P \leq 0.05$ was considered significant.

Immunity, Volume *57*

Supplemental information

The brain microvasculature is a primary mediator of interferon-α neurotoxicity in human cerebral interferonopathies

Barney Viengkhou, Emina Hayashida, Sarah McGlasson, Katie Emelianova, Deborah Forbes, Stewart Wiseman, Joanna Wardlaw, Rovin Verdillo, Sarosh R. Irani, Darragh Duffy, Fredrik Piehl, Lipin Loo, Axel Pagenstecher, G. Greg Neely, Yanick J. Crow, Iain L. Campbell, David P.J. Hunt, and Markus J. Hofer

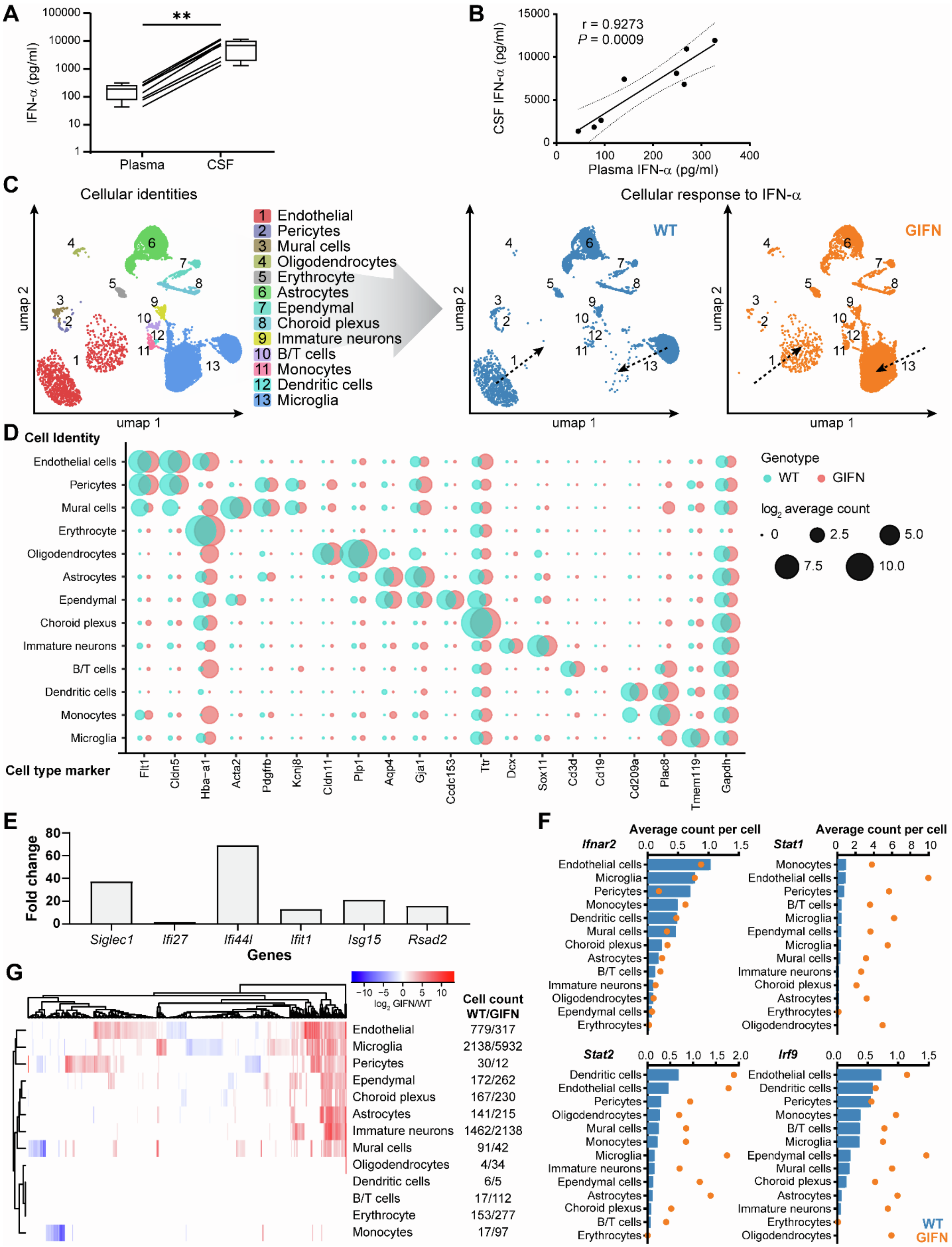
A
IFN-α (pg/ml)
Plasma
CSF
**
B
r = 0.9273
P = 0.0009
CSF IFN-α (pg/ml)
Plasma IFN-α (pg/ml)
C
Cellular identities
1 Endothelial
2 Pericytes
3 Mural cells
4 Oligodendrocytes
5 Erythrocyte
6 Astrocytes
7 Ependymal
8 Choroid plexus
9 Immature neurons
10 B/T cells
11 Monocytes
12 Dendritic cells
13 Microglia
Cellular response to IFN-α
WT
GIFN
umap 1
umap 2
D
Cell Identity
Endothelial cells
Pericytes
Mural cells
Erythrocyte
Oligodendrocytes
Astrocytes
Ependymal
Choroid plexus
Immature neurons
B/T cells
Dendritic cells
Monocytes
Microglia
Cell type marker
Flt1
Cldn5
Hba-a1
Acta2
Pdgfrb
Kcnj8
Cldn11
Plp1
Aqp4
Gja1
Ccdc153
Ttr
Dcx
Sox11
Cd3d
Cd19
Cd209a
Plac8
Tmem119
Gapdh
Genotype
WT
GIFN
log2 average count
0
2.5
5.0
7.5
10.0
E
Fold change
Siglec1
Ifi27
Ifi44l
Ifit1
Isg15
Rsad2
Genes
F
Average count per cell
Ifnar2
Stat1
Stat2
Irf9
WT
GIFN
G
log2 GIFN/WT
Cell count WT/GIFN
Endothelial 779/317
Microglia 2138/5932
Pericytes 30/12
Ependymal 172/262
Choroid plexus 167/230
Astrocytes 141/215
Immature neurons 1462/2138
Mural cells 91/42
Oligodendrocytes 4/34
Dendritic cells 6/5
B/T cells 17/112
Erythrocyte 153/277
Monocytes 17/97

Fig. S1 CSF IFN-α concentrations and type I interferon signaling are increased in brains of GIFN mice (Fig. S1 refers to Fig. 2).

(**A**) Paired comparison of CSF and plasma samples IFN-α protein from GIFN mice ($n = 8$) measured by ELISA. Samples from WT mice ($n = 4$) were below the detection limit of the ELISA. Box plots are shown. Paired two-tailed t test. $^{**}P < 0.01$.

(**B**) Correlation between CSF and plasma IFN-α protein in GIFN mice. Pearson correlation with 95% confidence bands.

(**C**) UMAP plot showing clustering of cells in of WT and GIFN mice. Data from one experiment.

(**D**) Known cell-type-associated markers were used to assign and verify cell identities of clusters.

(**E**) Fold change (GIFN vs WT) of genes used to calculate the whole brain ISG score.

(**F**) Average count of transcripts of each cell identity and between WT and GIFN cells. *Ifnar1* is shown in Fig. 2.

(**G**) Heatmap of type I interferon-regulated genes from each cell identity.

A-B: Representative data from two independent experiments. **C-G**: Analyzed data from one experiment.

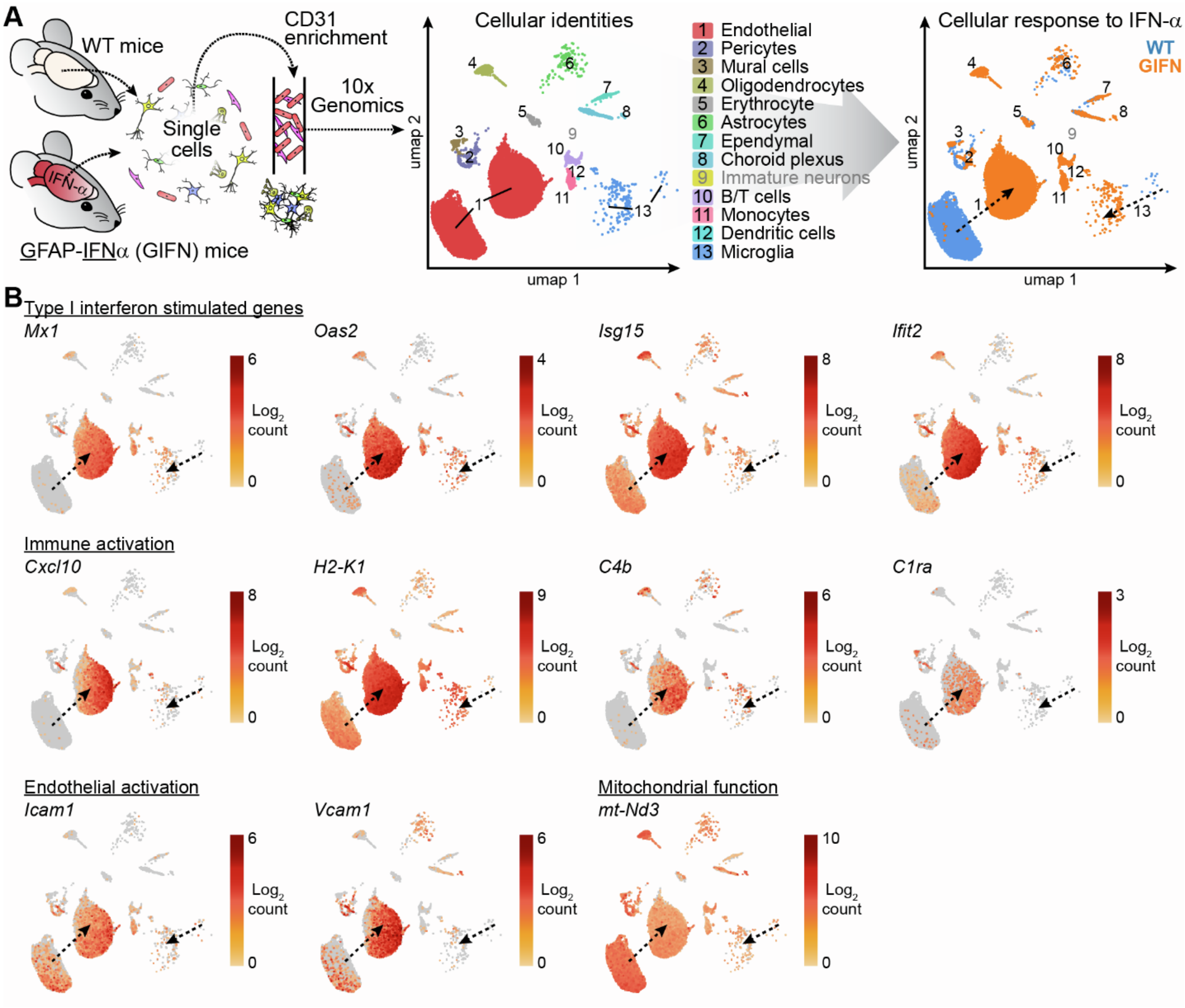

Fig. S2. Immune stimulation and endothelial cell activation is present in CD31-enriched cortical cells from GIFN mice versus WT mice (Fig. S2 refers to Fig. 3A-B).

(**A**) Single-cell RNA sequencing of isolated cells from pooled forebrains of WT and GIFN mice that were enriched using CD31-microbeads. Cells were clustered and assigned an identity which allowed transcriptomic comparison between the two genotypes, as in Fig. 2. Note, immature neurons were not detected. Dashed arrows indicate shift in endothelial cells and microglia between WT and GIFN mice.

(**B**) Markers of canonical type I interferon-stimulated genes, immune activation, endothelial activation and mitochondrial function.

A-B: Data from one experiment.

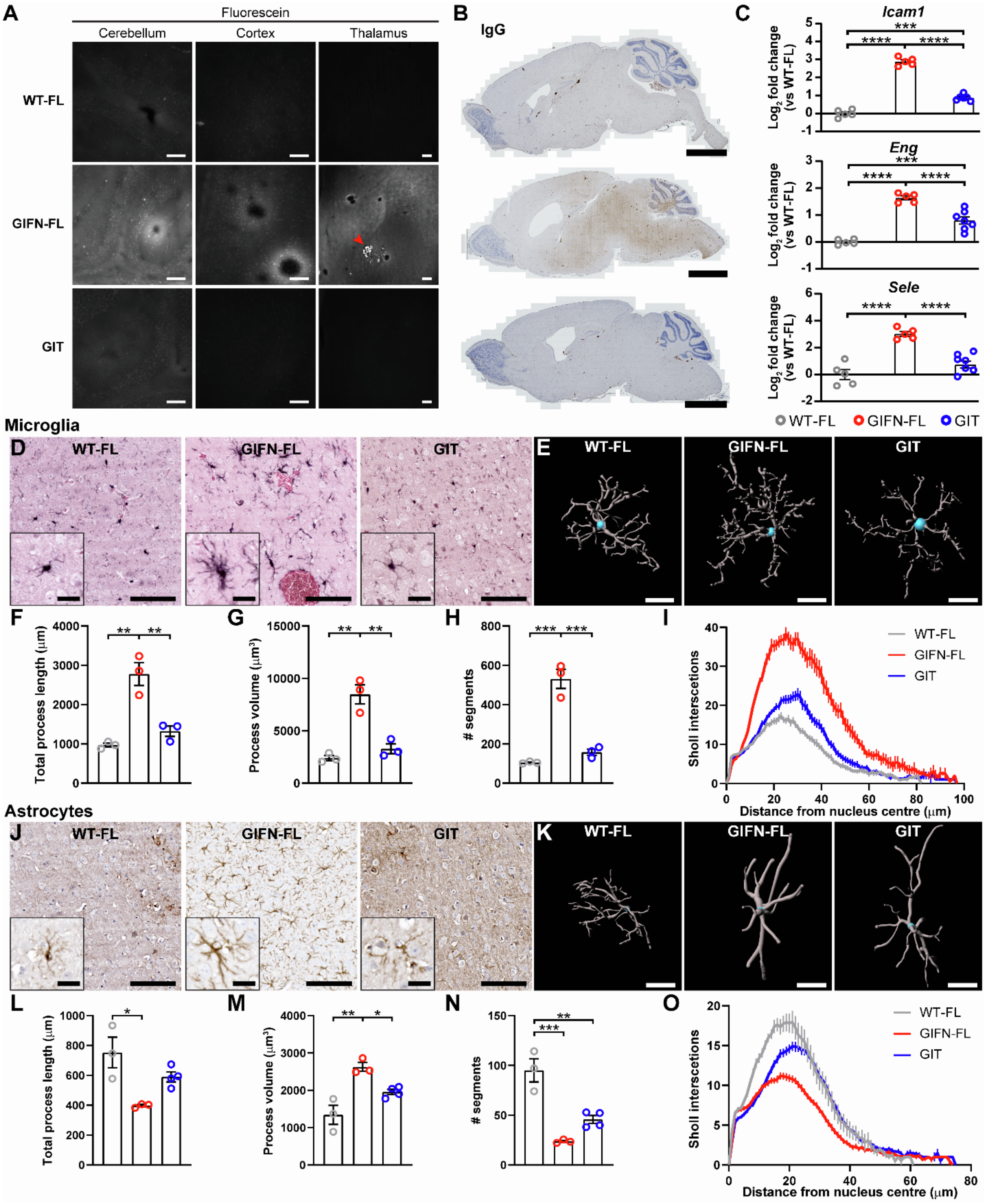

A
Fluorescein
Cerebellum
Cortex
Thalamus
WT-FL
GIFN-FL
GIT
B
IgG
C
Icam1
Eng
Sele
Log2 fold change (vs WT-FL)
WT-FL
GIFN-FL
GIT
Microglia
D
E
F
G
H
I
Total process length (μm)
Process volume (μm3)
segments
Sholl interscetions
Distance from nucleus centre (μm)
Astrocytes
J
K
L
M
N
O

Fig. S3. Blood-brain barrier function is restored and morphological characteristics of cortical microglia and astrocytes is partially reversed in GIT mice (Fig. S3 refers to Fig. 6).

(**A)** Representative fluorescent images of brain sections for fluorescein revealing the spatial leakage (*n* = 4 per genotype at 16 weeks of age). Red arrowhead: calcification; scale bar, 100 μm. Representative images from over three independent experiments.

(**B**) Immunohistochemistry for IgG (brown) in the parenchyma (*n* = 4 per genotype at 16 weeks of age; scale bar, 2 mm; hematoxylin counterstain). Representative images from two independent experiments.

(**C**) Markers of endothelial activation in the cerebellum measured by qPCR (*n* = 5 for WT-FL and GIFN-FL and *n* = 7 for GIT). Mean ± s.e.m. are shown. One-way ANOVA with Tukey's post-test. $**P < 0.01$, $***P < 0.001$ and $****P < 0.0001$. Quantification was one experiment from sample collected from more than three independent experiments.

(**D** and **J**) Immunohistochemistry for microglia (Iba1) and astrocytes (GFAP) (*n* = 4 per genotype at 16 weeks of age; scale bar, 100 μm and 20 μm for inserts). Representative images from two independent experiments.

(**E** and **K**) Reconstructed morphology from Iba1 and GFAP stained passively cleared brains (*n* = 3-4 mice per genotype at 16 weeks of age). Representative images from two independent experiments.

(**F** and **L**) Total length of processes calculated from morphological reconstructions.

(**G** and **M**) Average volume of processes calculated from single cell morphological reconstructions.

(**H** and **N**) Number of segments calculated from single cell morphological reconstructions.

(**I** and **O**) Histogram of Sholl intersections calculated from single cell morphological reconstructions.

C, **F**-**H** and **L**-**N**: each point is a mouse. **I** and **O** are from of all measured microglia and astrocytes. Mean ± s.e.m. are shown. Statistical comparisons were performed using one-way ANOVA with Tukey's post-test. $*P \leq 0.05$, $**P \leq 0.01$, $***P \leq 0.001$ and $****P \leq 0.0001$. **E-I** and **K-O**: Quantification of data pooled from two independent experiments.

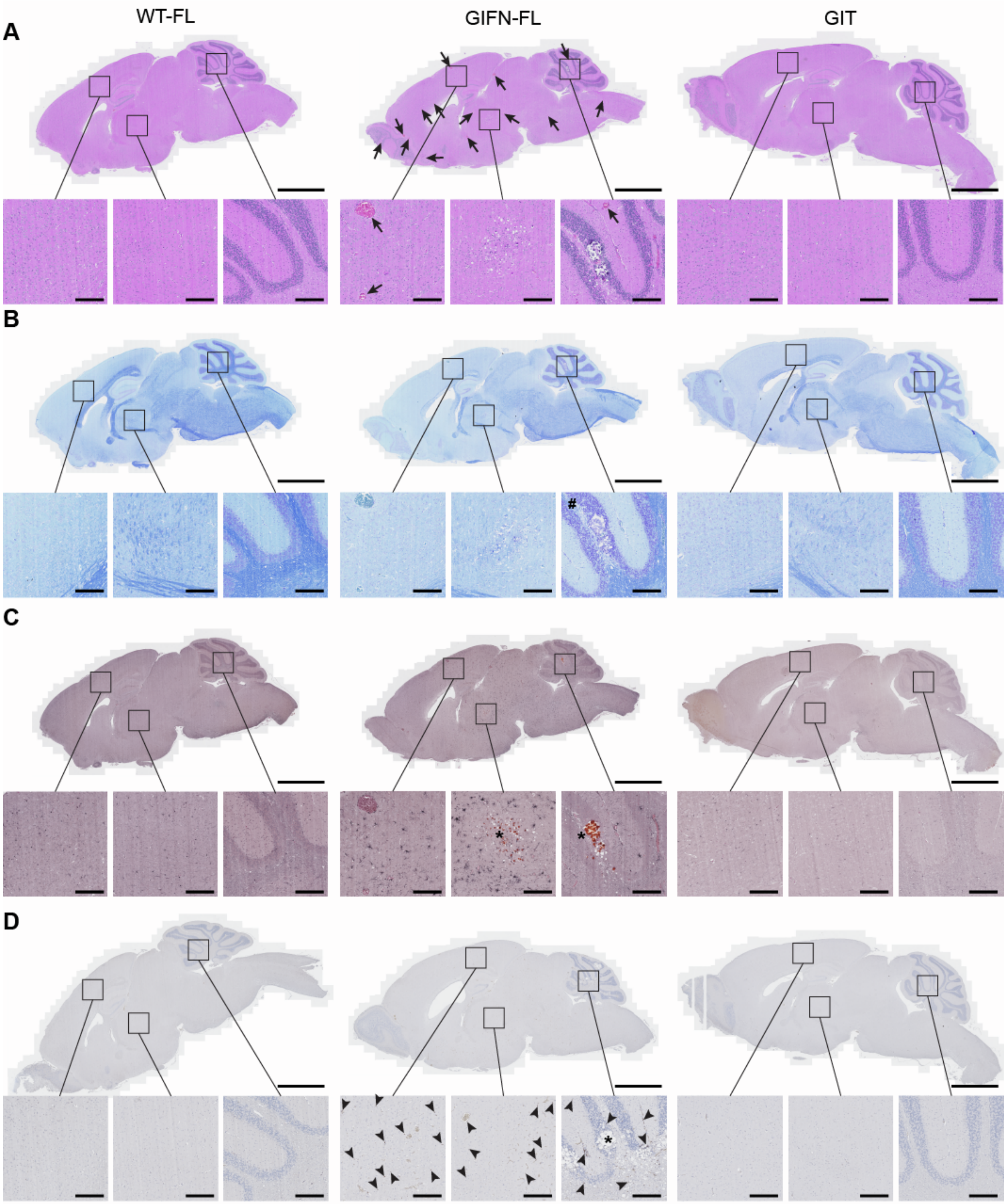

Fig. S4. Microangiopathy, neurodegeneration, calcification and T cell infiltrates are absent in the brains of 16-week-old GIT mice (Fig. S4 refers to Fig. 7A).

(**A**) H&E (arrows: aneurysms).

(**B**) LFB&CV (#: loss of myelin).

(**C**) Iba1-ARS (*: calcification).

(**D**) CD3 (arrowheads) staining.

Staining of of WT-FL, GIFN-FL and GIT mice at 16 weeks of age (n = 4 per genotype). Whole brain image: scale bar, 2 mm; zoomed images: scale bar, 200 μm. Representative images from two independent experiments.

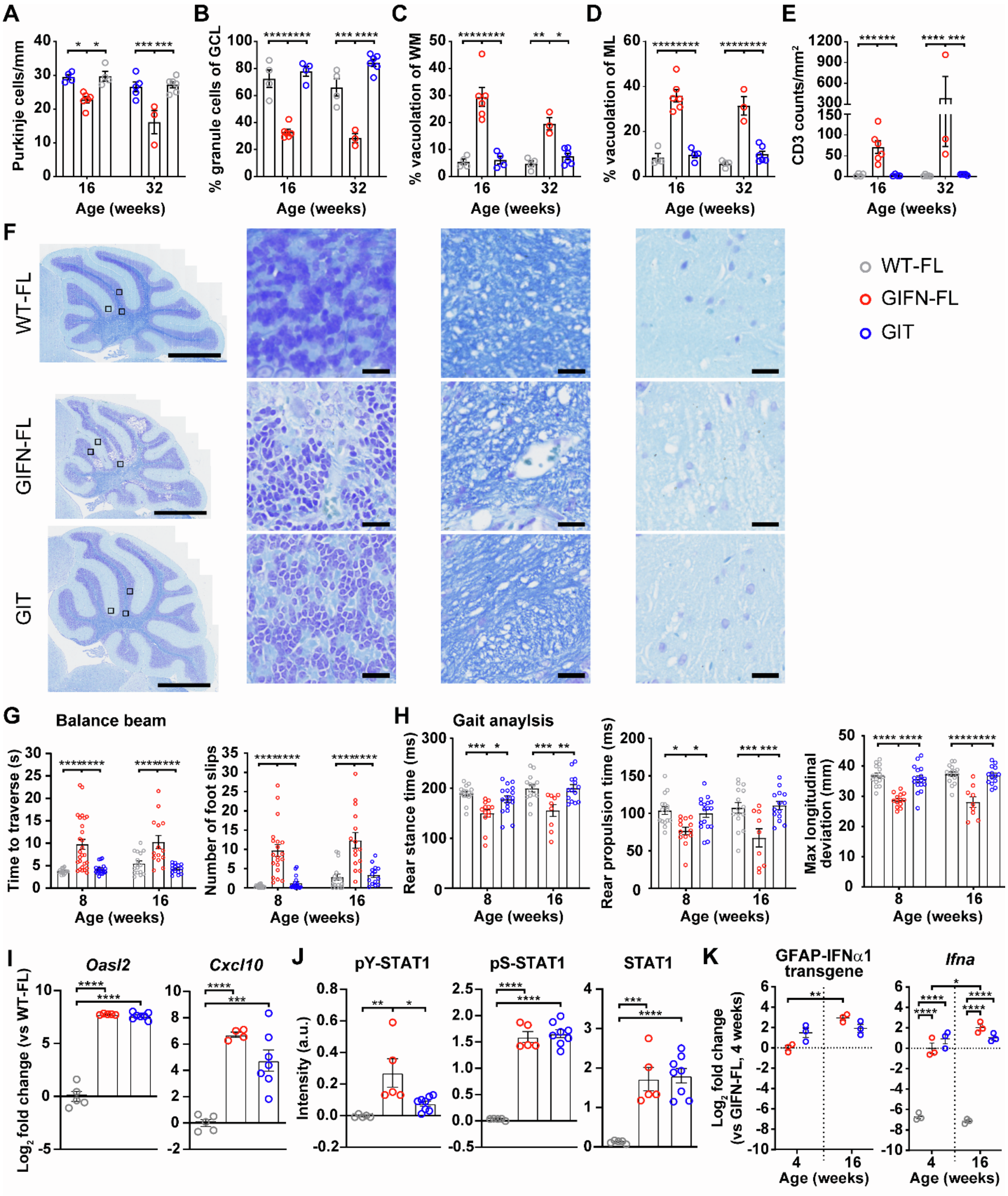

Fig. S5. Neuropathology and motor phenotype are improved in GIT mice despite chronic type I interferon signaling in the brain (Fig S5 refers to Fig. 7).

(**A**) Purkinje cells normalized to the perimeter of the granule cell layer (GCL) of the cerebellum.

(**B**) Percentage area granule cells in the GCL of the cerebellum.

(**C**) Percentage area vacuolation of the white matter (WM) of the cerebellum.

(**D**) Percentage area vacuolation of the molecular layer (ML) of the cerebellum.

(**E**) Number of CD3 cells per mm^2 of the cerebellum.

(**F**) Representative low (scale bar, 1 mm) and high magnification (scale bar, 20 µm) of the cerebellum indicating the GCL, WM and ML in WT-FL, GIFN-FL and GIT mice at 16 weeks of age (*n* = 4 per genotype).

(**G**) Fine motor coordination detected using the balance beam test (*n* = 16 for WT-FL, 25 for GIFN-FL and 18 for GIT at 8 weeks of age; 16 for WT-FL, 12 for GIFN-FL and 14 for GIT at 16 weeks of age; repeated measures linear mixed-effects models with Tukey's post-test; also viewed in Video S2).

(**H**) Parameters of gait of average duration in which rear paws are in contact with the treadmill, average time it takes to leave the stance phase and average maximum distance the foot deviates from the waist during the stance phase (*n* = 15 for WT-FL, 13 for GIFN-FL and 17 for GIT at 8 weeks of age; 14 for WT-FL, 10 for GIFN-FL and 14 for GIT at 16 weeks of age; repeated measures linear mixed-effects models with Tukey's post-test).

(**I**) Expression of type I interferon-stimulated genes in the cerebella of mice measured by qPCR (*n* = 4-5 for WT-FL and GIFN-FL and 7 for GIT at 16 weeks of age; one-way ANOVA with Tukey's post-test).

(**J**) Quantification of immunoblots from Fig. 7D (*n* = 5 for WT-FL and GIFN-FL and 8 for GIT at 16 weeks of age; one-way ANOVA with Tukey's post-test).

(**K**) Expression of the transgene (GFAP-IFNα1) and *Ifna* in the cerebellum measured by qPCR (*n* = 3 per genotype per age; two-way ANOVA with Tukey's post-test). Representative analysis from two independent experiments.

Points represent individual animals and mean ± s.e.m. shown. *$P < 0.05$, **$P < 0.01$, ***$P < 0.001$ and ****$P < 0.0001$. **A**-**E**: *n* = 3-5 per genotype per age. Two-way ANOVA with Tukey's post-test. **A**-**G**: Quantification from images obtained in two independent experiments. **F**: Representative images from two independent experiments. **G**-**H**: Data pooled from more than three independent experiments. **I**-**J**: Quantification was one experiment from sample collected from more than three independent experiments.

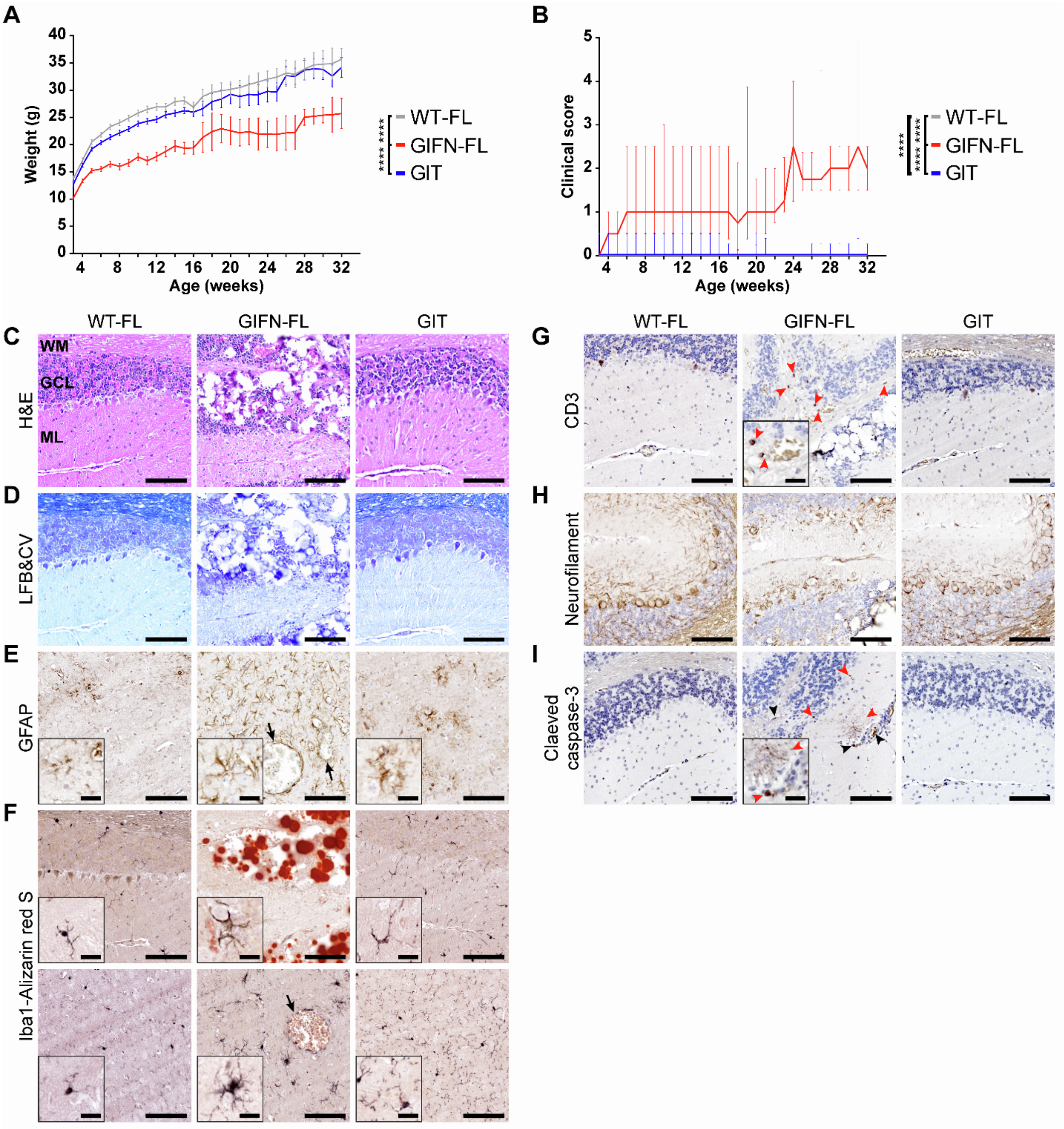

Fig. S6. GIT mice have a largely WT-like development and neuropathology (Fig S6 refers to Fig. 7).

(**A**) Weight curve of mice up to 32 weeks of age (*n* = 50-57 mice per genotype). Mean ± s.e.m. shown.

(**B**) Clinical score (median ±interquartile range shown) indicating development of ataxia (score: 0.5 – 2), seizures (score: 3 – 4) and mortality (score: 5). *n* = 50-57 mice per genotype. Significance of clinical scores between genotypes was calculated with cumulative link mixed models with *P* adjusted with Tukey's post-test. Significance of weight between

genotypes was calculated with linear mixed-effects models and P adjusted with Tukey's post-test. ****$P \leq 0.0001$.

(**C**) H&E staining (white matter (WM), granule cell layer (GCL) and molecular layer (ML)).

(**D**) LFB&CV staining.

(**E**) GFAP immunohistochemistry.

(**F**) Staining for Iba1 and Alizarin red S.

(**G**) CD3 staining showed T cell infiltration (red arrowheads). Black arrowheads indicate perivascular cells and red arrow heads indicate positive cells in the parenchyma.

(**H**) Neurofilament staining.

(**I**) Cleaved caspase-3 staining showed apoptosis.

A-B: Data pooled from more than three independent experiments. **C-I**: n = 3-4 per genotype at 32 weeks of age. Scale bar, 100 µm and 20 µm in inserts. Representative images from two independent experiments.

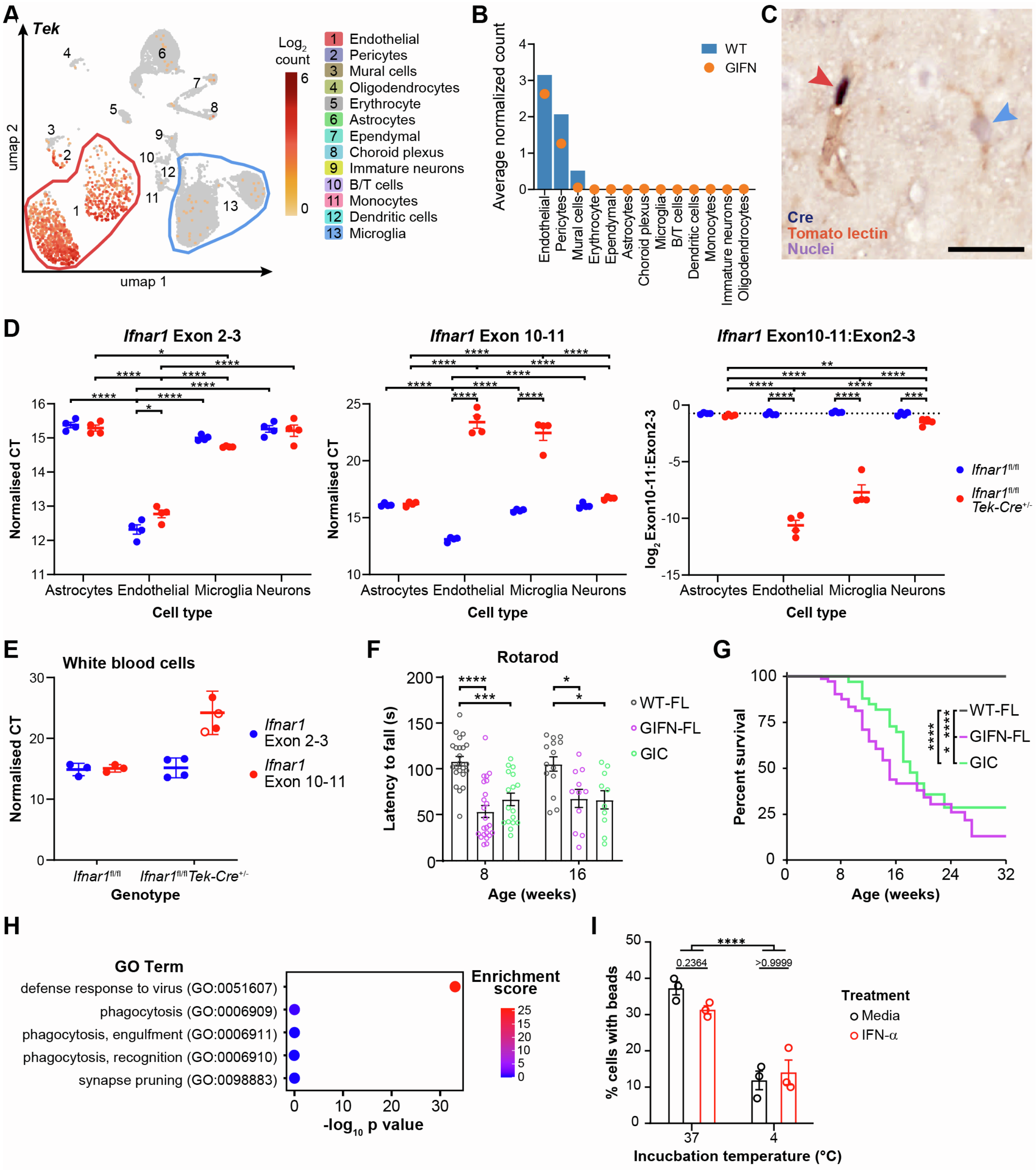

Fig. S7. *Ifnar1* deletion on microglia does not rescue clinical phenotype in GIFN mice (Fig S7 refers to Figs. 6 & 7).

(**A**) scRNASeq data from WT and GIFN mice showing *Tek* expression.

(**B**) Average normalized counts of *Tek* for each cell identity.

(**C**) Immunohistochemistry for Cre recombinase protein and tomato lectin (labels endothelial cells and microglia). Endothelial cell nuclei with Cre recombinase (red arrowhead), but

not in microglia (blue arrowhead). Scale bar, 20 μm. Representative images from two independent experiments.

(**D** and **E**) Expression of *Ifnar1* mRNA in (**D**) different brain cell types from *Ifnar1*$^{fl/fl}$ and *Ifnar1*$^{fl/fl}$*xTek-Cre*$^{+/-}$ mice (n = 4 per genotype) and (**E**) in white blood cells (n = 3-4 per genotype) measured by qPCR. Primers amplifying across exons 2 and 3 indicate levels of *Ifnar1* transcripts and those amplifying across exons 10 and 11 indicate transcripts from cells without exon 10 excision. Transcripts were normalized to 18S. Mean ± s.e.m. and two-way ANOVA with Tukey's post-test are shown in (**D**) and mean ± s.d. in (**E**). Empty dots in (**E**) were not included in the mean but indicate undetermined CTs that were set to a CT value of 40. **D**: Data were from two independent experiment. **E**: Data from one experiment.

(**F**) Gross motor function measured by rotarod test (n = 23 for WT-FL, 23 for GIFN-FL and 17 for GIC at 8 weeks and n = 14 for WT-FL, 11 for GIFN-FL and 10 for GIC at 16 weeks; repeated measures linear mixed-effects models with Tukey's post-test. Quantification of data pooled from more than three independent experiments.

(**G**) Survival analysis of mice with *Ifnar1* excision in microglia in GIFN mice (GIC) and control littermates (total n = 96 for WT-FL, 82 for GIFN-FL and 47 for GIC mice). Significance determined by long-rank test with Benjamini-Hochberg post-test. Data pooled from more than three independent experiments.

(**H**) Overrepresentation analysis for phagocytosis associated biological processes in microglia from the GIFN brain using PANTHER.

(**I**) Phagocytosis assay using fluorescent latex beads incubated for 1 h at 4°C or 37°C with primary microglia pretreated with media or IFN-α for 72 h (n = 3 per treatment per temperature). Mean ± s.e.m. and two-way ANOVA with Tukey's post-test. Representative analysis from two independent experiments.

A-B and **H**: Data from one experiment. *$P < 0.05$, **$P < 0.01$, ***$P < 0.001$ and ****$P < 0.0001$.

Table S1. IFN-α concentrations in paired serum-CSF across diseases (Table S1 refers to Fig 1A-D).

	**Controls**		**Interferonopathic Disease**	
	**Healthy Control**	**Disease Control (Multiple Sclerosis – a non-interferonopathic disease)**	**Cerebral Interferonopathy disease (AGS)**	**Systemic Interferonopathy disease (SLE)**
**Number of serum-CSF pairs**	19	32	29	53
**Serum IFN-α fg/ml (median and IQR)**	0.36 (0.36 - 0.40)	0.36 (0.36 - 0.62)	153 (39 - 622)	195 (9.9 - 770)
**CSF IFN-α fg/ml (median and IQR)**	1.74 (1.1 - 4.1)	0.81 (0.72 - 2.1)	552 (104 - 3189)	7.3 (1.5 - 38.5)

Table S3. Multiple linear regression analysis with "age at sampling" as the dependent variable (Table S3 refers to Fig. 5A).

Parameter estimates	Variable	Estimate	|t|	P value	VIF	R2 with other variables
β0	Intercept	38.16	7.486	<0.0001		
β1	CSF interferon (IU/ml)	-0.2281	3.628	0.0004	1.085	0.07818
β2	Serum interferon (IU/ml)	-0.08033	0.7658	0.4456	1.085	0.07818

Table S4. Cohort demographics for human samples.

A) Cohort demographic for paired serum-CSF Simoa study (Table S4A refers to Fig 1).

B) Cohort demographic for SLE paired Simoa – neuroimaging/MRI study (Table S4B refers to Fig 5).

Table S4A.

		Non-interferonopathic controls		Interferonopathic disease		
Underlying diagnosis		Healthy controls	Relapsing-remitting multiple sclerosis	Systemic lupus erythematosus cohort 1	Systemic lupus erythematosus cohort 2	Aicardi-Goutières Syndrome
Number of serum-CSF pairs		19	32	35	18	29
Age	Mean age	27.4	34.5	45	15	9
	Age range	22-32	20-51	18-77	11 to 17	0-38
Sex	Female %	53	78	97	73	54
Ethnicity	White Caucasian %	89	84	86	N/A	N/A
	Asian %	11	16	14	N/A	N/A
Country of origin	Sweden %	78	72	80	N/A	N/A
	Other European %	11	6.00	9	N/A	N/A
	Middle East %	11	16	9	N/A	N/A
	Other %		6	2	N/A	N/A

Table S4B.

Diagnosis		Systemic lupus erythematosus
	n	47
Age	Mean age	49
	Age range	20-75
Sex	Female %	91
Ethnicity	White Caucasian %	93
	Other %	7
Socioeconomic status	School leaving age	16 years

Table S5. List of oligonucleotides (Table S5 refers to STAR Methods).

Oligonucleotide	Source	Identifier
Expression assays		
Icam1 forward primer: 5’-CTGGGCTTGGAGACTCAGTG-3’	Sigma-Aldrich	N/A
Icam1 reverse primer: 5’-CACACTCTCCGGAAACGAA-3'	Sigma-Aldrich	N/A
Eng forward primer: 5’-AGGTGTTCCTGGTCCTCGTT-3’	Sigma-Aldrich	N/A
Eng reverse primer: 5’-GTTGACTCTTGGCTGTCCTTG-3'	Sigma-Aldrich	N/A
Sele forward primer: 5’-CTAGCGCCTGGATGAAAGC-3'	Sigma-Aldrich	N/A
Sele reverse primer: 5’-GAGCTCACTGGAGGCATTGT-3'	Sigma-Aldrich	N/A
Isg15 forward primer: 5’-GAGCTAGAGCCTGCAGCAAT-3'	Sigma-Aldrich	N/A
Isg15 reverse primer: 5’-TTCTGGGCAATCTGCTTCTT-3'	Sigma-Aldrich	N/A
Mx1 forward primer: 5’-TCTGAGGAGAGCCAGACGAT-3'	Sigma-Aldrich; Hernández et al. [54]	N/A
Mx1 reverse primer: 5’-ACTCTGGTCCCCAATGACAG-3'	Sigma-Aldrich; Hernández et al. [54]	N/A
Oasl2 forward primer: 5’-GGATGCCTGGGAGAGAATCG-3'	Sigma-Aldrich; Hernández et al. [54]	N/A
Oasl2 reverse primer: 5’-TCGCCTGCTCTTCGAAAC-3'	Sigma-Aldrich; Hernández et al. [54]	N/A
Cxcl10 forward primer: 5’-AGAGACATCCCGAGCCAA-3'	Sigma-Aldrich	N/A
Cxcl10 reverse primer: 5’-GATGAGGCAGAAAATGACGG-3'	Sigma-Aldrich	N/A
Ifna forward primer: 5’-GTGACCTTCCTCAGACTCATAAC-3'	Sigma-Aldrich	N/A
Ifna reverse primer: 5’-CAAAGTCCTTCCTGTCCTTCA-3'	Sigma-Aldrich	N/A
GFAP-IFNα1 transgene forward primer: 5’-CAATGTGCTGGGAAGACTGA-3'	Sigma-Aldrich	N/A
GFAP-IFNα1 transgene reverse primer: 5’-CTGCATTCTAGTTGTGGTTTGTC-3'	Sigma-Aldrich	N/A
Ifnar1 Exon 2 forward primer: 5’-CAGCCACGGAGAGTCAATG-3’	Sigma-Aldrich	N/A
Ifnar1 Exon 3 reverse primer: 5’-GCACTTTTAACCACTTCGCC-3’	Sigma-Aldrich	N/A
Ifnar1 Exon 10 forward primer: 5’-GCCTCCCCGCAGTATTGAT-3’	Sigma-Aldrich	N/A

Ifnar1 Exon 11 reverse primer: 5'-ATCTTTCCGTGTGCTCCTCA-3'	Sigma-Aldrich	N/A
18S forward primer: 5'-CACGGCCGGTACAGTGAAAC-3'	Sigma-Aldrich; Hernández et al. [54]	N/A
18S reverse primer: 5'-AGAGGAGCGAGCGACCAA-3'	Sigma-Aldrich; Hernández et al. [54]	N/A
Genotyping assays		
Ifnar1 Exon 10 forward primer: 5'-GTGTTGTGTTCTTCTCTGTCATGGT-3'	Sigma-Aldrich	N/A
Ifnar1 Exon 10 reverse primer: 5'-AATACTGCGGGGAGGCTTGA-3'	Sigma-Aldrich	N/A
Tk1 forward primer: 5'-CCAACGAGGGCAAGACAGTAATTG-3'	Sigma-Aldrich	N/A
Tk1 reverse primer: 5'-AGAGCACCCACTAGCCAATAGC-3'	Sigma-Aldrich	N/A
